# Genetic background influences developmental airway smooth muscle program and susceptibility to airway hyperresponsiveness in mice

**DOI:** 10.1172/jci.insight.200486

**Published:** 2026-07-21

**Authors:** Takehiro Otoshi, Benjamin D. Kotton, Ayyappa K.S. Kameshwar, Yoshinori Seki, Zachary Cardell, Xiangyi Ke, Yuta Matsuno, Pooja Rajaram, Youn-Kyung Kim, Sarah M. Sharpton, Loredana Quadro, Wellington V. Cardoso, Masako Suzuki

**Affiliations:** 1 Columbia Center for Human Development, Department of Medicine, Division of Pulmonary Allergy and Critical Care Medicine, and Department of Genetics and Development, Columbia University, New York, New York, USA.; 2 Department of Nutrition, Texas A&M University, College Station, Texas, USA.; 3 Department of Genetics, Albert Einstein College of Medicine, Bronx, New York, USA.; 4 Department of Food Science and Rutgers Center for Lipid Research, and New Jersey Institute for Food, Nutrition, and Health, Rutgers University, New Brunswick, New Jersey, USA.; 5 Molecular Genomics Core, Institute for Genome Sciences and Society, Texas A&M University, College Station, Texas, USA.

**Keywords:** Development, Pulmonology, Asthma, Embryonic development, Genetic variation

## Abstract

Airway structural remodeling and hyperresponsiveness (AHR), hallmarks of asthma, are influenced by genetic variations and adverse exposures. While intrauterine perturbations in lung development have been linked to adult pulmonary disease, the developmental origins of these abnormalities remain poorly understood. Here, we provide evidence of genetic background playing a key role in this process. Using A/J and C57BL/6J mice known for their distinct susceptibility to AHR, we show that A/J embryos selectively develop an aberrant airway smooth muscle (SM) program and AHR in adulthood when exposed transiently to a vitamin A/retinoic acid (RA)–disrupted intrauterine environment in vivo by maternal BMS493 administration. Single-nucleus multiomics identified a mesenchymal cell population overactivating TGF-β targets in response to BMS493 selectively in A/J lungs. These cells, localized to sites of airway SM initiation and p-SMAD2- and -3, exhibited robust BMS493-mediated upregulation of SMAD2/3 targets, including regulators of SM program *Pdgfra* and *Tnc*. Functional analyses in vivo and cultured lungs showed aberrant SM formation in areas of overactive TGF-β of BMS493-exposed lungs. These abnormalities were prevented by inhibiting TGF-β signaling in utero in RA-deficient embryos. These findings underscore how distinct genetic backgrounds respond to intrauterine perturbations that program airway structure and function, with potential lasting consequences in postnatal pulmonary function.

## Abstract 2

Genetic background influences airway smooth muscle differentiation and susceptibility to airway hyperresponsiveness after prenatal retinoic acid disruption in mice.

## Graphical abstract

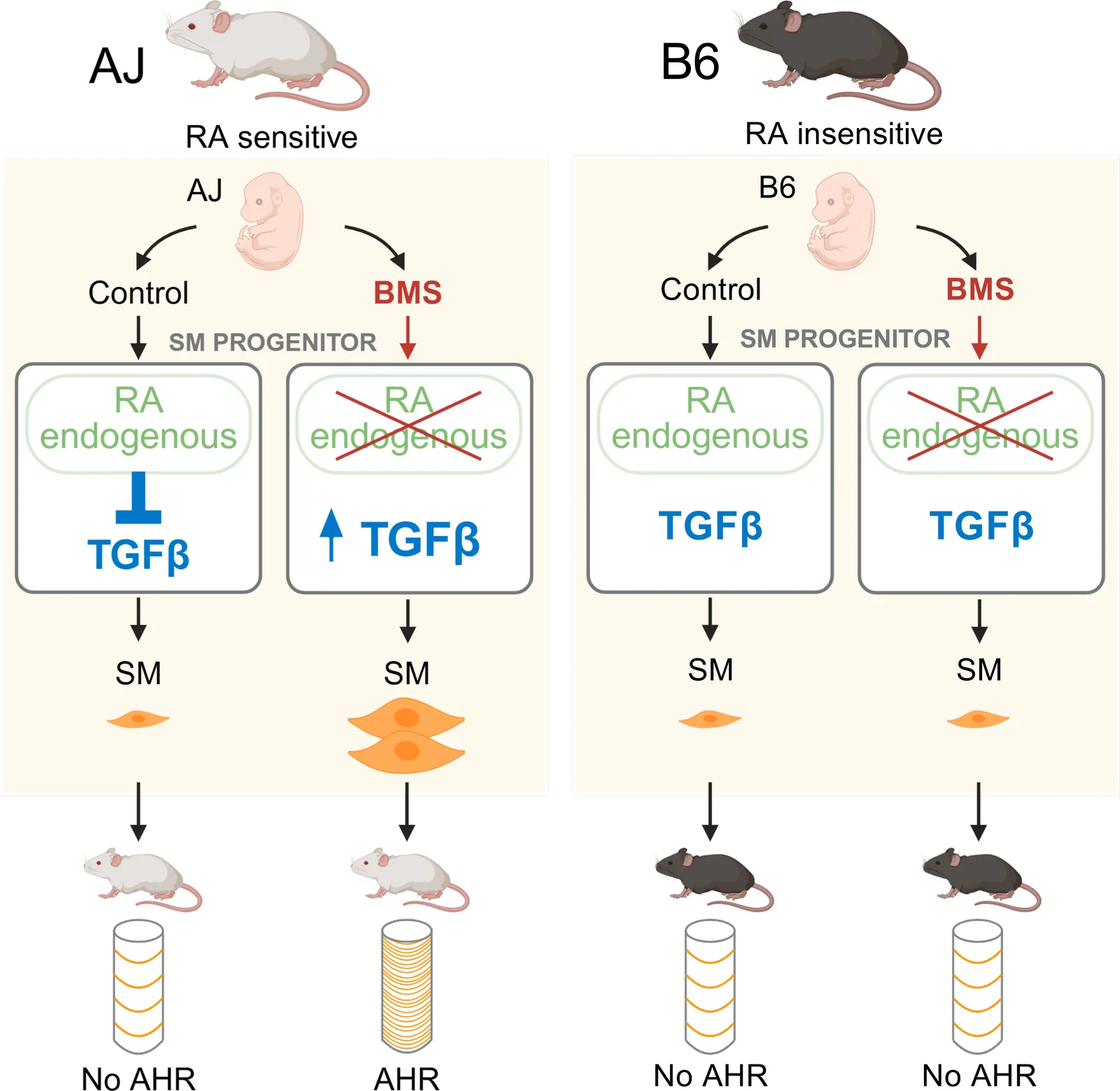

## Introduction

Genetic background in combination with environmental exposures can act as a risk factor for multiple human health conditions, influencing disease susceptibility and severity. One of these conditions is asthma, a disease characterized by phenotypes that include airway hyperresponsiveness (AHR), inflammation, and structural remodeling with narrowing of the distal airways. AHR is clinically defined as an increased airway sensitivity to non-specific stimuli or inhaled constrictor agonists, such as methacholine (MCh) (1). Although a hallmark of asthma, AHR is multifactorial and is seen as a manifestation of other pathological conditions often associated with airway structural changes, including an increase in airway smooth muscle (SM) mass (2). Mouse models have provided key insights into the genetic determinants of AHR susceptibility. Strain-dependent variations of AHR have been reported in different inbred mouse strains, and several AHR quantitative loci have been identified (3).

AHR variation is well illustrated from studies in C57BL/6J (B6) and A/J (AJ) mice, known for their distinct genetic backgrounds and use in pulmonary research. AJ mice are known for their susceptibility to developing AHR and airway remodeling, while B6 mice are more susceptible to developing emphysema (4). A transcriptome analysis of AJ and B6 lungs throughout the prenatal and postnatal stages reveals strain-related differences in gene expression signatures associated with a broad range of biological processes (5). This raises fundamental questions relevant to understanding the developmental origin of these abnormal airway responses. Are there differences in lung cellular behavior and composition that could ultimately reflect on distinct postnatal strain-related responses, such as AHR susceptibility and abnormal airway remodeling? How does the genetic background modulate the lung developmental programs associated with these responses?

An additional complexity to consider is the impact of maternal-fetal interactions influencing the developmental processes in a genetic background–dependent fashion. An essential component of these interactions is the establishment of an appropriate maternal micronutrient status in the intrauterine environment. Micronutrient perturbations have been linked to altered developmental events and postnatal adverse health outcomes (6). A large body of evidence implicates vitamin A and its active form, retinoic acid (RA), as key micronutrients regulating biological functions from early organogenesis to adulthood. In the adult lung, vitamin A/RA deficiency has been associated with airway SM dysfunction and AHR in animal models and humans (7, 8). Still, little is known about how genetic background influences these responses, likely contributing to the conflicting results on the relationship between vitamin A status and asthma risk (9). Compelling evidence of the lasting adverse effects of a brief prenatal vitamin A perturbation on adult lung function was previously reported. Mouse embryos briefly exposed to a vitamin A–deficient intrauterine environment develop subtle but relevant changes in airway SM structure and show AHR in adulthood (10).

The distinctive ability of AJ and B6 mice to undergo airway remodeling and AHR, and the observations reported above, provided an opportunity to explore this model of prenatal RA perturbation to address relevant questions about the developmental origins and the impact of genetic background in this phenotype. Are the lung developmental programs similarly sensitive to perturbations in the RA status in AJ and B6? If so, what makes them different, and what cellular and molecular changes are potential determinants of these strain-specific responses? We hypothesized that genetic background determines the susceptibility of the developing lung to transient disruption of RA signaling during a critical window of airway formation, such that the same prenatal perturbation would differentially affect developmental programs controlling airway SM differentiation and ultimately lead to strain-specific differences in adult AHR.

Here, we address these issues by performing comprehensive multiomics and functional analysis of AJ and B6 mice similarly subjected to prenatal RA deficiency. By disrupting RA signaling prenatally during a short developmental window, we found remarkably different responses in lung gene expression and function between these two strains. In contrast with B6, AJ mice showed an exacerbated response to RA disruption, activating TGF-β signaling and transcriptional targets differentially in a subpopulation of mesenchymal progenitor cells resulting in excessive ectopic SM formation in nascent airways. This phenotype was prevented when TGF-β signaling was inactivated in RA-deficient lungs from AJ embryos in utero. We show that this subpopulation is spatially restricted to sites where the SM program emerges in the distal developing lung, and that in AJ mice, is exquisitely sensitive to endogenous RA, which tightly controls activation of TGF-β and downstream targets required for proper SM differentiation. Collectively, these observations illustrate how distinct genetic backgrounds differentially influence developmental events and susceptibility to prenatal perturbations in the SM program that ultimately may result in strain-related differences in AHR in adulthood.

## Results

### Genetic background is a determinant of the aberrant response of the embryonic lung to an intrauterine RA-deficient environment and the susceptibility to AHR in adulthood.

**Figure 1:**
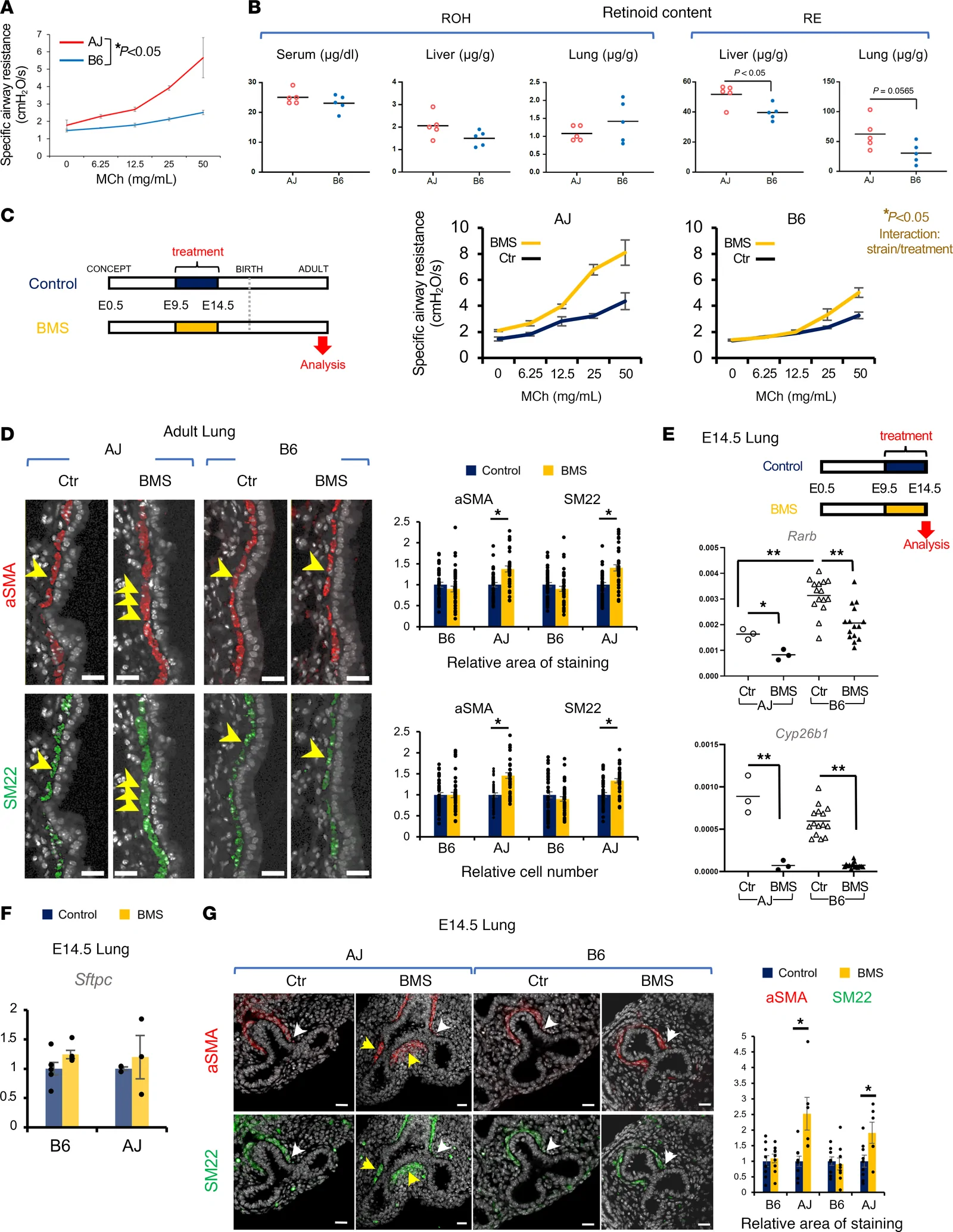
Genetic background determines airway smooth muscle (SM) program and airway hyperresponsiveness after transient prenatal RA deficiency. (**A**) Specific airway resistance (sRaw) in adult mice under methacholine (MCh) challenge. Significant differences in sRaw between AJ and B6 (*P* = 0.008, 2-way ANOVA). Mean ± SEM (*n* = 3 per group). (**B**) Retinoid concentrations (retinol [ROH], retinyl ester [RE]) in adult mice. *n* = 5 per group. Two-tailed Student’s *t* test. (**C**) Graphs: sRaw (mean ± SEM): AJ exhibited significantly higher BMS-induced airway resistance differences over Control than B6 as shown by 2-way ANOVA. AJ Control (*n* = 4), AJ BMS (*n* = 3), B6 Control (*n* = 9), B6 BMS (*n* = 6). (**D**) Immunofluorescence (IF) and quantitative analysis in adult mouse lungs exposed prenatally to BMS or control conditions. Two-tailed Student’s *t* test. AJ Control (*n* = 39), AJ BMS (*n* = 37), B6 Control (*n* = 45), B6 BMS (*n* = 43). Scale bars: 20 μm. (**E**) Expression of RA pathway components in E14.5 lungs (qPCR). Significant *Rarb* and *Cyp26b1* downregulation by BMS in both strains. One-way ANOVA followed by Tukey’s post hoc test. *P < 0.05, **P < 0.01. AJ Control (*n* = 3), AJ BMS (*n* = 3), B6 Control (*n* = 15), B6 BMS (*n* = 15). (**F**) qPCR of *Sftpc* in E14.5 lungs. Mean ± SEM. Two-tailed Student’s *t* test. AJ Control (*n* = 3), AJ BMS (*n* = 3), B6 Control (*n* = 6), B6 BMS (*n* = 5). (**G**) Analysis of E14.5 lungs. aSMA and SM22 IF and morphometric assessment of airway SM. Arrows: normal distribution (white) and ectopic accumulation (yellow) distribution. Graph: Mean ± SEM for each marker. *P < 0.05. Two-tailed Student’s *t* test. AJ Control (*n* = 9), AJ BMS (*n* = 6), B6 Control (*n* = 9), B6 BMS (*n* = 9). Scale bars: 20 μm.

A comprehensive analysis of the impact of genetic background on pulmonary function across 36 distinct inbred mouse strains showed remarkable differences in AHR in response to MCh (3). AHR can result from multiple factors, including abnormal inflammatory, structural, and neural responses. There is compelling evidence of an association between AHR and airway remodeling with prenatal disruption of vitamin A signaling in mice (10). We reasoned that further examining this association in mice with distinct genetic backgrounds could provide relevant insights into the developmental origins and prenatal events influencing AHR susceptibility. However, we had no evidence that the observations above, which were reported in an outbred (CD-1) line, could be reproduced in mice with defined genetic backgrounds. Even less certain was the relationship between vitamin A/RA status and AHR in mice of different backgrounds. Thus, to investigate these issues we selected AJ and B6, 2 widely used inbred mouse strains known for their markedly distinct responses to cholinergic agonist challenge (3). To confirm the suitability of these strains for our subsequent studies, adult 16-week-old male AJ and B6 mice were subjected to whole-body plethysmography (FinePoint), and specific airway resistance (sRaw) was measured under the nonchallenged state (baseline) or increasing concentrations of MCh (6.25, 12.5, 25, and 50 mg/mL). Airway resistance increased in both strains in response to increasing MCh concentration (Figure 1A). A 2-way ANOVA showed a significant main effect of MCh concentration (*P* = 0.016) and a significant main effect of strain, with AJ mice exhibiting significantly higher airway resistance than B6 mice (*P* = 0.008). Our findings of sRaw and AHR were consistent with airway resistance reported in the Mouse Phenome Database (AJ, 0.424 ± 0.096; B6, 0.321 ± 0.080; *P* < 0.05).

Given that differences in retinol (ROH) and retinyl ester (RE) levels have been reported in tissues from different adult inbred mouse strains, and that inadequate adult vitamin A/RA status has been linked to AHR, we asked whether the distinct sRaw/AHR of AJ and B6 reflected strain-dependent differences in retinoid bioavailability (11). The concentrations of ROH and RE in serum and in homogenates from lung and liver were measured in 8-week-old adult mice under a standard vitamin A–sufficient diet (Lab Diet, 5LG4). ROH levels in lung, liver, and serum were similar between strains (Figure 1B). Although AJ showed significantly higher RE levels in the liver (*P* < 0.05) and a trend toward higher RE levels in lung (*P* = 0.0565), overall, the differences in retinoid content between adult AJ and B6 mice were unlikely to explain the distinct AHR behavior between strains.

We then tested whether AJ and B6 embryos were equally susceptible to developing AHR in adulthood when briefly exposed to a vitamin A/RA deficient intrauterine environment during early lung development (10). To ensure effective and transient disruption of RA signaling in both strains, we used a more direct approach to prevent RAR activation in the embryo by orally administering mothers with a well-established pan-RAR reverse agonist, BMS493 (BMS) (10, 12). BMS administration could also minimize the variability in vitamin A uptake and retinoid bioavailability seen across strains. We conducted pilot studies to identify a BMS concentration that could simulate the effects of a mild-moderate vitamin A deficiency without the known teratogenic effects in embryonic growth or organogenesis, allowing offspring to survive to adulthood. Thus, AJ and B6 dams were daily gavaged with BMS at different concentrations (3.75–15 μg per body weight per day; μg/BW/day) or vehicle, corn oil (control group). Embryos were exposed to BMS in vivo from E9.5–E14.5, a developmental window encompassing the initial stages of branching morphogenesis and airway differentiation (10) (Supplemental Figure 1A; supplemental material available online with this article; https://doi.org/10.1172/jci.insight.200486DS1). Morphological analysis of E14.5 embryos exposed to the highest BMS concentration (15 μg/BW/day) showed severe effects in the body size and multiple developmental abnormalities, including the lung, most evident in AJ mice (Supplemental Figure 1, A and B). AJ lungs were severely hypoplastic compared with respective controls and B6 lungs. These abnormalities were largely undetected at 3.75 μg/BW/day. Lungs from both AJ and B6 embryos exposed to BMS at this concentration appeared macroscopically comparable to respective controls (Supplemental Figure 1, A and B).

qPCR analysis of these embryonic lungs confirmed that intrauterine exposure to BMS 3.75 μg/BW/day effectively disrupted RA signaling, as seen by the significant decrease in *Rarb* and *Cyp26b1* expression compared with controls (*n* ≥ 3, *P* < 0.05). Importantly, *Sftpc*, which marks the epithelium of growing distal buds, showed no significant differences in expression between control and BMS lungs in both strains (Figure 1, E and F). This ensured that BMS was effective in disrupting RA signaling but not lung epithelial growth and branching. To further demonstrate that the prenatal perturbation in RA signaling did not result in the teratogenic effects that prevent viability, we monitored these mice until adulthood. Embryos from both AJ and B6 mothers exposed to BMS (3.75 μg/BW/day; E9.5–E14.5) were viable, comparable with controls, and reached adulthood. No difference in BW or liver weight relative to BW was found in mice prenatally exposed to BMS or control in both strains. Although we detected a small difference in lung weight relative to BW between adult mice prenatally exposed to BMS compared with control conditions, this difference was similarly found in both AJ and B6 (*n* > 3, from at least 2 dams) and, importantly, did not prevent animals to undergo postnatal development and reach adulthood as in controls (Supplemental Figure 1, C and D).

Next, we asked whether the brief disruption of RA signaling in embryos exposed to BMS differentially influenced the susceptibility of AJ or B6 mice to develop AHR in adult life. sRaw was assessed at baseline and after MCh challenge in adult 16-week-old male mice from all groups as before. Brief intrauterine exposure to an RA-deficient environment consistently resulted in aberrant higher sRaw at baseline and across all MCh concentrations in adult AJ, but not B6, which responded to the challenge only at the highest MCh concentration (Figure 1C). Two-way ANOVA revealed that both strain and BMS treatment contributed to explaining the variance in AHR at the increasing MCh concentrations tested. Crucially, it also showed that AJ-specific BMS-induced airway resistance over control was significantly higher than that found in B6 (strain-treatment interaction *P* value: 0.018).

We reasoned that the hyperresponsiveness of BMS-AJ was associated with some structural changes in the airway SM, as suspected by similar observations previously reported in CD-1 mice. Indeed, immunofluorescence (IF) and quantitative analysis confirmed the increased thickness in airway SM labelled by α-smooth muscle actin (aSMA) and SM22 in the adult AJ lungs compared with B6 (Figure 1D). To confirm the developmental origin of this phenotype, we examined embryonic lungs from both AJ and B6 mice immediately after the last day of exposure to maternal BMS administration. E14.5 BMS-AJ lungs showed no obvious abnormalities in epithelial patterning or growth; however, aSMA and SM22 IF revealed increased ectopic accumulation of airway SM, consistent with the pattern previously reported in vitamin A–deficient embryos in CD-1 mice (10). By contrast, disruption of RA signaling had no effect on SM markers in BMS lungs from B6 embryos (Figure 1G).

These findings were consistent with the idea that differences in genetic background are major determinants of how the lung responds to prenatal perturbations in RA signaling and the potential link to the susceptibility to AHR in adult life.

### AJ and B6 lungs differ broadly in gene expression signatures, with minimal differences in cellular composition in response to prenatal RA deficiency.

**Figure 2:**
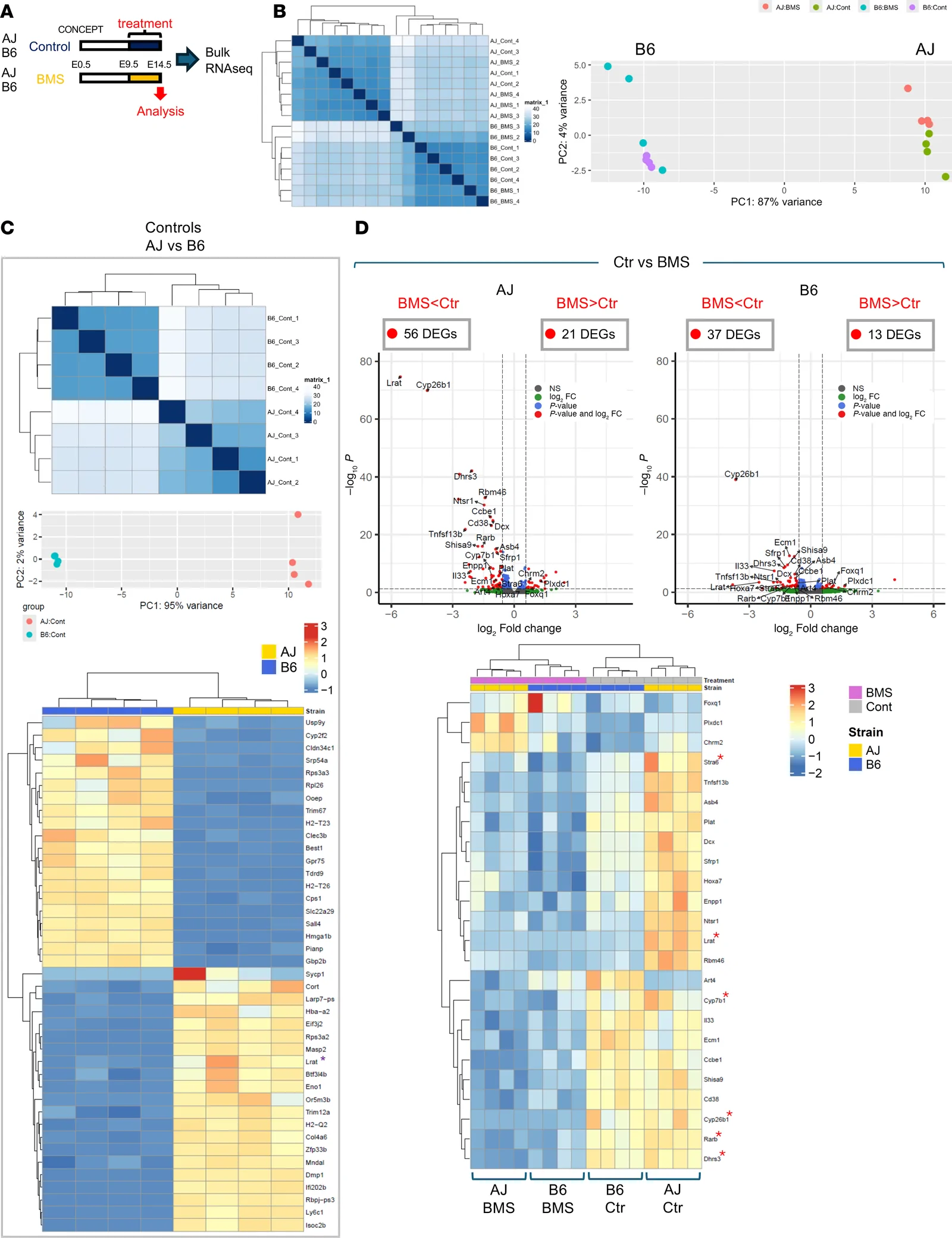
AJ and B6 developing lungs have distinct gene expression signatures under RA deficiency. (**A**) Experimental design. (**B**) Heatmap of sample-to-sample distances: segregation by strain. PCA plot shows PC1 (strain effect) and PC2 (BMS effect). (**C**) Differential gene expression between controls. Unsupervised heatmap depicting the top genes enriched in each strain (*Lrat enriched in AJ). (**D**) Volcano plots: DEGs in BMS versus control embryonic lungs. Number of DEGs depicted in boxes (*P*adj < 0.05 and |log_2_[fold change]| > 0.58). Supervised heatmap, highlighting BMS-downregulated markers of RA activity (*).

The distinct response of AJ and B6 embryos to prenatal disruption of RA signaling led us to examine differences in lung gene expression signatures associated with these distinct backgrounds. First, we performed bulk RNA-seq in E14.5 lungs from AJ and B6 embryos intrauterine exposed to BMS or control conditions (*n* = 4 per group). Interestingly, principal component analysis (PCA) showed that the strain-related differences in gene expression between control AJ and B6 lungs were greater than those resulting from the prenatal exposure to BMS in each strain (87% variance vs. 4% variance) (Figure 2, A and B, and Supplemental Table 1). Indeed, control AJ and B6 lungs differed greatly in their signatures, as revealed by 1258 differentially expressed genes (DEGs) (Figure 2C and Supplemental Figure 2B).

We searched for putative direct RA targets among these genes, initially by looking for RA-responsive element–containing (RARE-containing) open chromatin regions (RARE-OCRs) in publicly available ENCODE ATAC-seq mouse datasets from E14.5–E16.5 lungs, representative of early stages of lung differentiation (5) (Supplemental Figure 2A). This revealed 28,355 RARE-OCRs with an irreproducible discovery rate of less than 0.05; nearly half of these (44.2%) were identified within the promoter region of genes, potential RA targets (RARE-OCR genes, Supplemental Table 2). The data suggested a robust RA-responsive gene network at these developmental stages. The proportion of RARE-OCRs that overlapped with genes differentially expressed between control AJ and B6 lungs was not markedly different (AJ: 39.7%; B6: 54.4%). However, gene set enrichment analysis (GSEA) suggested that they differed considerably in downstream pathways and presumably function. For example, analysis of RARE-OCR–containing genes enriched in AJ included Gene Ontology (GO) terms, such as growth factor activity, lyase activity, and transferase activity, while those enriched in B6 included ECM-related pathways and growth factor binding (Supplemental Figure 2B). Moreover, confirming our initial findings, *Stra6* and *Lrat*, key genes associated with vitamin A uptake and retinoid bioprocessing/storage, were among the DEGs upregulated in control AJ compared with B6 (Supplemental Figure 2D and Supplemental Table 3) (13, 14).

We then compared the impact of maternal BMS exposure on gene expression signatures of AJ and B6 embryonic lungs. Efficient disruption of RA signaling was confirmed in both strains by downregulation of RA pathway target genes (*Rarb*, *Cyp26b1*, and *Dhrs3*) as well as other known RARE-OCR–containing genes such as *Hox* family members (Figure 2D, Supplemental Figure 2, C and E, and Supplemental Table 4). GSEA of DEGs in BMS versus control lungs from AJ embryos revealed significant enrichment for terms associated with the suppression of RA-related molecular function (e.g., RA binding), as well as enrichment for terms associated with activation of muscle contraction and microfilament activity, none of which were found in B6 lungs (Supplemental Figure 2F and Supplemental Figure 3A). Consistent with this, analysis of transcription factor activity using the R package *decoupleR* with the univariate linear model (ULM) approach showed decreased *Rarg*, *Rarb*, and *Rora* activity and increased activity of transcription factors, such as *Egr1*, *Id2*, *Med1*, *Tbx2*, and *Tbx3* associated with muscle development (GO: 0060537) in AJ BMS (Supplemental Figure 3B). This was not observed in BMS versus control B6 lungs, suggesting a less robust RA-related functional activity in these events. Together, the data pointed to broad differences in the gene expression signatures of the developing AJ and B6 lungs, including regulators of retinoid bioavailability and RA targets. However, these differences could hardly justify their role as determinants of the altered response of AJ mice to an RA-deficient environment.

To further investigate whether the distinct behavior of AJ and B6 resulted from predominant changes in gene expression in a specific cell population in response to BMS, we performed single-nucleus multiome analysis (snRNA-seq and snATAC-seq) of these lungs exposed to BMS under the same conditions. After filtering low-quality entries, 21,293 nuclei were examined across all conditions (AJ: control = 4750 and BMS = 6363; B6: control = 5321 and BMS = 4859). Using weighted nearest neighbors (WNN) to integrate snRNA-seq and snATAC-seq data, 4 populations were identified broadly as epithelial, mesenchymal, endothelial, and immune cell based on reported markers and chromatin structure profiles (Supplemental Figure 4A and Supplemental Figure 5A) (15). No significant strain-specific differences in cell proportion were found in any of these 4 cell populations in response to prenatal disruption of RA signaling (Supplemental Figure 4B). This was somewhat unexpected since the genetic background can account for subtle but often important variations in cellular composition, resulting in strain-dependent differences in organ size and patterns of growth (16). By contrast, we found major changes in gene expression and to a lesser extent open chromatin accessibility in response to BMS in both strains. Consistent with our bulk RNA-seq findings, BMS exposure resulted in a higher number of DEGs in AJ lungs (*n* = 548) compared with B6 (*n* = 214). Moreover, BMS-AJ also had a higher number of differentially accessible regions (DARs) (*n* = 117) than BMS-B6 (*n* = 71); of these, 61 out of 117 (52%) and 37 out of 71 (52%), respectively, harbored RARE-OCRs (Supplemental Figure 5B and Supplemental Tables 5 and 6). The lower number of DARs compared with DEGs suggested that differences in transcription factor expression were more likely to be the primary driver of the RA-mediated events rather than shifts in the epigenetic state. An initial analysis of the top DEGs in each of these populations was unremarkable. The collectively higher number of DEGs and DARs in the mesenchymal cell population of AJ compared with B6 and the AJ-distinctive SM differentiation program in response to BMS, led us to further focus our analysis on the lung mesenchyme.

### Distinct subpopulations emerge from the mesenchymal cell compartment during airway morphogenesis in both AJ and B6 lungs.

**Figure 3:**
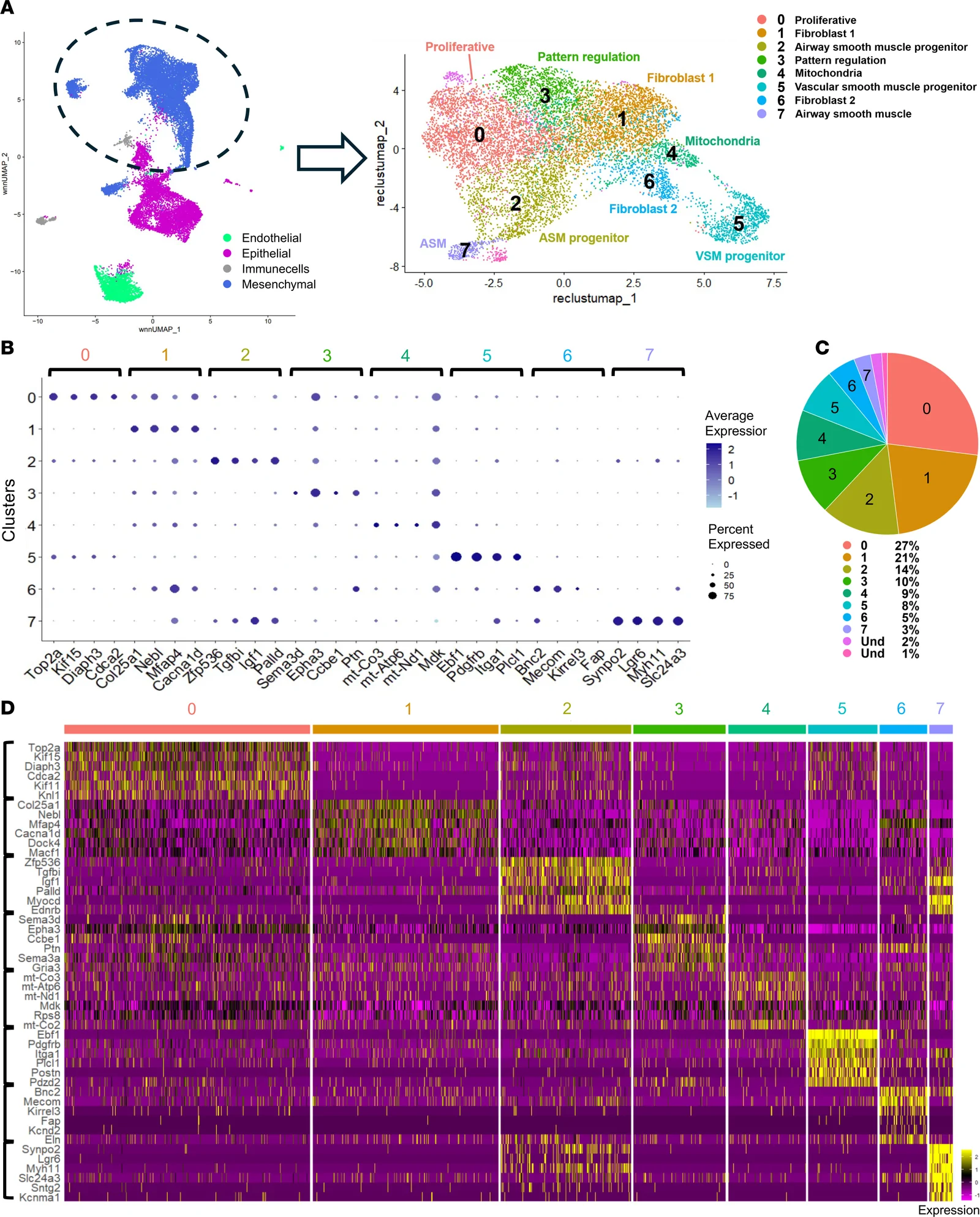
Single-nucleus multiomics identifies multiple subpopulations of lung mesenchymal cells. (**A**) UMAP projection of reclustered mesenchymal cells (all conditions and strains). Eight subpopulations identified by established markers. (**B**) Dot plot of top differentially enriched genes for each of the mesenchymal cluster. (**C**) Pie chart depicting the relative proportion of each mesenchymal cell subpopulation. (**D**) Heatmap of top differentially enriched genes for each of the mesenchymal cluster.

**Figure 4:**
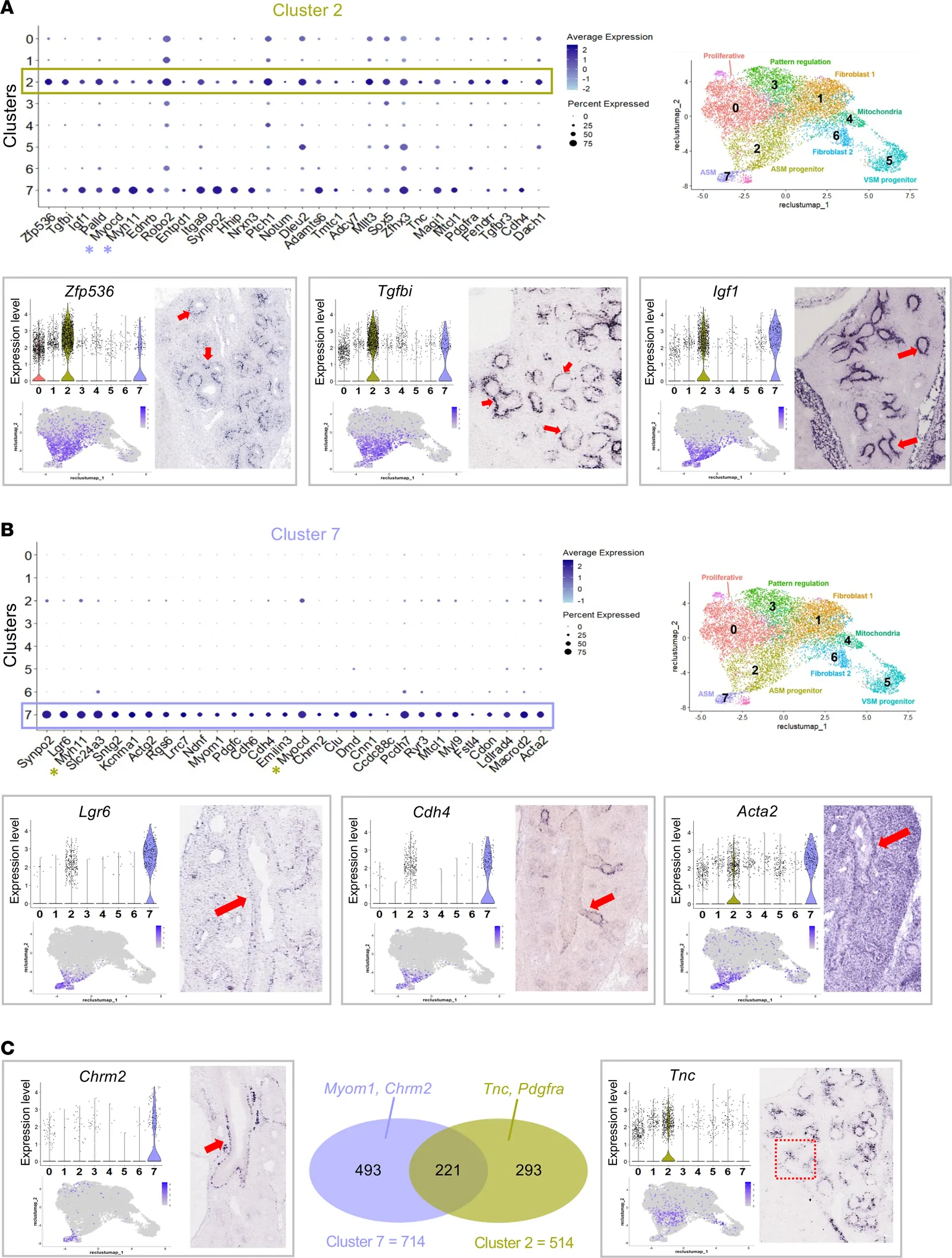
Multiomics analysis identifies 2 distinct stages of airway SM cell program during lung morphogenesis. (**A** and **B**) Clusters 2 and 7: Dot plots displaying top DEGs (*P*adj < 0.05). Representative genes from each cluster are depicted in violin and feature plots. In situ hybridization (GenePaint; http://www.genepaint.org/): Distinct spatial distribution of these populations in distal mesenchyme where the SM emerges (Cluster 2) or in already established airway SM at E14.5 (Cluster 7). (**C**) Violin plots and Venn diagram depicting unique and shared genes between Clusters 2 and 7. Red arrows and dashed box depict gene expression in airway smooth muscle.

Given the higher number of DEGs and DARs in the mesenchymal compartment of BMS-AJ compared with BMS-B6 (Supplemental Figure 5B), the known diversity of lung mesenchymal cell types, and the dynamic morphogenetic changes taking place in the E14.5 lung, we performed a comprehensive analysis of this compartment to understand how BMS influenced its gene expression and behavior. First, we computationally filtered for the mesenchymal cells from our multiome datasets, combining all groups. Leiden clustering identified 8 subpopulations, annotated based on established markers (Seurat *FindMarkers* with the Wilcoxon rank-sum test) and 2 other small indistinct subpopulations with mixed signatures, not further analyzed (Figure 3, A–D, and Supplemental Table 7). Cluster 0 was identified as undifferentiated proliferative cells for their enrichment in cell cycle–related genes (*Top2a*, *Kif15*, and *Mki67*) (Supplemental Figure 6A). Clusters 1 and 6 consisted of 2 populations of fibroblast-like cells based on their expression of ECM-related genes (Fibroblast 1: *Col25a1*, *Mfap4* and Fibroblast 2: *Bnc2*, *Fap*) (Supplemental Figure 6C). Cluster 4 was annotated as “mitochondria-enriched mesenchyme” (*mt-Co3*, *mt-Atp6*, and *mt-Nd1)* (Supplemental Figure 6B). Cluster 3 was characterized by enrichment in genes associated with biological processes, such as cell migration, morphogenetic movements, and chemoattraction reported mostly in neuronal studies (*Sema3d*, *Epha3*, and *Ptn*), but also featuring key regulators of lung pattern formation and mesenchymal differentiation (*Wnt2*, *Fgf10*, *Rspo2*, and *Ctnna2*) (Supplemental Figure 7A). Two populations (Clusters 5 and 7) have common expression statuses of genes associated with the SM program (Supplemental Figure 6E). However, Cluster 5 was annotated as “vascular smooth muscle” (pericytes) based on the differential enrichment in *Pdgfrb*, *Heyl*, and *Notch3*, compared with Cluster 7 “airway smooth muscle” identified by markers such as *Lgr6*, *Actg2*, *Cdh4*, *Acta2*, *Myom1*, *Chrm2*, and others (Figure 4B and Supplemental Figure 6, D and E). Lastly, Cluster 2 was enigmatic as it encompassed a population of undifferentiated mesenchymal progenitors marked by *Zfp536*, *Tgfbi*, and *Igf1*, but also expressing early stages of the SM cell program (*Myocd* and *Myh11*), overall distinct from Clusters 5 and 7 (Figure 4A).

A systematic survey of the spatial localization of top markers from each cluster showed partially overlapping but also distinct expression patterns in the E14.5 developing lung. Transcripts from Cluster 0 markers were found throughout the lung mesenchymal compartment, consistent with the broad distribution of proliferative cells at this stage (Supplemental Figure 6A). This contrasted with the more spatially restricted distribution of Cluster 2, 3, 5, and 7 genes (Figure 4, A and B, Supplemental Figure 6, D and E, and Supplemental Figure 7, A and C). Notably, analysis of Cluster 2 markers revealed a remarkable pattern of transcript distribution in the mesenchyme at the stalk region of distal buds from where the SM program emerges (Figure 4A and Supplemental Figure 8A). Based on its signature, gene ontology, and spatial distribution, Cluster 2 was annotated as “airway smooth muscle progenitor,” reflecting its enrichment in genes associated with initiation of the cell fate program toward this lineage. Indeed, evidence from multiple studies, as well as our GSEA/GO results, showed Cluster 2 top differentially enriched genes, components of the TGF-β, IGF, and PDGF pathways (*Tgfbi*, *Pdgfra*, and *Igf1*) reported as early regulators of the SM program (17–19). Also enriched in Cluster 2 we found *Ptch1* and *Hhip*, components of the Shh pathway reported as crucial for initiation of the airway SM program (20). By contrast, Cluster 7 cells were most distinctly identified by the expression of more definitive markers of SM differentiation and for their localization to proximal airways (Figure 4, B and C, and Supplemental Figure 8B). The analysis also underscored the closer relationship in the signature of Clusters 7 and 2 compared with the others. For example, among the 714 and the 514 genes identified as markers of Clusters 7 and 2, respectively, 30% or more genes were common to both clusters (221 genes; *P*adj < 0.05, absolute log_2_[fold change] > 0.585) (Figure 4C and Supplemental Table 14). These overall (e.g., *Myocd* and *Myh11*) differed in levels or percentages of cells expressed in Clusters 7 or 2 and likely include cells undergoing a transition from commitment to initiation of the SM program in the highly dynamic niche of the E14.5 distal lung (dot plots in Figure 4, A and B). By contrast, Clusters 7 and 5, in spite of being identified as SM (airway and vascular, respectively), shared only approximately 10% of their DEG signatures (Supplemental Figure 6E). Notably, DEGs in Cluster 3 overall showed a characteristic spatial transcript distribution in the distal mesenchyme, often in a subpleural location and found collectively by GO to be regulators of cell migration, morphogenetic movements, and chemoattraction, widely reported in neurons and the lung. Additional Cluster 3 enriched genes, such as *Wnt2* and *Fgf10*, are known for their prominent role in lung pattern formation and shown by lineage tracing analysis to label early progenitors of the airway SM program (Supplemental Figure 7, A and B) (21). Moreover, *Wnt2* has been shown to regulate the airway SM program through MRTFB/myocadin and FGF10 (22).

Altogether, from these annotations we propose a developmental model that aligns well with that proposed by Kumar et al. (21), which describes a progression from undifferentiated tip bud mesenchymal progenitors (Cluster 3) to recruitment to the airway SM lineage around the stalk region of distal lung buds (Cluster 2) to ultimately differentiate into mature SM cells (Cluster 7) (Supplemental Figure 7D).

### Endogenous RA signaling is differentially required in a specific TGF-β–activating mesenchymal progenitor population to prevent aberrant SM program of AJ lungs during morphogenesis.

Having identified the signature characteristic of these mesenchymal clusters and their spatial distribution in the E14.5 lung, we then asked what differences in cellular composition and gene expression could be attributed solely to the genetic background of each strain, selectively in the mesenchymal cell compartment. For this, we compared the gene expression signature of E14.5 AJ and B6 under unperturbed (control) conditions. Transcriptome analysis showed that AJ and B6 control lungs differed significantly in the number of DEGs in Cluster 0 (478 DEGs), Cluster 2 (273 DEGs), and Cluster 3 (161 DEGs), with fewer differences in the remaining mesenchymal clusters (Supplemental Figure 9A and Supplemental Table 10). Although interesting, we could not rule out that this could be in part due to an overall higher number of cells in these clusters. Notably, we had no evidence that endogenous RA signaling was preferentially more active in the mesenchymal populations of control AJ compared with B6 lungs. *Rarb*, a surrogate marker of RA activation, was similarly expressed in all clusters of controls from both strains (Supplemental Figure 9B). A comparison of the gene expression signature of Clusters 0, 2, and 3 from control AJ and B6 lungs revealed *Mgp*, *Negr1*, *Col1a1*, and *Opcml* among the top DEGs significantly downregulated in AJ in these 3 clusters. This signature was also found in AJ controls when all mesenchymal clusters were examined collectively (Clusters 0–9) and when AJ and B6 were analyzed by bulk RNA-seq of whole-lung homogenates. These results strongly suggest that these genes represent key components of the signatures that distinguish AJ from B6 in E14.5 lungs, ultimately associated with the strain differences in genetic background (Supplemental Figure 9, C–F and Supplemental Table 12).

**Figure 5:**
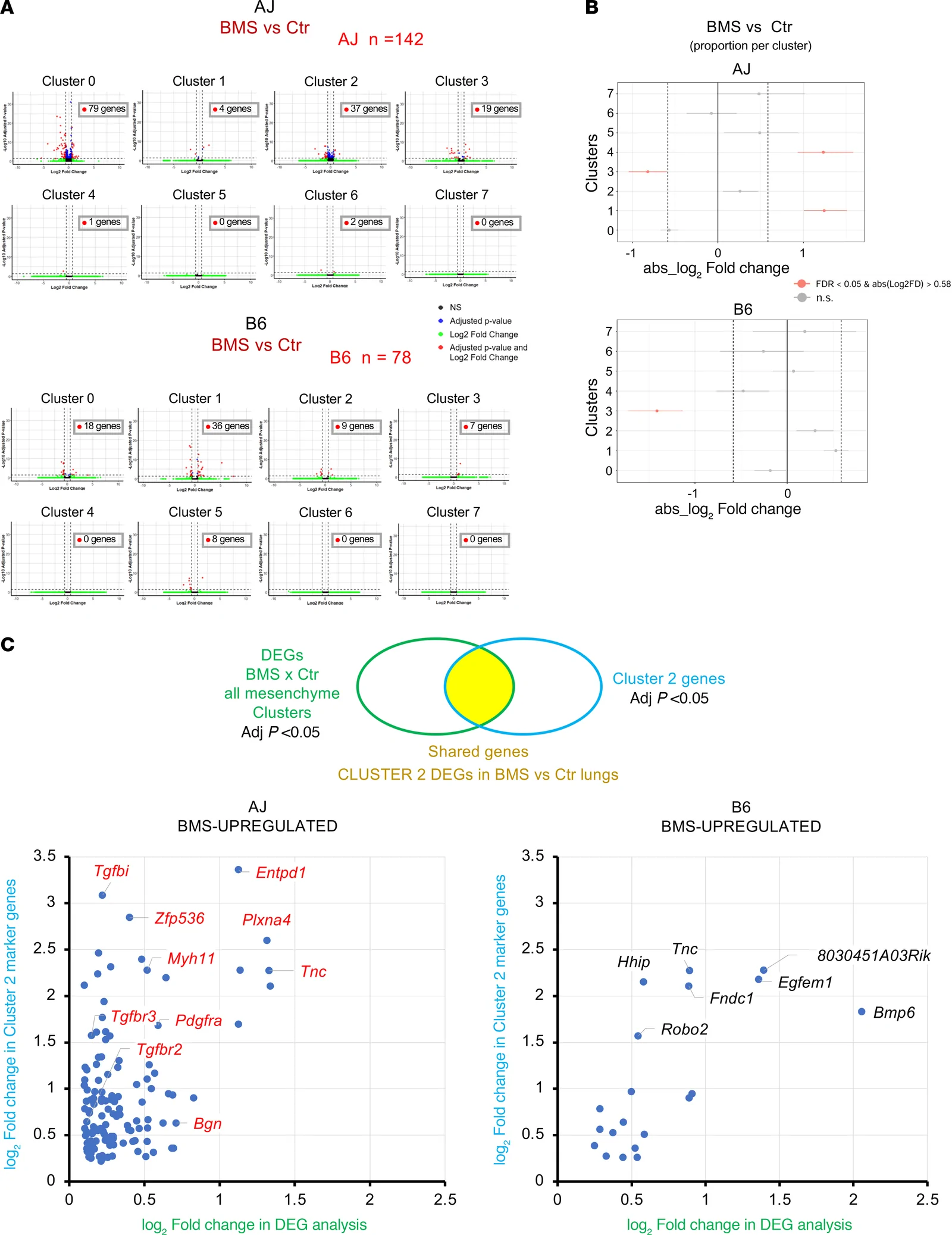
Cluster 2 cells are enriched in regulators of SM program and respond differentially to an RA-deficient intrauterine environment in AJ and B6 lungs. (**A**) Single-nucleus multiomics analysis of BMS versus control lungs. Volcano plots: Higher number of DEGs in AJ, particularly in Clusters 0, 2, and 3. Boxes depict DEG number with |log_2_(fold change)| > 0.58 and adjusted *P* < 0.05. (**B**) Graph depicting changes in cell proportion in response to BMS for each mesenchymal cluster. Differences between AJ and B6 are significant if FDR < 0.05, |log_2_(fold change)| > 0.58. Permutation testing (*n* = 10,000). (**C**) Identification of mesenchymal Cluster 2–enriched genes among the DEGs upregulated by BMS in AJ lungs. Graphs represent the |log_2_(fold change)| in expression between BMS and control conditions across all mesenchymal clusters from whole mesenchymal analysis (*x*-axis) against the |log_2_(fold change)| of genes selectively enriched in Cluster 2 (*y*-axis).

Next, we assessed the impact of intrauterine exposure to BMS on the gene expression signature of AJ and B6 developing lungs. Single-nucleus multiomics showed a significantly higher impact of BMS on the transcriptomics of AJ lungs. Altogether, the number of DEGs between control and BMS in all clusters was almost twice as high in AJ compared with B6 lungs (*n* = 142 and *n* = 78, respectively), with the majority of these found in Clusters 0, 2, and 3, regardless of differences in cell proportions (Figure 5, A and B, and Supplemental Tables 8 and 9). We leveraged the statistical power of a global analysis collectively of all mesenchymal clusters from our AJ and B6 single-nucleus transcriptome dataset to identify DEGs between control and BMS-exposed lungs. Although mesenchymal disruption of RA signaling was confirmed by downregulation of *Rarb* in both strains, strain-specific differences in gene expression in response to BMS were clearly detected between AJ and B6. These included downregulation of additional components of the RA pathway (*Lrat* and *Dhrs3*), accompanied by upregulation of TGF-β targets and known regulators and markers of SM program in the mesenchymal population of BMS-AJ lungs (Supplemental Figure 10, A and B, and Supplemental Table 11).

Given our goal to identify potential regulators of the strain-specific responses of the mesenchymal cells to an RA-deficient environment, we established the following more stringent criteria for further analyses: (a) we examined the DEGs unique to AJ not differentially altered by BMS in B6 lungs; (b) among these, we focused on the genes upregulated by BMS in AJ consistent with our original hypothesis that endogenous RA prevents an unrestrained aberrant program of SM progenitors during lung morphogenesis; and (c) using information gathered from our cluster analysis and expression pattern, we selected DEGs whose mRNA distribution mapped to putative sites of initiation of the SM program in the E14.5 distal lungs. Based on these criteria, Cluster 2 emerged as the cell population to be the prime candidate for analysis. We first asked which of the DEGs identified by analysis of control versus BMS collectively in all mesenchymal cells featured among the markers of Cluster 2 cells. We then identified the Cluster 2 genes differentially upregulated by BMS in AJ, but not in B6. This revealed *Tgfbi*, *Pdgfra*, *Entpd1*, and *Zfp536* and other top markers of Cluster *2* (Figure 5C). The identification of widely reported regulators of the SM program among these genes supported the criteria for further screening (17, 18, 23, 24). Since BMS did not significantly alter the proportion of Cluster 2 cells in AJ or B6 lungs (Figure 5B), we concluded that the upregulation of the Cluster 2 genes found in AJ likely resulted from their derepression in response to the disruption of RA signaling selectively in this strain. Cluster 2 genes downregulated by BMS are shown in Supplemental Figure 11B.

A large number of studies confirm the essential role of TGF-β signaling in SM development and in remodeling of the injured lung in diseases such as pulmonary fibrosis and asthma (25). The regulation of TGF-β signaling by RA in airway SM differentiation has also been extensively reported both in the developing and the adult lung. Disruption of RA signaling leads to hyperactivity of the TGF-β pathway at the onset of lung development and later during branching morphogenesis (12, 26). Here, we found key components of the TGF-β pathway enriched in the mesenchymal compartment of the E14.5 lungs, with *Tgfbi* and *Tgfbr3* appearing as top markers of Cluster 2 (Supplemental Figure 13). *Tgfbr3* is a co-receptor crucial for activation of TGF-β/SMAD signaling through TGFB2-TGFBR2 binding and phosphorylation of TGFBR1 (27). *Tgfbi* is a well-established early target gene of the TGF-β pathway (12).

**Figure 6:**
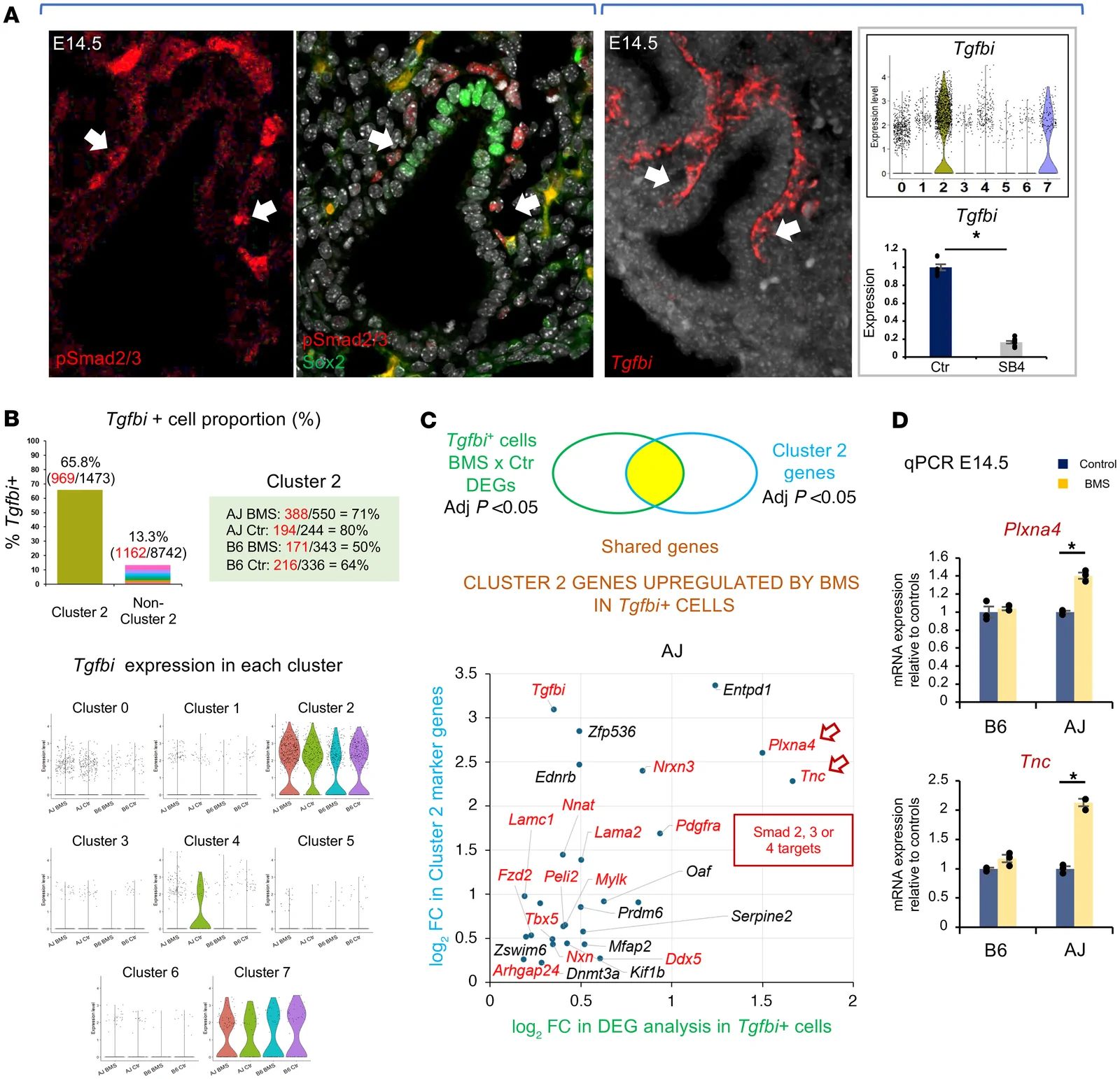
TGF-β–activating cells are key determinants of the distinct response of AJ to prenatal disruption of RA signaling. (**A**) Image panels: *Tgfbi* transcript distribution in distal E14.5 mesenchyme, and p-SMAD2/3 IF signals in the stalk mesenchyme associated with the emergence of Sox2^+^ airway epithelial progenitors. Violin plot: *Tgfbi* enrichment in Clusters 2 and 7. White arrows depict pSMAD2/3+ (left and middle panels) and *Tgfbi*+ regions (right panel). Graph: *Tgfbi* expression in lung explants cultured under control or SB431542-containing (SB4-containing) media. Marked downregulation by disruption of endogenous TGF-β signaling. qPCR: Mean ± SEM, 2-tailed Student’s *t* test, Ctr (*n* = 7), SB4 (*n* = 8). (**B**) Quantitative analysis of the *Tgfbi*^+^ cell proportion in Cluster 2 compared to all other clusters and effect of BMS. Highest *Tgfbi* expression in Clusters 2 and 7. (**C**) Top: Venn diagram depicting Cluster 2–enriched genes among the DEGs upregulated by BMS in *Tgfbi*^+^ cells of AJ lungs (*P*adj < 0.05 and |log_2_[fold change]| > 0). Bottom: Differences in gene expression in BMS versus control *Tgfbi*^+^ cells (|log_2_[fold change]|; *x*-axis) against the |log_2_(fold change)| of genes selectively enriched in Cluster 2 (*y*-axis). Genes carrying SMAD2, SMAD3, or SMAD4 binding sites (marked in red) found upregulated by BMS in *Tgfbi*^+^ cells from AJ lungs. (**D**) qPCR analysis of lung homogenates from E14.5 embryos. Significant upregulation by BMS only in AJ (2-tailed Student’s *t* test, *n* = 3 per group). *P < 0.05. All images: ×20 magnification.

Interestingly, analysis of E14.5 lungs revealed expression of *Tgfbr3*, *Tgbi*, and p-SMAD2 and -3 signals converging at the stalk mesenchyme where airway progenitors (*Sox2*^+^) emerge and the SM program initiates (Figure 6A and Supplemental Figure 13). This led us to hypothesize that the TGF-β pathway could be aberrantly active locally, altering key developmental events in response to disruption of endogenous RA signaling. We started investigating this possibility by comparing the SMAD2/3 phosphorylated status locally in the distal lung undergoing morphogenesis ex vivo under control and RA-deficient conditions.

Embryonic lungs from AJ and B6 mice were cultured under well-established conditions in control or BMS-containing media and analyzed after 48 hours (12). Efficient disruption of RA signaling was confirmed by downregulation of RA targets (*Rarb* and *Cyp26b1*) in both AJ and B6 lung cultures. IF of aSMA confirmed ectopic induction of airway SM in AJ, but not in B6-derived lung cultures, confirming the strain-dependent differential response we observed in vivo (Supplemental Figure 12, A and B). Control cultures showed the typical distribution of p-SMAD2/3 signals in the distal mesenchyme associated with lung bud stalks also observed in vivo (Supplemental Figure 12C and Figure 6A). By contrast, we found p-SMAD2/3 signals markedly increased and sharper in the distal mesenchyme of BMS-treated AJ lung cultures. No difference in p-SMAD2/3 staining was observed between control and BMS-treated lung cultures from B6 mice (Supplemental Figure 12D). The data strongly supported the idea of a strain-dependent regulation of TGF-β activation by RA in a subpopulation of progenitor cells undergoing SM fate specification in developing airways. The overactive TGF-β signaling was evident by the increased p-SMAD2/3 signals and upregulation of *Tnc*, *Pdgfra*, and *Plxna4*, known targets of TGF-β signaling, in BMS-treated AJ cultures compared with controls (Supplemental Figure 12E).

There is accumulated evidence that *Tgfbi* identifies sites of canonical TGF-β activation (28, 29). In the E14.5 lung, we found *Tgfbi* transcript distribution was closely associated with sites of SMAD2/3 phosphorylation in the distal mesenchyme, and overall levels were markedly downregulated when endogenous TGF-β signaling was inhibited by SB431542 in lung embryonic explant cultures (Figure 6A). Given the evidence of the differential upregulation of TGF-β pathway genes by BMS in AJ lungs, we raised the possibility that *Tgfbi*^+^ cells may harbor key mediators of this differential response. Although largely represented in the Cluster 2 population, *Tgfbi* was also found to a lesser extent in other clusters (66% of 1473 Cluster 2 cells, 13% of 8742 non–Cluster 2 cells) (Figure 6, A and B, and Supplemental Figure 14A). Thus, we computationally filtered all *Tgfbi*^+^ cells and collectively investigated their gene expression signature in each experimental condition. *Tgfbi*^+^ cells were filtered by selecting cells with normalized *Tgfbi* expression greater than 0.1. Analysis of control lungs from both strains showed a relatively high proportion of *Tgfbi*^+^ cells in AJ (27%) compared with B6 (16%). This proportion was largely unchanged by BMS when compared to controls from respective strain (AJ BMS: 25%; B6 BMS: 14%). By contrast, the number of DEGs in BMS versus control *Tgfbi*^+^ cells was remarkably different in AJ (*n* = 337) compared with B6 lungs (*n* = 6) (*P*adj < 0.05) (Supplemental Figure 11A and Supplemental Table 13). Notably, while we found 79 DEGs upregulated by BMS in *Tgfbi*^+^ cells from AJ, only 3 DEGs were upregulated in BMS-B6 lungs (Supplemental Figure 14B). Given that the proportion of *Tgfbi*^+^ cells was unchanged by BMS in both strains, we conclude that disruption of RA signaling resulted in overly activated TGF-β signaling selectively in this cell population of AJ lungs. In searching for a potential reason for this discrepancy, we examined a panel of markers of RA activation in *Tgfbi*^+^ cells from both strains. Surprisingly, although *Rarb* and *Cyp7b1* were consistently expressed in *Tgfbi*^+^ cells from controls in both AJ and B6 lungs, these genes were downregulated by BMS only in AJ lungs (Supplemental Figure 14C). This suggested that the *Tgfbi*^+^ cell population of B6 was selectively insensitive to BMS and maintained its normal program, while the same population in AJ lungs under the same conditions responded aberrantly derepressing genes whose levels would be normally controlled by endogenous RA in these cells. Their identification as TGF-β–activating cells in both strains and their distinct response to an RA-deficient environment are consistent with the idea that the *Tgfbi*^+^ cells represented the key cellular target of the SM phenotype observed in BMS AJ lungs.

To further refine our analysis and enrich genes with a greater chance of being mediators of the BMS aberrant phenotype, we first identified DEGs from cluster 2 that were most upregulated by BMS in AJ *Tgfbi*^+^ cells (*P*adj < 0.05 and absolute log_2_[fold change] > 0). This ensured a greater representation in genes predicted by GSEA/GO analysis of Cluster 2 to be regulators of the SM program. Second, given that *Tgfbi*^+^ cells were identified for their differential activation of TGF-β signaling, we used the public dataset (ChEA Transcription Factor Targets 2022; https://maayanlab.cloud/Harmonizome/dataset/ChEA+Transcription+Factor+Targets+2022 accessed March 2025) to search for targets of SMAD2, SMAD3, or SMAD4 among these genes. As predicted, *Tgfbi* featured as one of the top DEGs in this cell population, further supporting this gene as a direct target of TGF-β signaling in these cells. Top differentially upregulated SMAD2/3 targets in *Tgfbi*^+^ AJ cells included *Tnc*, *Pdgfra*, *Plxna4*, *Nrxn3*, and *Nnat* (Figure 6C). Targets such as *Pdgfra* and *Tnc* are particularly relevant in the context of the question addressed here. During lung morphogenesis, *Pdgfra^+^* cells are reported to be abundant in the mesenchymal compartment, including the domain of putative SM progenitors we identified in Cluster 2. *Pdgfra*^+^ cells in peribronchiolar and alveolar regions are known to differentiate into contractile aSMA^+^ cells in the developing lung and later shown to be crucial for alveolar septation (18, 30–32). Various lines of evidence support interactions of *Pdgf*, *Tgfb*, and *Tnc* in myofibroblast/fibroblast differentiation in different tissue contexts. For example, a functional TGF-β/PDGFRA signaling crosstalk regulates myofibroblast migration and differentiation (33). A mechanism in which PDGFRA and TGF-β signaling promotes TNC expression in intestinal myofibroblasts has been described in a mouse model of intestinal injury (34). This crosstalk also involved SMAD3 and SMAD4, which were shown to activate TNC promoter activity, and their overexpression further enhanced the effect of TGF-β (35). In addition, we found *Plxna4* among the most upregulated genes in *Tgfbi*^+^ cells (Figure 6, C and D). This gene encodes a receptor for semaphorins, known regulators of migration and neuron guidance. *Plxna4* is a TGF-β/SMAD3 target upregulated in SM cells and fibroblasts by *Tgfb1* (36, 37) (Figure 6D). None of the Cluster 2 genes upregulated by BMS in *Tgfbi*^+^ cells in B6 carried SMAD2, SMAD3, or SMAD4 binding sites (Supplemental Figure 11C). We confirmed the relevance of these findings and showed that the impact of RA signaling disruption on regulators of the SM program, PDGFRA and TNC, could be faithfully recapitulated in vivo embryos exposed to maternal BMS or control regimens as described below in subsequent experiments.

Next, we investigated whether blocking TGF-β overactivation could prevent the aberrant airway SM program of BMS-exposed embryos in vivo. We selected from a list of pharmacological compounds those proven to inhibit TGF-β signaling, with some evidence that they could be well tolerated during pregnancy, and able to cross the placental barrier without inducing embryonic lethality. Then different doses were tested to identify those that effectively interfered with TGF-β signaling in the embryo during organogenesis with minimal impact on overall embryo and organ growth. Special attention was given to identify maternal dosing regimens compatible with the developmental window of our study (E9.5–E14.5), applying criteria analogous to those we chose in our analysis of BMS in the lung (overall preserved epithelial patterning and growth).

Although none of the compounds screened were able to fulfil all criteria, we selected galunisertib (Gal) (LY2157299), a selective TGF-βRI kinase inhibitor, for its effectiveness, ability to cross the placental barrier, and interfere with TGF-β signaling in the embryo. Gal was administered to both AJ and B6 mothers at concentrations of 20, 75, and 150 mg/kg BW on gestation days 9.5–14.5, and embryos were analyzed subsequently (Supplemental Figure 15A). E14.5 embryos from AJ and B6 mothers exposed to 20 and 75 mg/kg/BW Gal were viable, but 150 mg/kg Gal consistently resulted in fetal death. Lungs from AJ embryos exposed to 20 mg/kg/BW Gal showed no obvious gross morphological defects compared to controls, but differed from those at 75 mg/kg/BW Gal, which appeared developmentally less mature (Supplemental Figure 15A). Disruption of TGF-β signaling by Gal was confirmed by downregulation of TGF-β targets *Tgfbi*, *Pdgfra*, *Tnc*, and *Myh11*, prominently at the highest concentration (Supplemental Figure 15B). Given the relatively preserved morphology and epithelial patterning of lungs with 20 mg/kg/BW Gal (no significant changes in *Epcam*, *Sftpc*, and *Sox2/Sox9*) compared to controls, we selected this dose for subsequent studies (Supplemental Figure 15, A and B).

**Figure 7:**
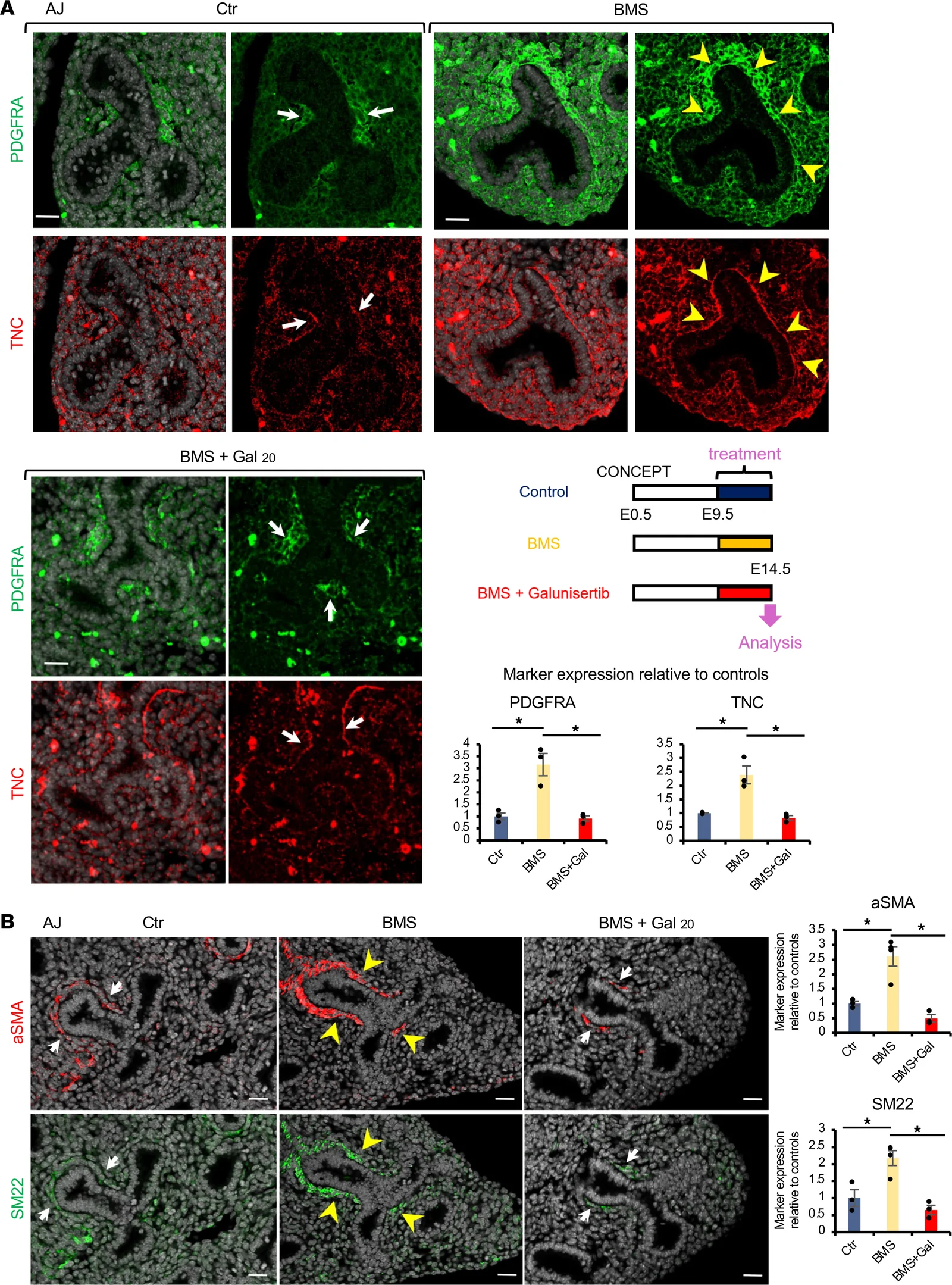
The aberrant airway SM program of prenatal RA signaling disruption in AJ lungs is prevented by TGF-β pathway inactivation in uterus. (**A**) Analyses of E14.5 AJ lungs exposed to maternal BMS, BMS+Gal, or control conditions. IF of PDGFRA and TNC in Control AJ showing typical expression pattern of these markers in distal mesenchyme associated with bud stalks (white arrows), distinct from BMS-exposed lungs, in which signals are much stronger and widely distributed (yellow arrowheads). Concomitant disruption of RA signaling and TGF-β inactivation in uterus prevents the aberrant PDGFRA and TNC expression of BMS-exposed lungs, as shown by their distinct pattern in BMS+Gal lungs comparable with controls. Graphs: Relative area of marker expression per field in AJ distal lung. Mean ± SEM (1-way ANOVA followed by Tukey’s post hoc test, *n* = 3 measurements per group). *P < 0.05. (**B**) IF of aSMA and SM22 in E14.5 AJ lungs showing that exposure to BMS+Gal prevented the aberrant ectopic SM formation in distal airways of embryos exposed to BMS alone. Graphs: Quantitative analysis of marker expression. Mean ± SEM (1-way ANOVA followed by Tukey’s post hoc test, *n* = 3–4 measurements per group). *P < 0.05. Scale bars: 20 μm.

Thus, we examined how simultaneous disruption of RA signaling and TGF-β inactivation influenced the airway SM program emerging in stalks of growing lung buds. AJ mothers were administered BMS (3.75 μg/g) plus Gal (BMS+Gal; 20 μg/g), BMS alone (3.75 μg/g), or oil (control) starting on gestation day 9.5, and embryonic lungs were analyzed on E14.5 (Figure 7A). IF of PDGFRA and TNC in controls confirmed their local signals distribution in the distal niche associated with initiation of the airway SM program (Figure 7A), consistent with their pattern of mRNA expression (Figure 4C and Supplemental Figure 10B). This contrasted with the marked increase in PDGFRA and TNC expression and ectopic expansion of their domain to adjacent areas found in BMS-exposed AJ lungs (Figure 7A). These changes were not found in BMS-exposed B6 embryos, further supporting the strain-related differences in RA regulation of the airway SM program (Supplemental Figure 16, A and B).

Notably, IF analysis of E14.5 lungs from mothers administered BMS+Gal showed a pattern of PDGFRA and TNC expression strikingly different from that of the BMS group, being rather comparable to that typically found in control lungs (Figure 7A). The data suggested that the aberrant expression and spatial distribution of regulators of the SM program (such as PDGFRA and TNC) that resulted from RA signaling disruption in AJ lungs could be at least partially prevented by restricting TGF-β signaling activation. Consistent with this, lungs from BMS+Gal-treated embryos showed no evidence of the increased and ectopic expression of SM markers (aSMA and SM22) seen in BMS-AJ lungs (Figure 7B).

Based on these findings, we asked whether the offspring from AJ mothers administered BMS+Gal, if followed through adulthood, would have normal pulmonary function and no AHR, the phenotype characteristic of the adult AJ mice exposed to BMS (alone) prenatally. However, in contrast with the high survival of AJ prenatally exposed to BMS, we found that many of the BMS+Gal AJ embryos were already not viable by E14.5 (only 4 embryos survived out of 6 litters) (Supplemental Figure 15C). Their minimal survival still at an early stage — (a) prior to crucial events that allow postnatal life, such as sacculation and alveolarization, combined with (b) the overly large-scale breeding required to generate enough surviving adults, (c) the need to perform pulmonary function tests, and (d) the need for appropriate replicates to reach meaningful statistical significance — altogether made these experiments unrealistic. We hypothesize that the high lethality observed likely resulted from a crucial but still undetermined requirement for TGF-β signaling in the embryo, since it was infrequent in embryos exposed to BMS alone.

Whether the regulatory network and mechanisms identified here extend beyond AJ mice to other genetic contexts remains unclear. We performed similar experiments in the outbred CD-1 mouse strain that we previously reported to develop AHR in adulthood if exposed prenatally to RA signaling disruption (10). Analysis of E14.5 CD-1 embryos from mothers administered BMS, Gal, or BMS+Gal under the same regimens showed a remarkable resemblance to what we observed in AJ mice. Consistent with our findings in AJ mice, E14.5 lungs from CD-1 embryos showed the distinctive BMS aberrant SM phenotype (e.g., increased ectopic PDGFRA and aSMA), which was not seen when TGF-β was inactivated by concomitant BMS+Gal exposure (Supplemental Figure 15D). These findings provide proof of principle that the RA/TGF-β regulatory network identified in AJ mice is not strain restricted but can also operate in distinct genetic backgrounds, including outbred populations.

Our data identify a mesenchymal cell niche exquisitely sensitive to RA during initiation of the SM program in the developing lung of AJ mice. We show that differences in genetic background profoundly influence this mesenchymal niche, as seen by the distinct strain response to RA disruption. Collectively, these observations emphasize the effect of genetic background on the impact of developmental perturbations on adult AHR, highlighting mechanisms in the SM program that mediate such responses.

## Discussion

Here, we investigated the impact of genetic background on the ability of the embryonic lung to undergo a normal program of airway SM differentiation in the presence of a transiently RA-deficient intrauterine environment, and the consequences of this prenatal micronutrient perturbation on postnatal lung function and its association with AHR in adult life. Using 2 well-characterized inbred mouse strains, AJ and B6, with contrasting susceptibility to develop AHR, a hallmark of asthma in humans, we demonstrate that inhibiting RA signaling during an early developmental window that encompasses initiation of the airway SM program results in transcriptional rewiring of a selected population of *Tgfbi^+^* mesenchymal progenitors in AJ lungs. This cell population resides at the stalk mesenchyme of distal lung buds, where epithelial progenitors transition from SOX9 to SOX2 expression and the airway SM program is first engaged. Despite similar proportions of this TGF-β–activating population in both strains, disruption of RA signaling in AJ results in robust upregulation of downstream SMAD2/3 transcriptional targets, including *Pdgfra* and *Tnc*, and in the acquisition of an aberrant differentiation signature in these SM progenitors. These changes are not seen in the B6 embryonic lungs similarly exposed to an RA-deficient intrauterine environment. These observations provide evidence of a previously unsuspected mechanism of how the genetic background influences the transcriptional landscape of the developing lung and its sensitivity to environmental perturbation.

The RA and TGF-β pathways intersect extensively during lung morphogenesis (12, 26). A central mechanistic insight emerging from our work is the AJ-specific hyperactivation of TGF-β signaling in *Tgfbi^+^* progenitors following RA inhibition. In B6, these same cells maintained expression of RA targets and showed minimal transcriptional response to BMS, suggesting intrinsic resistance to RA withdrawal. This difference is consistent with the idea that in AJ lungs, endogenous RA normally constrains TGF-β activity at the onset of airway SM differentiation, and its removal derepresses TGF-β targets that drive an abnormal SM program and airway remodeling. It is also possible that the strain-specific responses we observed may have arisen from differences in how progenitor cells balance plasticity versus stabilization of remodeling-associated programs. In this scenario, RA–TGF-β interactions could influence the activation of remodeling pathways and the underlying plasticity of mesenchymal progenitor states, which together contribute to long-term morphological and functional effects.

Quantitative trait locus mapping in mice has previously identified loci for AHR that colocalize with genes involved in ECM organization, growth factor signaling, and SM differentiation (3). Many of these loci are syntenic with human GWAS signals associated with asthma and lung function, suggesting a good agreement of our findings with human genetic data, and that our observations may be mechanistically relevant to human disease. In humans, asthma is a polygenic trait with substantial heterogeneity in morbidity, therapeutic response, and airway remodeling phenotypes. Common variants in *TGFB1*, *PDGFRA*, and ECM-related genes have been linked to asthma severity, airway wall remodeling, and reduced lung function (38–40). Notably, the *TGFB1* promoter polymorphism C-509T (rs1800469) in humans has been linked to increased *TGFB1* transcription and heightened AHR and asthma severity (41). Our findings link these human observations to a potential mechanistic framework in which prenatal micronutrient status, through modulation of RA–TGF-β crosstalk, can tip a genetically poised mesenchymal progenitor population toward a remodeling-prone state. The TGF-β/PDGFRA/TNC axis we identified as a core component of this dysregulated program has strong precedents in developmental and pathological remodeling contexts. PDGFRA is a canonical marker of embryonic lung fibroblasts and airway SM progenitors; lineage tracing demonstrates that *Pdgfra*^+^ cells populate peribronchiolar mesenchyme and can differentiate into contractile airway SM cells during development (18, 31, 32). In disease settings, PDGFRA^+^ fibroblasts are increased in the lungs of asthmatics and contribute to airway wall thickening (38). *Tnc* is a well-established TGF-β/SMAD target gene (35) and can be induced synergistically by PDGF and TGF-β signaling (34). Functionally, TNC promotes cell proliferation, migration, and integrin/PDGFR complex signaling in SM cells, and its expression is elevated in the airways and lavage fluid of patients with severe or refractory asthma (42). Variants near or within *PDGFRA*, such as rs1800810, have been linked to severe nonallergic asthma and enhanced airway remodeling (43). GWAS has also implicated *TNC* in asthma risk, with certain alleles affecting ECM stiffness and influencing bronchial mechanics (44). These convergent lines of evidence point to a shared network in which genetic variation affecting the regulation or responsiveness of TGF-β/PDGFRA/TNC signaling modulates susceptibility to structural airway remodeling. Our transcriptomic analyses also identified *Igf1* as a marker of the Cluster 2 mesenchymal cells (which also featured *Tgfbi*). IGF1 is a known mitogen for airway SM cells, promoting proliferation and hypertrophy, being also implicated in airway remodeling in asthma (19). Although we found increased *Igf1* expression in mesenchymal populations of AJ-BMS lungs, this gene was not specifically upregulated in *Tgfbi^+^* cells, raising the possibility that it may be regulated by RA directly or by an additional mechanism. Also notable was the finding of *Plxna4* as the most upregulated gene in *Tgfbi^+^* cells from BMS-AJ lungs. This was intriguing since upregulated PLXNA4 has been reported in a cohort of patients with refractory asthma (45), as well as found to be a locus associated with low FEV1 in asthmatic patients by GWAS (46).

Here we provide evidence of this airway SM progenitor regulatory network operating within a mesenchymal niche uniquely sensitive to local RA and TGF-β levels during lung morphogenesis, and profoundly shaped by genetic background across strains. Modulation of TGF-β signaling altered both the expression and spatial distribution of key regulators of the SM program, including PDGFRA and TNC, consistent with a localized RA/TGF-β network controlling this program. Although, due to technical limitations in our model, we were unable to demonstrate mechanistically that opposing the TGF-β effects of an RA-deficient intrauterine environment prevents AHR, our observations linking prenatal disruption of RA signaling with adult AHR in AJ and CD-1 suggest that the RA/TGF-β link we describe here is operational and likely to be relevant in vivo.

From a translational perspective, the identification of a discrete, RA-sensitive, TGF-β–activated progenitor population provides both a mechanistic link between prenatal nutrition and adult airway disease and a potential target for future studies aiming at early-life intervention. If analogous cells exist in the human fetal lung — and given the high conservation of developmental signaling pathways between mice and humans, this is likely — then individuals harboring risk alleles in *TGFB1*, *PDGFRA*, *TNC*, or related RA pathway genes may benefit from personalized nutritional care during pregnancy to ensure adequate micronutrient availability during the critical developmental windows. Our findings suggest that in genetically susceptible individuals, transient environmental perturbations — such as maternal vitamin A insufficiency — during a critical developmental window could trigger abnormal activation of pathways to permanently reprogram specific progenitor niches in a manner dependent on host genetics, predisposing to abnormal remodeling in response to later life. In this context, the observations reported here illustrate well the broader concepts of the developmental origins of health and disease.

In conclusion, our results support a model in which a genetically determined RA-sensitive mesenchymal progenitor niche acts as a developmental checkpoint for airway SM initiation (Supplemental Figure 17). In susceptible genetic backgrounds, transient loss of RA signaling unleashes a hyperactive TGF-β/PDGFRA/TNC program that persists beyond development, structurally remodeling the airway and manifesting functionally as AHR in adult life. Our model integrates genetic background, environmental exposure, and developmental signaling into a unified framework to support a model in which the RA/TGF-β regulatory axis is selectively engaged in genetic backgrounds permissive to AHR, and extend its relevance beyond inbred strains to outbred populations. Future work integrating single-cell genomics of human fetal lung, functional assays of risk allele impact, and longitudinal clinical cohorts with prenatal nutritional data could validate and extend these findings, ultimately enabling precision prevention strategies for airway disease rooted in developmental biology.

## Methods

### Sex as a biological variable.

Both male and female mice were included in this study, because sex was not considered as a biological variable for most embryonic analyses.

### Mice.

AJ and B6 mice were from The Jackson Laboratory (stock nos. 000646 and 000664), and CD-1 mice were from Charles River (stock no. 022). The morning a vaginal plug was detected was considered E0.5.

### Maternal administration of BMS and Gal.

Pregnant mice were orally administered BMS (B6688, Sigma-Aldrich) or corn oil from E9.5 to E14.5. Except for the dose-response study, all BMS experiments used 3.75 μg/g of BW per day. Gal (HY-13226, MedChemExpress) was orally administered to pregnant mice, alone or with BMS. Dams were sacrificed for embryonic lung analyses on E14.5 or allowed to deliver pups, which were maintained on a standard nutritionally complete rodent chow diet (15 IU vitamin A/g; PicoLab) until adulthood.

### MCh challenge and specific airway resistance measurements.

Specific airway resistance was assessed in 16-week-old male mice by whole-body plethysmography (FinePointe, DSI). Baseline resistance was measured after nebulized saline, followed by increasing concentrations of nebulized MCh as previously reported (10). Results were analyzed by 2-way ANOVA.

### Lung explant cultures.

E12.5 mouse lungs were cultured for 48 hours at 37°C in 5% CO_2_ on 6-well Transwell-COL dishes in BGJb-based medium (12591038, Thermo Fisher Scientific) (10) with or without BMS or SB431542 (1614, Bio-Techne) (detailed reagent list in Supplemental Table 16). Specimens were processed for qPCR or fixed for IF analysis.

### Quantitative reverse transcription PCR.

Total RNA was extracted from E14.5 lungs or lung explant cultures from AJ and B6 mice (RNeasy, QIAGEN, 74004). cDNA was generated and quantitative real-time PCR was performed using SYBR Green Master Mix on a LightCycler 480II (Roche). Expression was normalized to *Gapdh or Actb* (primers listed in Supplemental Table 17).

### IF.

Lungs from 16-week-old mice and E14.5 embryos were fixed in 4% paraformaldehyde at 4°C overnight, paraffin embedded, and sectioned (6–8 μm). Sections were blocked (1% BSA and 0.5% Triton X-100), incubated with primary antibodies at 4°C overnight, and then incubated with Alexa Fluor–conjugated secondary antibodies and DAPI (Supplemental Table 18). Embryonic lung explants processed for whole-mount IF were fixed in 4% paraformaldehyde, blocked as above, and incubated with primary antibodies (4°C overnight). Samples were then incubated with secondary antibodies and DAPI at 4°C overnight.

### Morphometric analysis.

Confocal microscopy (Zeiss LSM 710) was performed in lung sections stained with specific antibodies and analyzed at ×20 magnification (Aivia AI Image Analysis software, Leica Microsystems). Quantitative assessment for each staining was performed in 3 adult (3–5 random fields/airway) and 3 embryonic lungs (1–3 fields/airway whenever available). The area expressing the specific IF signals (pixels^2^) and/or number of expressing cells was calculated, and normalized to the length of their associated basement membranes. Whole-mount IF images of lung explant cultures were similarly acquired (Zeiss LSM 710 or Leica Stellaris 8) and analyzed using the SAMJ Annotator. At least 3 distal airways from these cultures were quantitated; areas expressing specific IF signals were measured and values were normalized to the corresponding airway basement membrane length.

### Tissue retinoid concentration analyses.

ROH and RE (retinyl oleate, linoleate, palmitate, and stearate) were measured by reverse-phase high-performance liquid chromatography (HPLC), as previously reported (47, 48), and identified by comparing the retention times and spectral data of the experimental compounds with those of authentic standards. Retinyl acetate (Sigma-Aldrich) served as an internal standard.

### Identification of RARE-OCRs in developing lungs.

ATAC-seq datasets from E14.5–E16.5 mouse lungs (Mouse ENCODE epigenomic data PRJNA63471, NCBI GEO) were downloaded and analyzed as shown in Supplemental Figure 2A to identify candidate RA-regulated genes associated with vitamin A status–dependent lung phenotypes. The irreproducible discovery rate (IDR) of the peaks between experimental replicates (2/time point) was calculated and the peaks with an IDR of less than 0.05 were used (IDR-OCRs). The annotation of the IDR-OCRs from their corresponding transcription start site (TSS) was performed and visualized using the ChIPpeakAnno package (https://www.bioconductor.org/packages/release/bioc/html/ChIPpeakAnno.html). IDR-OCRs were further screened for RARE (RARa (NR)/K562-RARa-ChIP-Seq (Encode)/Homer (motif 304) motif) using HOMER motif analysis (http://homer.ucsd.edu/homer/motif/).

### Bulk RNA-seq.

Total RNA from E14.5 AJ and B6 lungs (control, BMS *n* = 4 per group) were extracted and processed for library preparation and sequencing (Novogene Co., Ltd). Sequences were aligned to the mouse GRCm39 reference genome using the STAR aligner (https://github.com/alexdobin/STAR). Transcript counts were analyzed with DESeq2 (https://www.bioconductor.org/packages/release/bioc/html/DESeq2.html). DEGs were defined as genes with an absolute log_2_(fold change) of greater than 0.58 and a false discovery rate–adjusted (FDR-adjusted) *P* value of less than 0.05. Sequencing and alignment statistics are provided in Supplemental Table 15.

### Transcription factor activity analysis.

Transcription factor activity was assessed using the ULM approach implemented in *decoupleR*. We used the DESeq2-processed bulk RNA-seq datasets, and the CollecTRI mouse regulatory network was employed as the reference for transcription factor target interactions (49). Significant transcription factor activities were defined by an adjusted *P* value of less than 0.05.

### Single-nucleus multiome analysis.

Gene expression and chromatin accessibility were assessed at single-nucleus resolution using the 10X Genomics single-cell Multiome platform in E14.5 male AJ and B6 lungs from control and BMS treatment groups. Embryonic lungs from 4 B6 or 6 AJ mice were pooled, and nuclei were collected, counted, and inspected under a microscope. Multiome libraries were prepared according to the 10X Genomics Chromium Next GEM Single Cell Multiome ATAC + Gene Expression user guide. The snATAC-seq and snRNA-seq libraries were sequenced (Illumina NextSeq 2000) using the 50-8-24-49 and 28-10-10-90 formats, respectively, aiming for 25,000 snATAC-seq and 20,000 snRNA-seq reads per cell to ensure data coverage. Raw FASTQ files were processed using Cell Ranger ARC and aligned to the mm10 reference genome. Subsequent analyses were conducted in R (https://www.r-project.org/) using Seurat and Signac (https://stuartlab.org/signac/). Quality control excluded low-quality cells. Cells were filtered based on low total RNA counts (nfeature_RNA >300, except AJ control >750), low total ATAC-seq counts (nfeature_ATAC>300), high mitochondrial gene expression percentage (<0.2), nucleosome signal greater than 2, and TSS enrichment score less than 1. Highly variable genes (*n* = 3,000) were identified, and data were scaled. To integrate data across all experimental conditions, we employed Seurat’s integration workflow. PCA was performed on the integrated data. Uniform Manifold Approximation and Projection (UMAP) dimensionality reduction was then applied using *RunUMAP*. ATAC-seq data were processed using Signac (v1.14). Feature selection was performed using *FindTopFeatures*, retaining peaks present in at least 50 cells. Data were normalized, and dimensional reduction was performed using singular value decomposition (SVD). ATAC-seq data were integrated across experimental conditions using the latent semantic indexing (LSI) algorithm in Seurat. Multimodal integration and clustering were performed using Seurat’s WNN approach, followed by UMAP visualization and Leiden clustering. Clusters were annotated using known cell type–specific markers and *FindAllMarkers* results. DEG and DAR analyses were conducted separately for AJ and B6 strain samples, using *FindMarkers* in Seurat. DEG analysis used MAST (50), and DAR analysis used the likelihood ratio test. Significance was defined as adjusted *P* value of less than 0.05 and log_2_(fold change) greater than 0.58 or less than –0.58. We identified the genes closest to the DARs using the *ClosestFeature* function in Signac.

### Statistics.

Continuous variables were analyzed using 2-tailed Student’s *t* tests for comparisons between 2 groups and 1-way ANOVA followed by Tukey’s post hoc test for comparisons among more than 2 groups, unless otherwise specified. Categorical variables were analyzed using Fisher’s exact test. Specific airway resistance data were analyzed by 2-way ANOVA. For single comparisons, *P* less than 0.05 was considered statistically significant; for analyses involving multiple testing, FDR-adjusted *P* less than 0.05 was considered statistically significant.

### Study approval.

All mouse experiments were approved by the Columbia University Institutional Animal Care and Use Committee (WVC, IACUC AC-AABF2567) or the Albert Einstein College of Medicine Institutional Animal Care and Use Committee (MS, IACUC 00001232) and followed ARRIVE guidelines.

### Data availability.

All data used in the figures are represented in the Supporting Data Values file. Raw data of the bulk RNA-seq and snMultiome (snRNA-seq + snATAC-seq) analysis performed for this study are available in the NCBI GEO database under accession number GSE307765.

## Author contributions

Conceptualization and methodology: WVC and MS. Investigation: TO, ZC, YS, AKSK, BDK, XK, YKK, PR, YM, SMS, and MS. Formal analysis and software: TO, ZC, AKSK, BDK, and MS. Writing — original draft: MS and WVC. Writing — review and editing: all authors. Supervision: LQ, WVC, and MS.

## Conflict of interest

The authors have declared that no conflict of interest exists.

## Funding support

This work is the result of NIH funding, in whole or in part, and is subject to the NIH Public Access Policy. Through acceptance of this federal funding, the NIH has been given a right to make the work publicly available in PubMed Central.

- Internal Texas A&M AgriLife Research funds (to MS).
- NIH award numbers R01HL145302 (to MS), R01DK136989 (to MS), R01HD094778 (to LQ), and R01EY027405 (to LQ), and R35 HL166661-01 (to WVC).

## Supplementary Material

Supplemental data

Supplemental tables 1-18

Supporting data values

## Version 1 07/21/2026

In-Press Preview

## Version 2 09/08/2026

Electronic publication

## Version 3 09/09/2026

The graph in Figure 5B was revised to correct a cropping error.
