# Supplementary material for: Genetic background influences developmental airway smooth muscle program and susceptibility to airway hyperresponsiveness in mice
Source: JCI Insight. 2026 Jul 21;11(17):e200486. doi: 10.1172/jci.insight.200486 (PMC13557617; doi:10.1172/jci.insight.200486)
Supplement: Supplementary file 1 [file jciinsight-11-200486-s295.pdf]

SUPPLEMENTAL FIGURE LEGENDS

Supplemental Figure 1. Differential effects of prenatal disruption of RA signaling by maternal BMS-administration in AJ and B6 mice.

(A) Impact of maternal BMS administration at different concentrations (3.75-15 µg/body weight per day (µg/bw/day) on the embryo gross morphology and lungs at E14.5 in both strains. Diagram experimental design: Control (corn oil) or BMS-containing diet administered from gestation day 9.5-14.5 to AJ and B6 mothers. Body truncation and lung hypoplasia at the highest maternal BMS concentration most prominent in AJ embryos compared to B6, not seen at 3.75 µg/bw/day in either strain. Both controls (corn oil) and BMS (3.75 µg/bw/day) offsprings reached adulthood.

(B) Hematoxylin and eosin (H&E) staining of representative sections from E14.5 control and BMS-exposed embryos. Maternal administration of BMS at 3.75ug/g/bw results in embryos morphologically similar to controls showing none of the pleiotropic effects of those exposed to BMS at 7.5 ug/bw/day. Left panel depicting frontonasal process (fnp) truncation, defective forebrain (fb) and heart (hrt) development and pronounced lung (lu) hypoplasia of AJ embryos exposed to BMS at 7.5 ug/bw/day, not seen in BMS AJ embryos at 3.75ug/g/bw. Right panel showing the pleiotropic effects of BMS in B6 embryos (lung hypoplasia) only at 7.5 ug/bw/day.

(C-D) Effects of prenatal BMS exposure (3.75ug/g/bw) in adult AJ and B6 offsprings: body weight, lung and liver weight relative to body weight. Graphs are mean ± SE, Two-tailed Student's t-test, *$p<0.05$. AJ Control (n=6), AJ BMS (n=6), B6 Control (n=9), B6 BMS (n=6).

Supplemental Figure 2. Differential expression of RA-responsive genes in embryonic lungs from AJ and B6 mice underlies the basis for the functional differences between these strains.

(A) Schematic overview: workflow for identification of RA–responsive open chromatin regions (RARE-OCRs) enriched in the embryonic lung during early differentiation (middle). Identification of RARE-OCRs, from E14.5-E16.5 mouse lung ATAC-seq datasets (ENCODE epigenomic PRJNA63471, Gene Expression Omnibus, NCBI). Irreproducible discovery rate (IDR) of the peaks between the experimental replicates and the peaks with IDR<0.05 were used. IDR-OCRs annotation from their corresponding transcription start site (TSS) was performed and visualized using the ChIPpeakAnno package. Promoter regions defined as within <2,000 bp upstream and <1,000 bp downstream the TSS (mm10 KnownGene - UCSC Genome Browser used to identify the TSS location). IDR-OCRs were further screened for presence of RARE (RARa (NR)/K562-RARa-ChIP-Seq (Encode)/Homer (motif 304) motif). Venn diagram (left): overlap of IDR-OCRs in embryonic mouse lungs. A total of 34,039, 26,128, and 45,988 IDR-OCRs were identified at E14.5, E15.5, and E16.5, respectively, with 22,812 regions shared across all three timepoints. While most IDR-OCRs were detected at all three time points, we defined RARE-OCRs as those detected as IDR-OCRs at two or more time points. Pie chart (right): Among 28,355 RARE-OCRs, 44.2% were identified within the promoter regions of genes.

(B) Bulk RNAseq analysis of E14.5 AJ vs B6 (controls only) identifies 1258 DEGs and strain-specific functional pathways potentially regulated through RARE-containing genes. Volcano plot highlighting *Lrat* upregulated in AJ. Gene Ontology molecular function enrichment analysis of differentially expressed genes containing RARE motifs. Bar plots: significantly enriched GO terms (*p*adj < 0.05) for top RARE-OCR genes in each strain (AJ-high genes and B6-high genes), ranked by significance.

(C) Bulk RNAseq of BMS vs control E14.5 AJ and B6 lungs. Left: Venn diagram indicating the number of DEGs downregulated or upregulated by BMS from each strain. DEGs downregulated in both strains include markers of RA activity (*), genes harboring RARE-OCR (bold) and DEGs in controls (red: AJ; blue: B6).

(D) Bulk RNA-seq analysis (*Lrat, Stra6, Dhrs3)*: whole E14.5 lungs from AJ and B6 embryos exposed to maternal BMS (3.75ug/g/bw) diet: downregulation by BMS in both strains and increased *Stra6* and *Lrat* expression in control AJ compared to B6 control. qPCR: expression of RA pathway components (*Lrat*, *Stra6*) in lung homogenates from E14.5 AJ and B6 embryos. Significant *Lrat, Stra6* downregulation by BMS in both strains. *Lrat and Stra6* expression significantly higher in control AJ compared to control B6. *$p<0.05$. **$p<0.01$.

(E) qPCR analysis showing Hox family member genes downregulated in BMS-exposed E14.5 lungs from both AJ and B6 embryos. Graphs: n=4 per group.

(F) Gene Set Enrichment Analysis (GSEA) of DEGs in BMS vs control E14.5 lungs from B6 mice. Dot plots: significantly enriched Gene Ontology terms for biological processes (BP) and molecular functions (MF) displayed by direction of enrichment (activated vs. suppressed pathways). GO terms enriched in AJ BMS include suppression of RA-related molecular function (RA binding) and activation of biological processes related to muscle contraction and microfilament activity (Supplemental Figure 3A). No molecular function GO terms were detected in B6, suggesting a less robust RA-related functional activity.

Supplemental Figure 3. Differential activation of a SM gene regulatory network under RA-deficiency in AJ developing lungs.

(A) Gene set enrichment analysis. Significantly enriched GO terms ($p$adj < 0.05) ordered by GeneRatio in AJ lungs; (*) highlight terms associated with muscle function.

(B) Bar charts depict the most differentially activated TFs in BMS vs Control. Positive scores (red): increased activity in BMS; negative scores (blue) increased activity in control (*p<0.05, **p<0.01, ***p<0.001). Panels show String DB network pathways altered by BMS: increased activity of muscle tissue development-related TFs in BMS-AJ but not BMS-B6 lungs. Red nodes: TFs with significantly elevated activity by BMS.

Supplemental Figure 4. Single-nuclei multiomics shows similar shifts in cell proportion in BMS-exposed AJ and B6.

(A) UMAP visualization of single-nucleus multiome data from E14.5 lungs showing the main cell populations identified by established markers.

(B) Relative cell type proportions. No significant differences in response to BMS (permutation testing n = 10,000). Cutoffs of FDR < 0.05 and absolute log2fold difference > 0.58.

Supplemental Figure 5. Multiome analysis reveals significantly higher number of DEGs and open chromatin regions in AJ than B6 lungs in response to intrauterine RA signaling disruption.

(A) Split UMAP projections displaying the distribution of the four major cell populations across conditions (control and BMS in AJ and B6 lungs).

(B) Volcano plots showing differentially expressed genes (DEGs) and differentially accessible chromatin regions (DARs) in epithelial, mesenchymal, and endothelial cells in response to BMS in AJ and B6 lungs. Significantly higher number of DEGs and DARs in AJ compared to B6. Mesenchymal compartment showing the greatest number of DEGs/DARs (boxes).

Supplemental Figure 6. Distinct subpopulations of ECM-producing, vascular and airway SM precursors in the mesenchymal compartment of developing lung.

(A) U-map (left) overview cluster analysis of mesenchymal compartment from all groups. Cluster 0 (undifferentiated proliferative) signature characterized by cell cycle-related genes (*Top2a, Kif15, and Mki67*), visualized by dot plot (top right), violin plots, and feature plots. In situ hybridization (ISH) showing diffuse transcript distribution throughout the E14.5 lung mesenchyme.

(B-C) Dot plots: enrichment of mitochondrial genes in cluster 4 and extracellular matrix-associated genes in cluster 6.

(D-E) Dot plot of top Cluster 5 genes featuring *Pdgfrb, Heyl, Notch3, Cspg4* identified by their association with the vascular smooth muscle (SM) program. Violin plots, feature plots and ISH images of representative cluster 5 genes. Venn diagram showing distinct and shared gene expression of clusters 5 (vascular) and 7 (airway) SM populations. Images depict striking difference in spatial distribution of *Myom1* (cluster 7) and *Pdgfrb, Heyl, Notch3* (cluster 5) reflecting the distinct airway and vascular SM signatures in the E14.5 lung mesenchyme.

Supplemental Figure 7. Multiome analysis identifies a distal mesenchymal population enriched in regulators of cell migration and tissue patterning genes.

(A) Top differentially enriched genes of Cluster3 (left). Table: most significant terms ranked by adjusted p value (Enrichr, Descartes Cell Types and Tissue 2021 library). Terms in red are lung-related. Violin and feature plots of representative cluster 3 genes (*Sema3d, Epha3 and Ptn)*. ISH showing most distal expression in the subpleural mesenchyme.

(B and C) Table depicting additional components of the Cluster 3 signature (left) featuring key regulators of lung pattern formation and mesenchymal differentiation (right: GO enrichment bar plot and terms listing these components). Genes shown in red in the tables are representative marker genes for Cluster 3. Gene ontology (GO) enrichment analysis reveals strong association with cell migration, morphogenetic movements and chemoattraction reported mostly in neuronal studies. *Wnt2* and *Fgf10* identified in Cluster 3 by dot plot, violin plot and feature plot. ISH of *Wnt2, Fgf10, Rspo2, Cttna2* showing distal mesenchymal localization, including subpleural region.

(D) Proposed developmental stages of airway SM differentiation based on our data and a model previously reported (Kumar et al. 2014). A three-stage progression from cluster 3 (undifferentiated tip bud mesenchymal progenitors) and cluster 0 (proliferative progenitors), through cluster 2 (recruitment zone), culminating in mature ASM in cluster 7 (maturation zone).

Supplemental Figure 8. GO analysis of cluster 2 and cluster 7 markers from E14.5 lung mesenchyme.

(A-B) GO terms enriched in each cluster. Red text indicates GO terms related to smooth muscle or smooth muscle differentiation.

Supplemental Figure 9. Differentially expressed genes between AJ and B6 control E14.5 lungs reflect strain differences in genetic background.

(A) Volcano plots: greater number of DEGs in cluster 0 (478), cluster 2 (273), and cluster 3 (161), with fewer DEGs detected in the remaining clusters. The total number of cells per cluster is indicated. Significantly upregulated or downregulated genes ($|log2FC| > 0.58$, $p$adj < 0.05) are represented in red.

(B) *Rarb* expression across mesenchymal clusters in AJ and B6 controls (dot plots and violin plots). No statistically significant difference in *Rarb* between AJ and B6 in any cluster.

(C-F) Identification of differentially upregulated and downregulated genes between control AJ and B6 lungs in clusters 0, 2, and 3. Heatmaps and table of the gene expression signature of individual clusters (**C,D)** and whole lung mesenchyme (clusters 0-9). The total cells per strain per cluster are shown in the heatmaps. **(E)** reveals common genes emerging as top DEGs across all mesenchymal clusters (Ex. *Mgp, Negr1, Hmga2, Col1a1, Opcml*) suggesting a shared transcriptional signature that distinguishes AJ from B6 mesenchymal cells. Several of these genes were also identified as significantly enriched in control AJ or B6 by bulk RNA-seq (whole lung), highlighting concordant strain-specific gene expression differences **(F)**.

Supplemental Figure 10. Analysis of the lung mesenchymal compartment (all mesenchymal clusters) identifies major differences in gene expression between AJ and B6 in response to prenatal disruption of RA signaling.

(A) Top, Diagrammatic U-map representation. Tables of DEGs (BMS vs control) confirming disruption of

RA signaling in both strains (*Rarb*) but distinct upregulation of markers and regulators of the SM differentiation program in AJ, compared to B6.

(B) U-Map projections and spatial distribution of representative DEGs (table) showing expression associated with emerging SM program.

Supplemental Figure 11. Transcriptomic responses of *Tgfbi*+ cells to BMS in AJ and B6 lungs.

(A) Markedly different number of DEGs in *Tgfbi*+ cells between control and BMS-exposed AJ (top) and B6 (bottom) lungs.

(B) Identification of cluster 2-enriched genes among those downregulated by BMS from whole-mesenchymal analysis of AJ and B6 lungs. Data represent the $\log_2$(fold change) in expression between BMS and control across mesenchymal clusters (x-axis) against the $\log_2$(fold change) of cluster 2 genes (y-axis).

(C) Identification of cluster 2-enriched genes among the DEGs upregulated by BMS in *Tgfbi*+ cells of B6 lungs (adjusted *p*-value <0.05 and log2 Fold Change > 0). Plot represents the $\log_2$(fold change) in expression between BMS and control conditions in *Tgfb*i+ cells (x-axis) against the $\log_2$(fold change) of cluster 2 genes (y-axis). None of the DEGs found carried Smad2, Smad3, or Smad4 binding sites.

Supplemental Figure 12. Tgfb signaling is selectively overactive in a distal population of smooth muscle progenitors of the BMS-cultured AJ lung.

(A) Diagram experimental design: E12.5 lung explants from AJ and B6 embryos cultured under control and BMS analyzed at 48h. qPCR of RA pathway components confirming downregulation of *Rarb* and *Cyp26b1* by BMS in both strains. Graphs: mean ± SE, **p*<0.05. (Two-tailed Student's t-test, n=3 per condition)

(B) αSMA whole mount IF of 48h lung embryonic explant cultures from AJ and B6. Arrows depict typical distal signal distribution in nascent airways (white) of controls AJ and B6 and increased aSMA accumulation in BMS AJ cultures (yellow). Graph: Quantitative analysis of aSMA. Relative area of staining per basement membrane: mean ± SE, **p*<0.05. (Two-tailed Student's t-test, n=3 per condition)

(C) IF of phosphorylated and total Smad2-3 and Sox2 in 48h control lung cultures. Arrows depict sites of Smad2-3 phosphorylation in stalks in the distal mesenchyme where newly-formed airways emerge.

(D) pSmad2-3 expression pattern in AJ lung explants cultured for 48h in Ctr or BMS media. Boxed areas depict signals in a distal mesenchymal population of both control and BMS explants (arrowheads; also shown in **C** and co-stained with total Smad2-3), but much stronger and focal in the distal mesenchyme of BMS lungs. Similar analysis in lung explant cultures from B6 shows no difference in pSmad2-3 expression between Ctr and BMS lungs. Graph: Relative area of staining for pSmad2-3 per basement membrane in airways under each condition. Graph: mean ± SE, Two-tailed Student's t-test, **p*<0.05. AJ Control (n=4), AJ BMS (n=6), B6 Control (n=5), B6 BMS (n=4).

(E) qPCR analysis of TGFβ targets (*Pdgfra*, *Tnc*, *Plxna4*) in homogenates control and BMS-treated AJ and B6 lung explants. Selective activation of Tgfb signaling confirmed by significant upregulation of TGFβ targets by BMS only in AJ. (Two-tailed t-test, n=4 per condition)

Supplemental Figure 13. Tgfb pathway components expression across mesenchymal clusters.

Tgfb2, *Tgfbr2*, and *Tgfbr3* enrichment in cluster 2 visualized by dot plots and violin plots. In situ hybridization: *Tgfbr3 signals* at prospective sites of airway SM initiation in distal mesenchyme (arrows).

Supplemental Figure 14. Strain-specific transcriptional response of *Tgfbi*⁺ cells to BMS exposure.

(A) UMAP representation of the *Tgfbi*+ cell population in AJ and B6 control and BMS-exposed lungs. The *Tgfbi*+ cell proportion differs between AJ and B6 controls, but remains largely unchanged by BMS in both strains (FDR < 0.05 in permutation testing, n=10,000). Violin Plot: *Tgfbi* expression in mesenchymal cells from all groups.

(B) Differential gene expression analysis of BMS vs Control *Tgfbi*+ cells from AJ and B6 lungs. Table depicts top 40 of the 79 differentially upregulated genes in AJ contrasting with only 3 genes upregulated in B6. Cluster 2 genes are highlighted in red.

(C) Markers of RA activation (Rarb and Cyp7b1) in *Tgfbi*+ cells showing downregulation by BMS in AJ but not in B6 (*p*adj <0.05).

Supplemental Figure 15. Effects of inactivating Tgfb signaling in uterus by maternal administration of Tgfb inhibitor.

(A) Impact of maternal administration of Galunisertib (Gal) at different concentrations (20mg/kg/bw, 75mg/kg/bw, 150mg/kg/bw) on the gross morphology of E14.5 embryos and lungs in AJ and B6. Diagram experimental design: control (corn oil) or Gal from gestation days 9.5-14.5 to AJ and B6 adult mice. No

viable embryos were recovered from Gal at 150mg/kg/bw treatment. At 75mg/kg/bw embryos were smaller and lungs hypoplastic, particularly in AJ, but overall appeared morphologically more preserved at 20mg/kg/bw Gal, compared to controls.

(B) Expression of epithelial markers (*Sox2*, *Sox9*, *Epcam*, *Sftpc*) and Tgfβ target genes (*Tgfbi*, *Pdgfra*, *Tnc*, *Myh11*) in lung homogenates from Gal-exposed E14.5 AJ embryos (qPCR). No significant (ns) differences in epithelial marker expression between control and Gal 20mg/kg/bw. Downregulation of Tgfβ targets in lungs treated by Gal 20mg/kg/bw and 75mg/kg/bw. Graphs: mean ± SE, *p<0.05 (n=3 per group).

(C) Changes in maternal body weight of adult AJ mice at gestation day 9.5 (drug administration) and 14.5 (analysis) relative to gestation day 0.5 under the various treatments. Inability to gain weight after Gal administration reflected the inability of AJ mothers to carry on normal gestation or maintain fetal viability, in contrast to BMS-treated mothers, which continued to gain weight by the time of analysis. (n=3 per group)

(D) Impact of Tgfb inactivation in the aberrant airway SM phenotype of E14.5 CD1 embryos exposed to BMS. Diagram experimental design: maternal administration of BMS, Gal and BMS+Gal. Panel: IF analysis of E14.5 lungs for epithelial markers of distal (SOX9) and proximal (SOX2) cell fate, and representative markers associated with initiation of the airway SM program (aSMA and PDGFRA). Controls exhibit typical aSMA and PDGFRA distribution of signals in mesenchyme of stalk lung buds (arrows) where distal airways emerge. The aberrant expression of these markers in lungs from CD1 embryos exposed to BMS (arrowheads) is not found BMS + Gal lungs which show aSMA and PDGFRA expression pattern comparable to controls. These signals are markedly inhibited in lungs exposed to Gal

(alone), suggesting that Tgfb overactivation is a key component of the abnormal SM program induced by disruption of RA signaling in developing lung.

Supplemental Figure 16. PDGFRA and TNC expression in E14.5 B6 and AJ lungs.

(A) IF of PDGFRA and TNC in B6 showing no difference in expression between control and BMS groups.

(B) Quantitative analysis represents the significant differences in marker expression between control and BMS in AJ but not in B6 lungs. Graph: Mean ± SE (Two-tailed Student's t-test, n=3 measurements per group). *$p<0.05$; ns: non-significant**.**

Supplemental Figure 17. Diagram Proposed model.

A genetically determined RA-sensitive mesenchymal progenitor niche acts as a developmental checkpoint for airway SM initiation. In a susceptible genetic background (AJ), prenatal transient loss of RA signaling unleashes an aberrant SM program likely mediated by hyperactive TGFβ signaling that persists beyond development, structurally remodeling the airway and manifesting functionally as AHR in adult life. This aberrant program is not seen in mice from a non-susceptible genetic background (B6) when subjected to similar prenatal disruption of RA signaling.

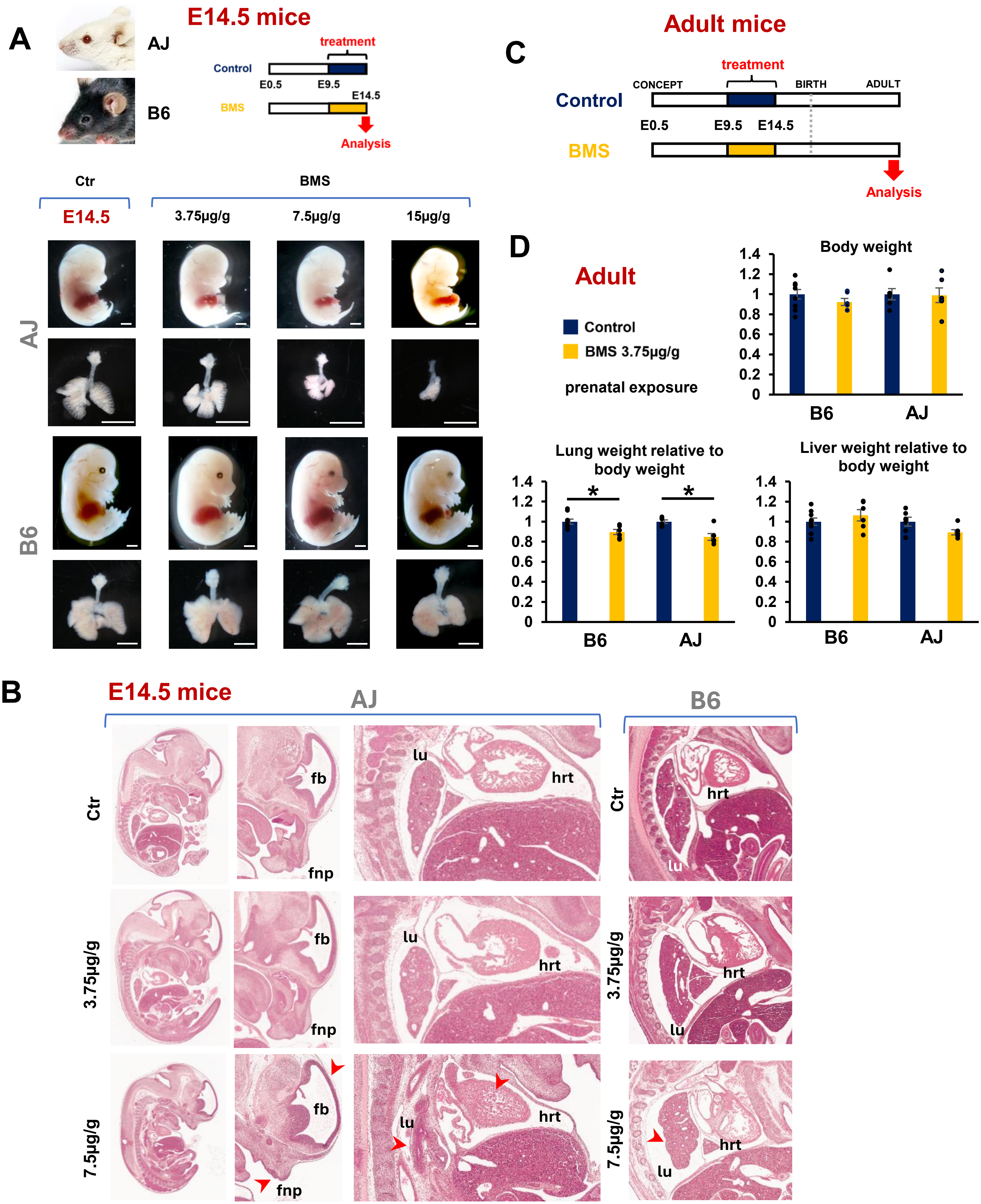

Suppl. Figure 1
Otoshi et al.

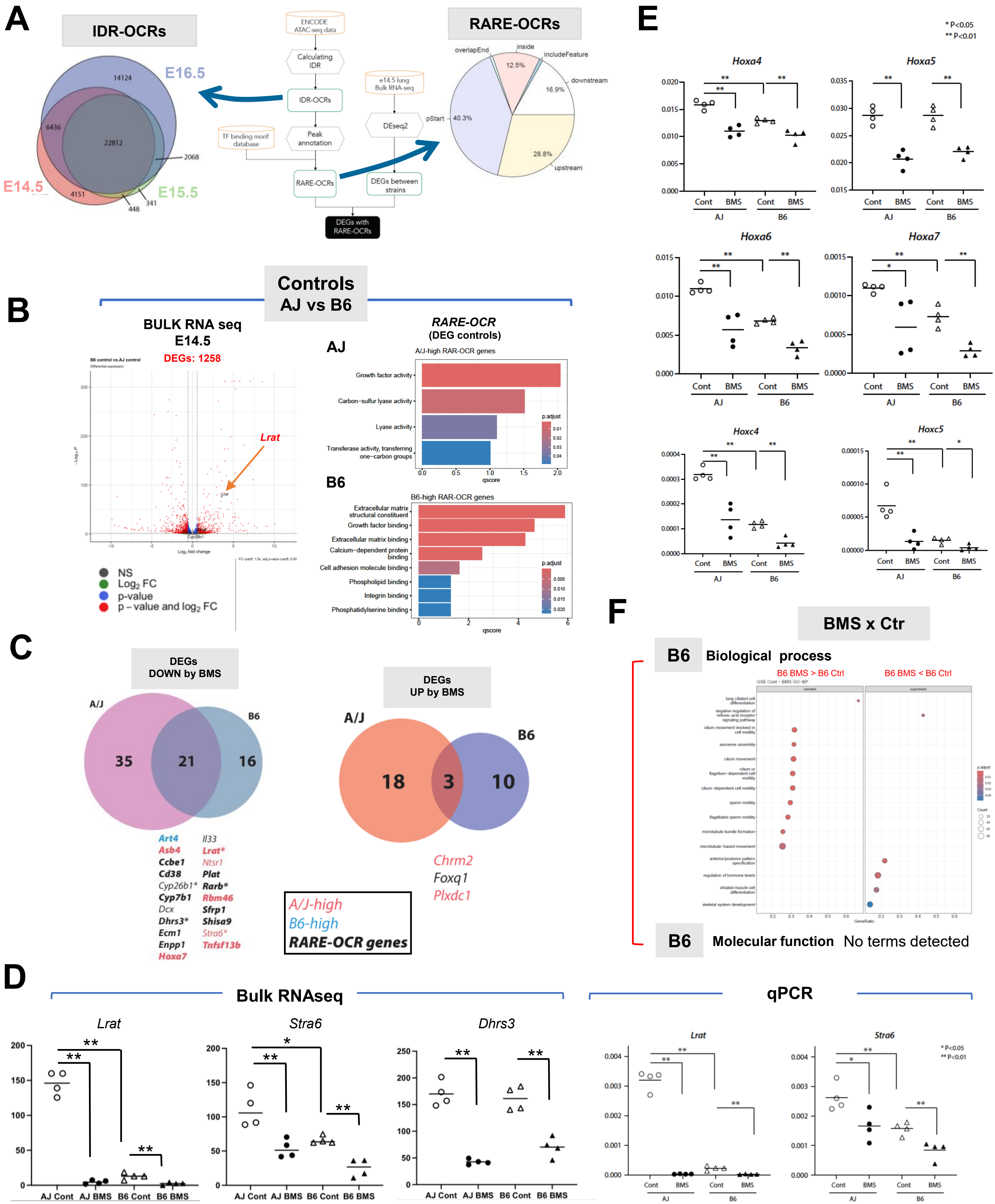

Suppl. Figure 2
Otoshi et al.

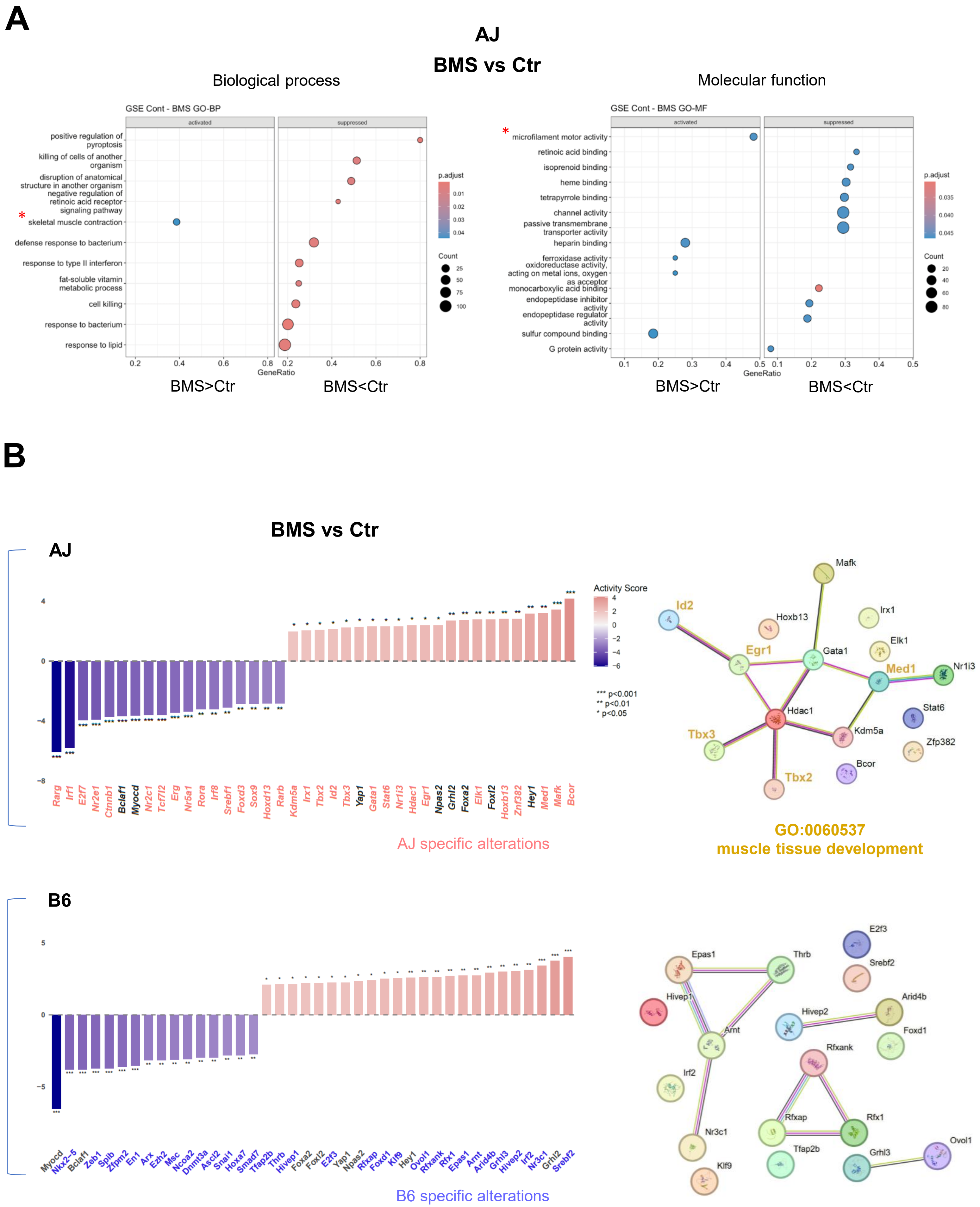

Suppl. Figure 3
Otoshi et al.

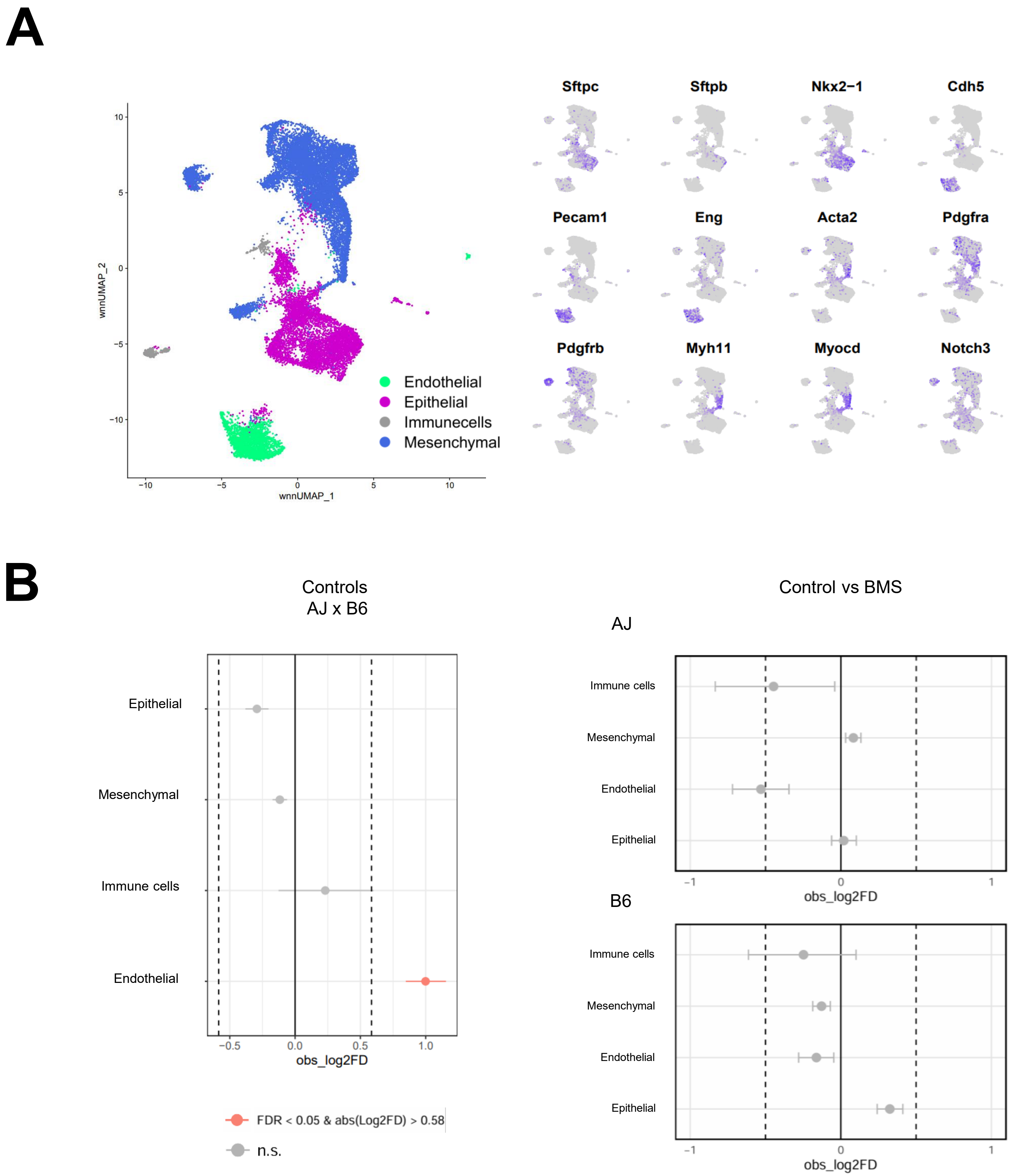

Suppl. Figure 4
Otoshi et al.

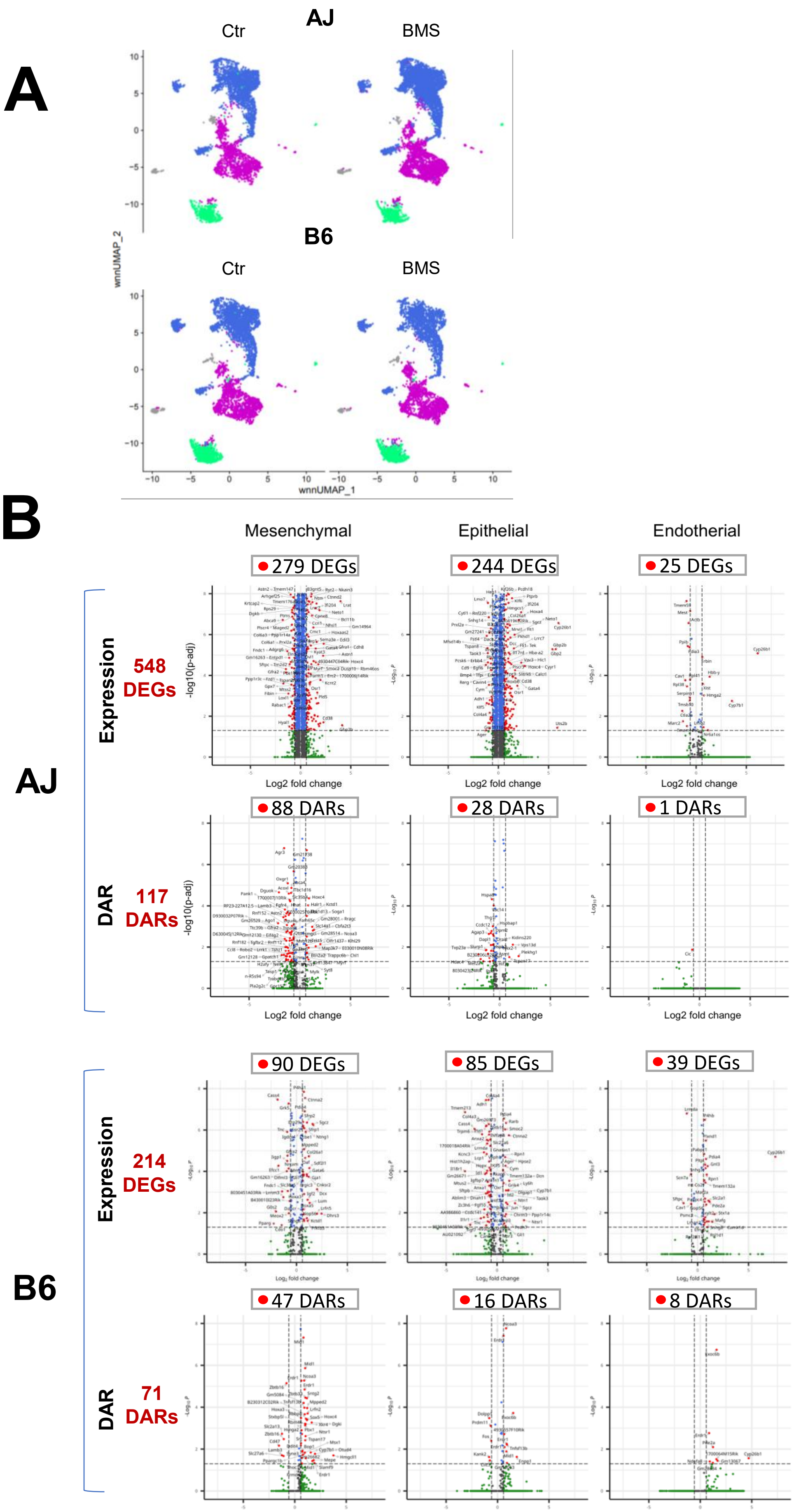

Suppl. Figure 5
Otoshi et al.

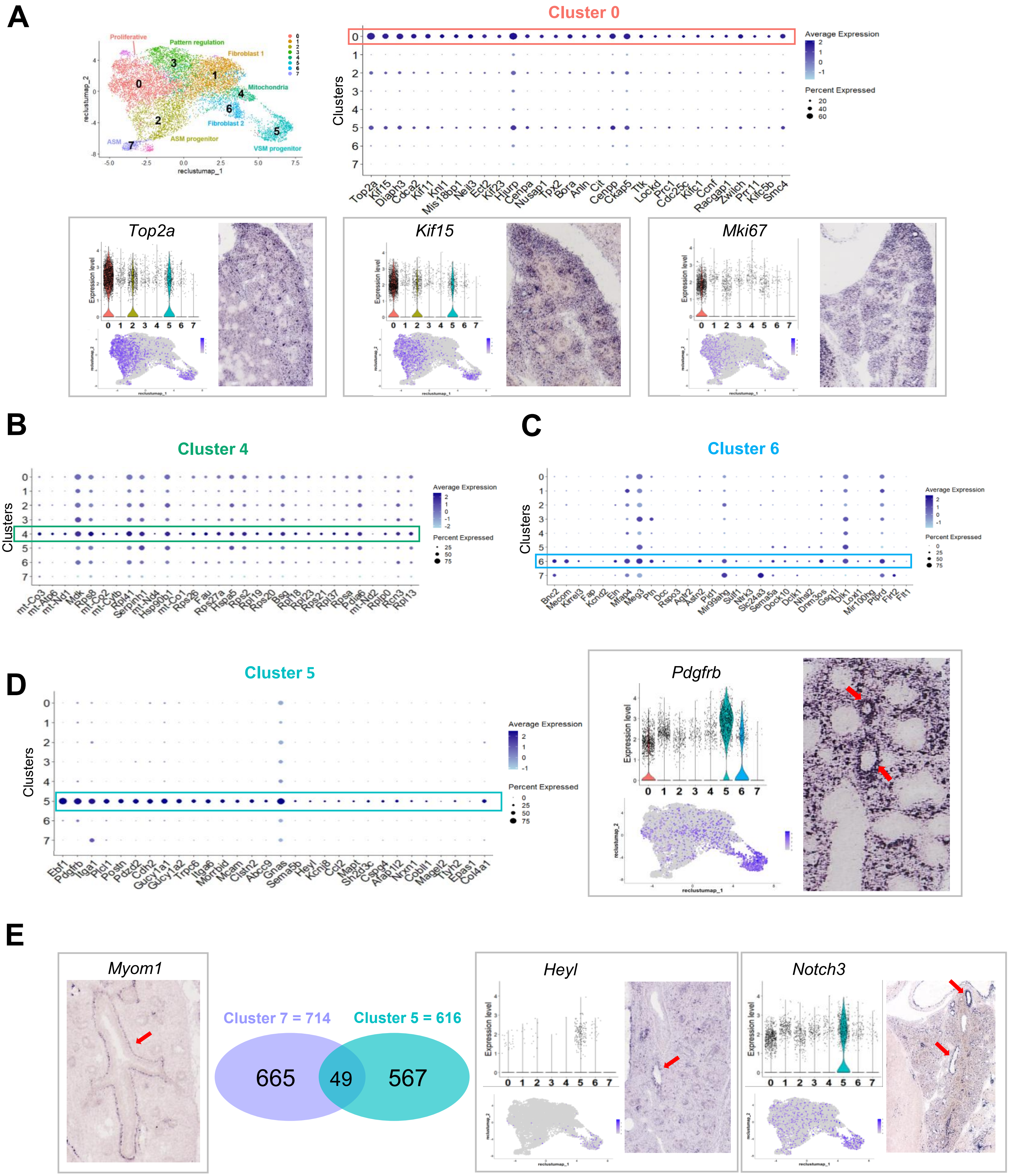

Suppl. Figure 6
Otoshi et al.

A

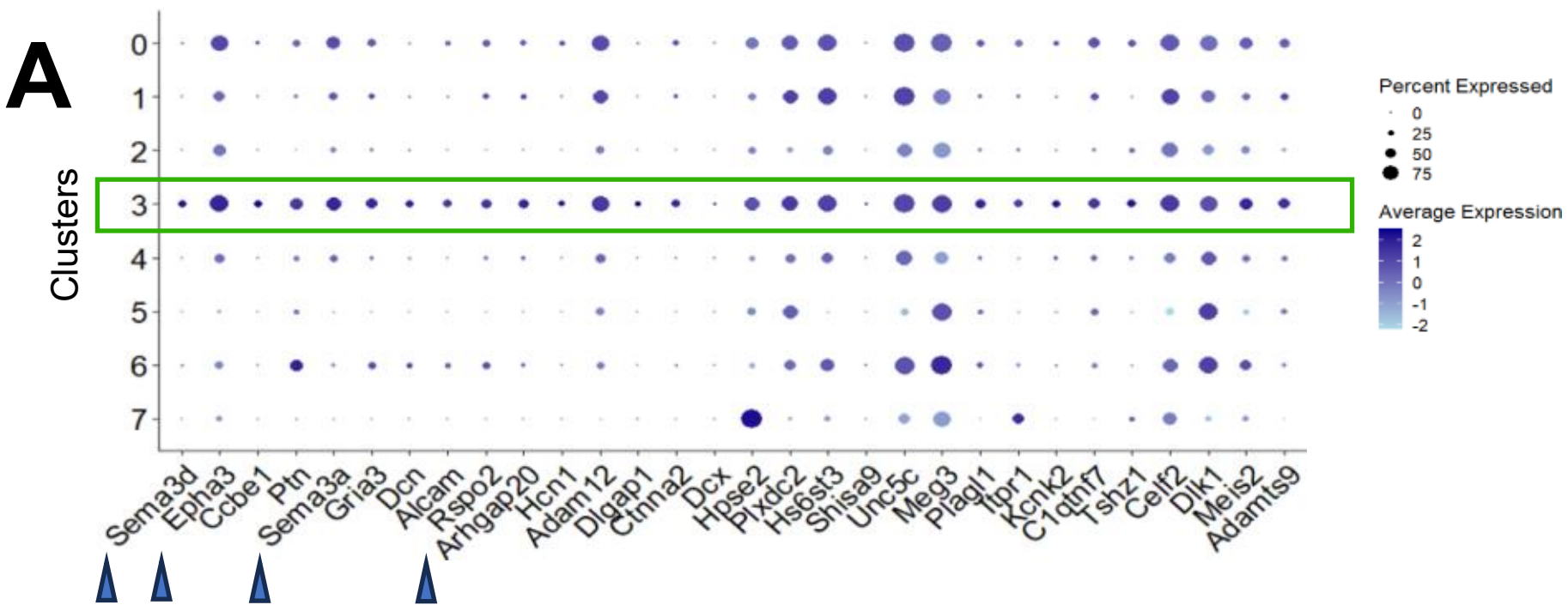

Descartes Cell Types and Tissue 2021

Name	Adjusted p value
Visceral neurons in Lung	0.000006813
ENS neurons in Stomach	0.0006165
Stromal cells in Thymus	0.0008421
Stromal cells in Kidney	0.001193
Stromal cells in Heart	0.009271
Stromal cells in Adrenal	0.009271
Stellate cells in Liver	0.009271
Stromal cells in Lung	0.01401
Vascular endothelial cells in Thymus	0.02486
Stromal cells in Muscle	0.03563

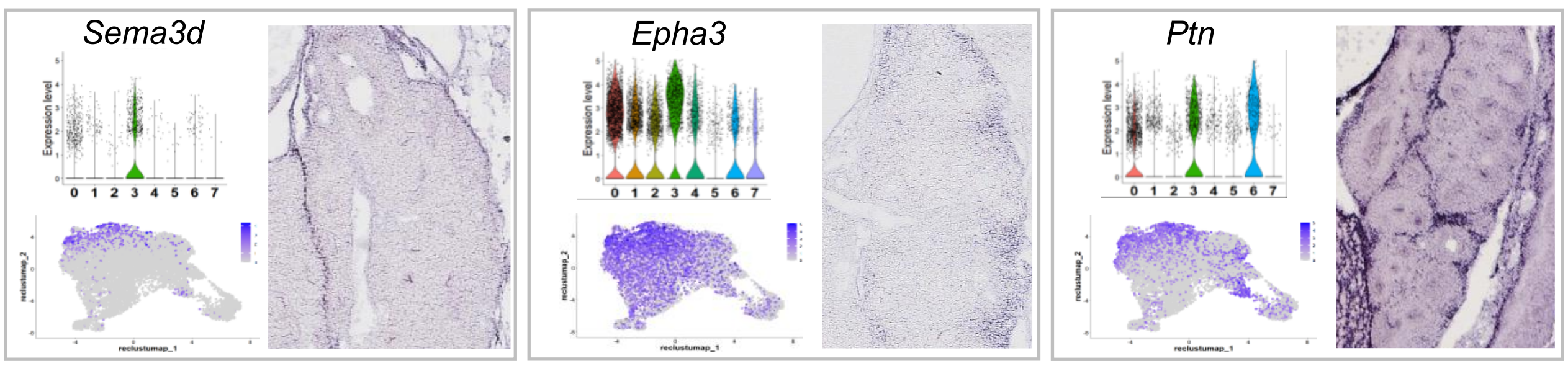

B

Gene	AvgLog2FC	Adj p value ↑
Rspo2	1.52	6.6×10^{-75}
Arhgap20	1.61	6.9×10^{-75}
Hcn1	2.17	1.1×10^{-74}
Adam12	0.94	1.7×10^{-74}
Dlgap1	2.54	1.2×10^{-67}
Ctnna2	1.78	4.4×10^{-65}
Dcx	3.28	1.2×10^{-64}
Hpse2	0.97	9.1×10^{-64}
Plxdc2	0.91	8.6×10^{-59}
Hs6st3	0.77	4.5×10^{-57}
Shisa9	2.61	2.7×10^{-56}
Unc5c	0.53	4.2×10^{-56}
Meg3	0.70	1.6×10^{-51}
Plagl1	1.36	3.1×10^{-49}
Itpr1	1.38	5.5×10^{-49}
Kcnk2	1.66	5.4×10^{-47}
C1qtnf7	1.05	9.2×10^{-47}
Tshz1	1.39	1.3×10^{-45}
Celf2	0.61	2.9×10^{-45}
Dlk1	0.63	8.1×10^{-44}
Meis2	1.01	5.1×10^{-43}
Adamts9	1.07	8.1×10^{-43}
Itga8	0.98	1.8×10^{-41}
Slit3	0.69	1.5×10^{-40}
Fgf10	1.17	2.6×10^{-39}
Capn6	1.86	4.0×10^{-39}
Rian	0.87	4.1×10^{-38}
Wnt2	1.21	2.3×10^{-35}

GO enrichment Cluster 3

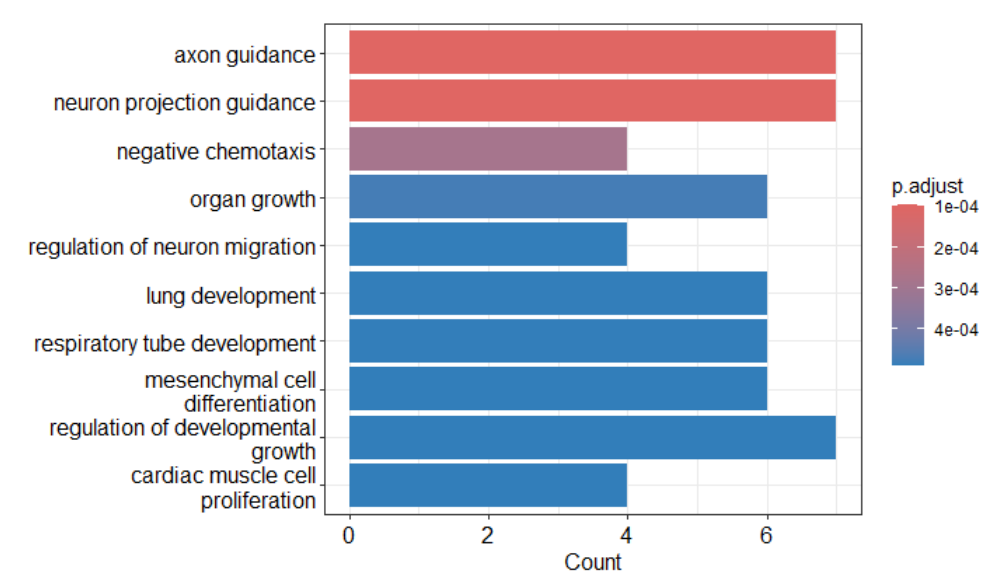

GO term	Genes
axon guidance	*Sema3d*, *Epha3*, *Sema3a*, *Alcam*, *Unc5c*, *Slit3*, *Epha7*
lung development	*Ccbe1*, *Rspo2*, *Meg3*, *Fgf10*, *Wnt2*, *Gata6*
mesenchymal cell differentiation	*Sema3d*, *Epha3*, *Sema3a*, *Fgf10*, *Rian*, *Wnt2*

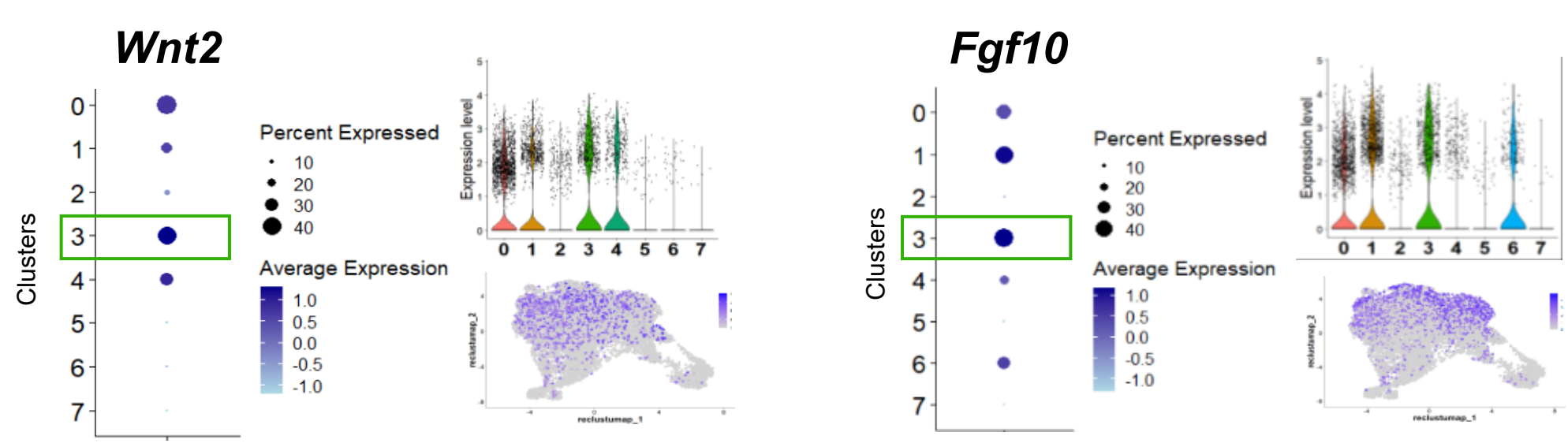

C

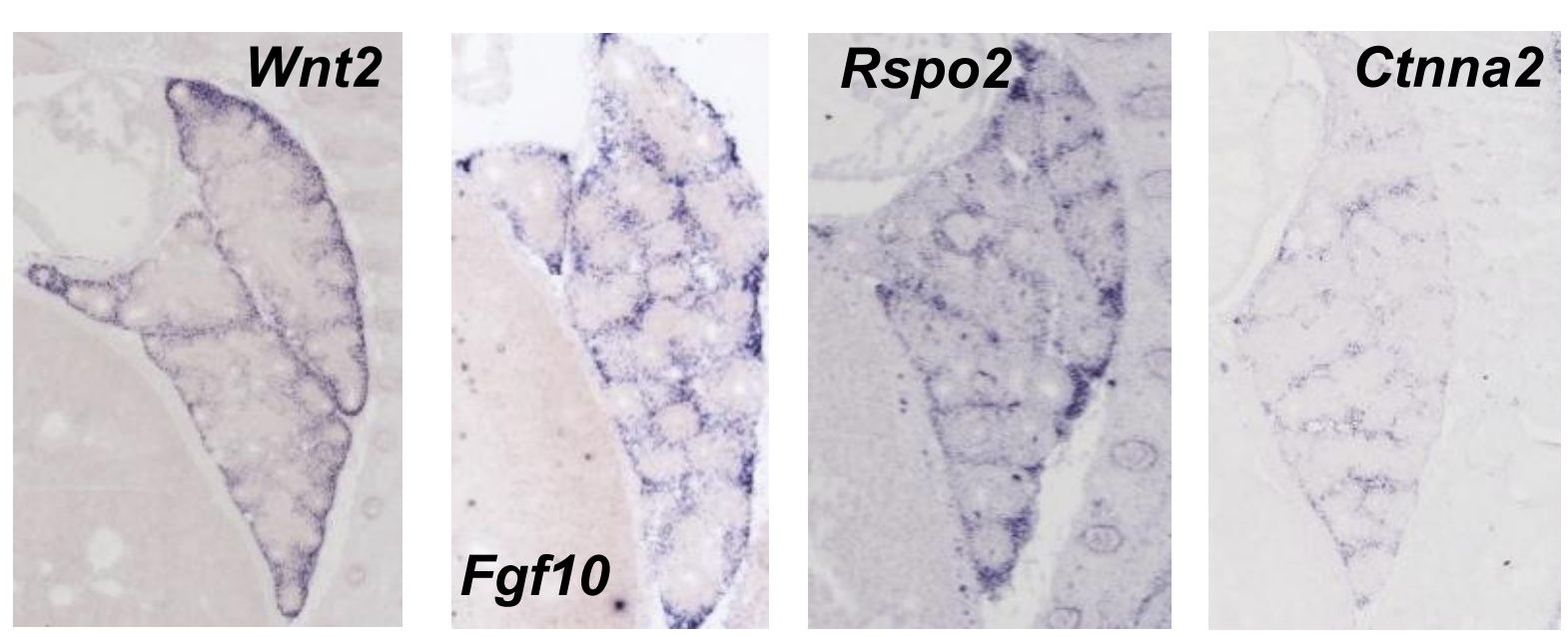

D

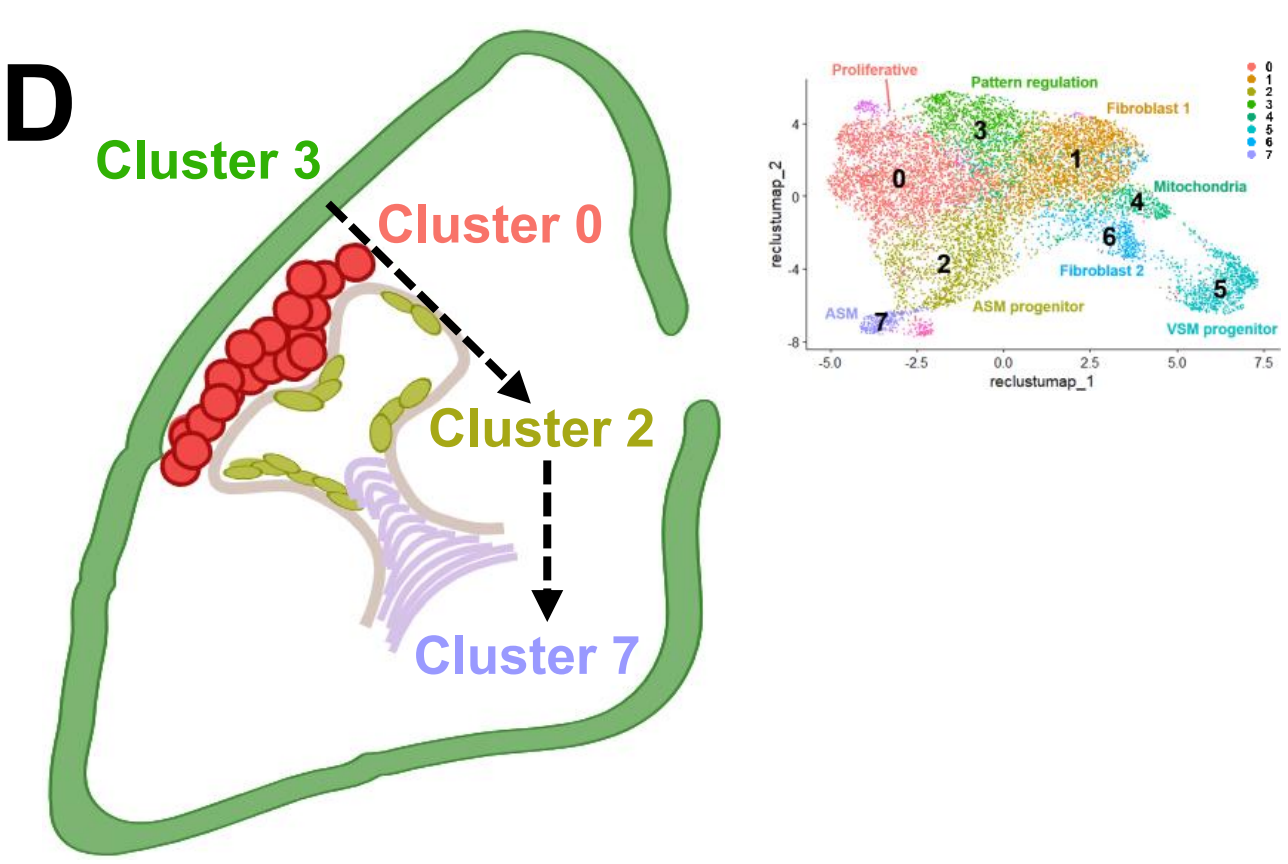

Suppl. Figure 7
Otoshi et al.

Cluster 2

A

GO Biological Process 2025

GO term ID	Term Name	Adjusted p value
GO:0051145	Smooth Muscle Cell Differentiation	0.006429
GO:0045445	Myoblast Differentiation	0.006429
GO:0097485	Neuron Projection Guidance	0.006429
GO:0045879	Negative Regulation of Smoothened Signaling Pathway	0.006429
GO:0001656	Metanephros Development	0.006429
GO:0051155	Positive Regulation of Striated Muscle Cell Differentiation	0.006429
GO:0055007	Cardiac Muscle Cell Differentiation	0.006429
GO:0055002	Striated Muscle Cell Development	0.006429
GO:0007411	Axon Guidance	0.006429
GO:0001501	Skeletal System Development	0.006732

Cluster 7

B

GO Biological Process 2025

GO term ID	Term Name	Adjusted p value
GO:0006940	Regulation of Smooth Muscle Contraction	0.0009560
GO:0006937	Regulation of Muscle Contraction	0.001447
GO:0090257	Regulation of Muscle System Process	0.008708
GO:1900221	Regulation of Amyloid-Beta Clearance	0.01341
GO:0031032	Actomyosin Structure Organization	0.01341
GO:0010976	Positive Regulation of Neuron Projection Development	0.01674
GO:0090287	Regulation of Cellular Response to Growth Factor Stimulus	0.01674
GO:0031346	Positive Regulation of Cell Projection Organization	0.01674
GO:0035088	Establishment or Maintenance of Apical/Basal Cell Polarity	0.01674
GO:0044331	Cell-Cell Adhesion Mediated by Cadherin	0.01674

A

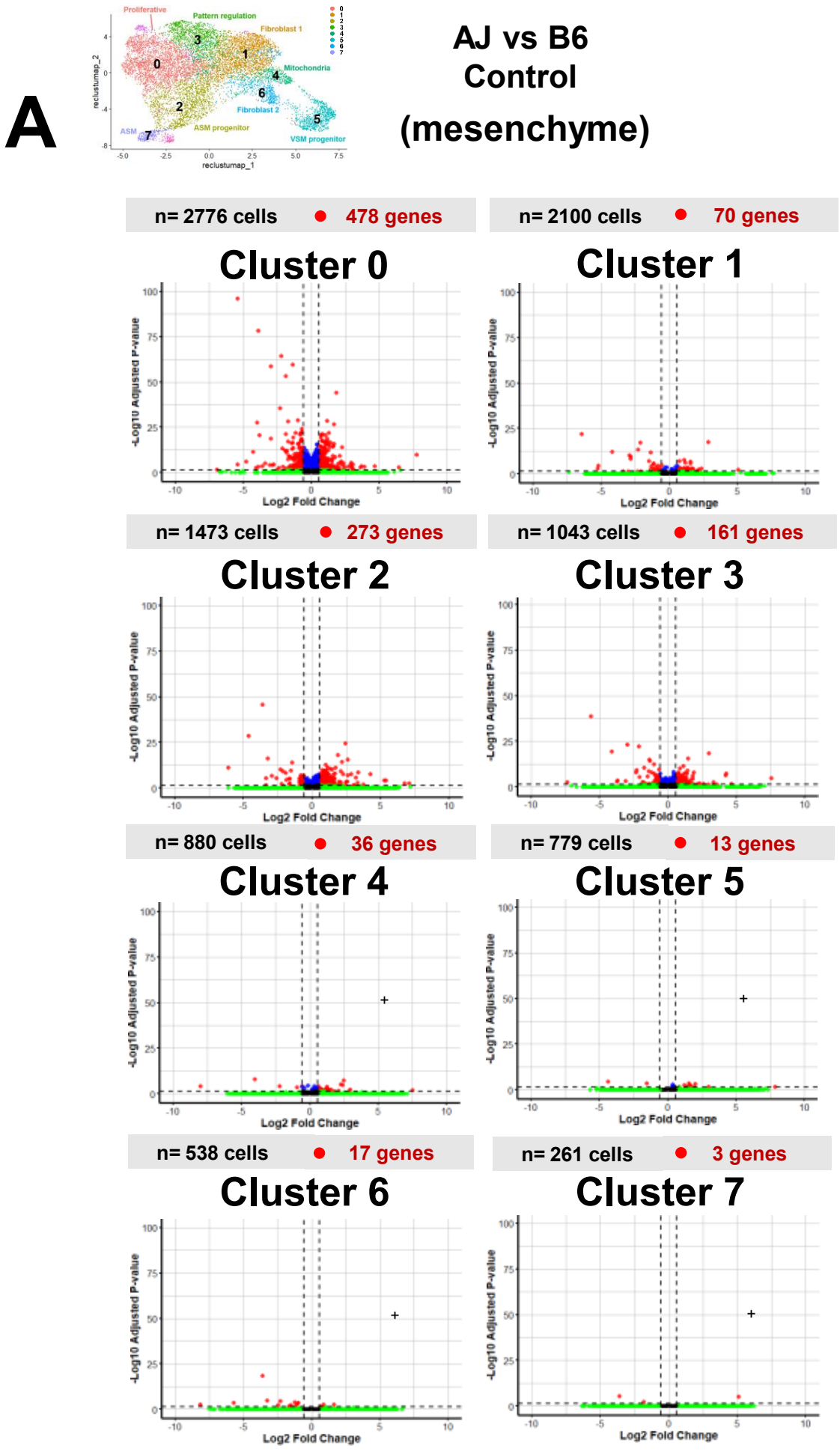

B

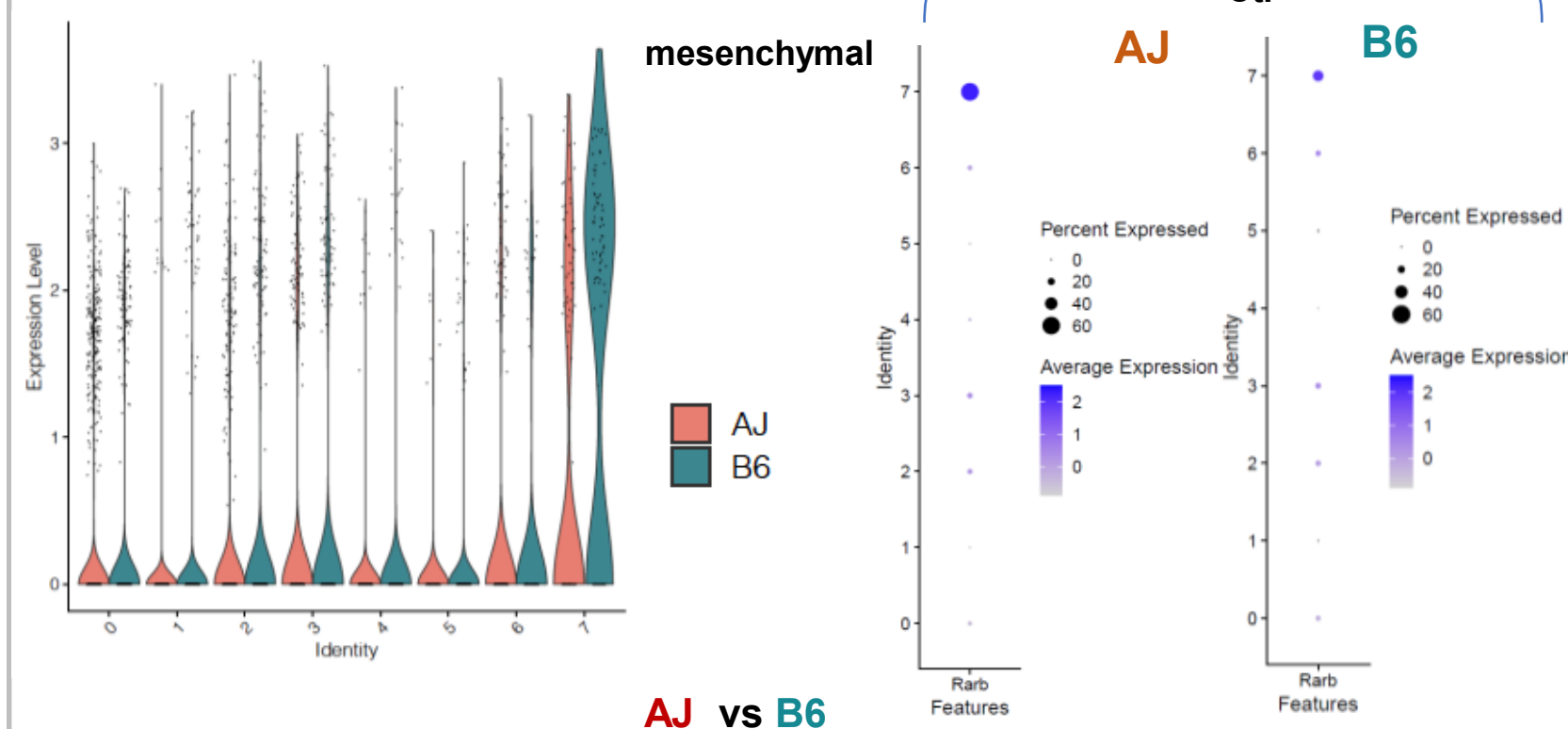

AJ vs B6 Control

Cluster 0

	avg_log2FC	pct.1	pct.2	p_val_adj
Rarb	0.37084952	0.209	0.132	0.259067

Cluster 1

	avg_log2FC	pct.1	pct.2	p_val_adj
Rarb	-0.343422	0.035	0.055	1

Cluster 2

	avg_log2FC	pct.1	pct.2	p_val_adj
Rarb	-0.4443896	0.217	0.176	1

Cluster 3

	avg_log2FC	pct.1	pct.2	p_val_adj
Rarb	0.1572566	0.239	0.169	1

Cluster 4

	avg_log2FC	pct.1	pct.2	p_val_adj
Rarb	-0.39676	0.101	0.068	1

Cluster 5

	avg_log2FC	pct.1	pct.2	p_val_adj
Rarb	0.6644587	0.085	0.05	1

Cluster 6

	avg_log2FC	pct.1	pct.2	p_val_adj
Rarb	0.8317866	0.25	0.149	1

Cluster 7

	avg_log2FC	pct.1	pct.2	p_val_adj
Rarb	-1.137191	0.349	0.648	1

C

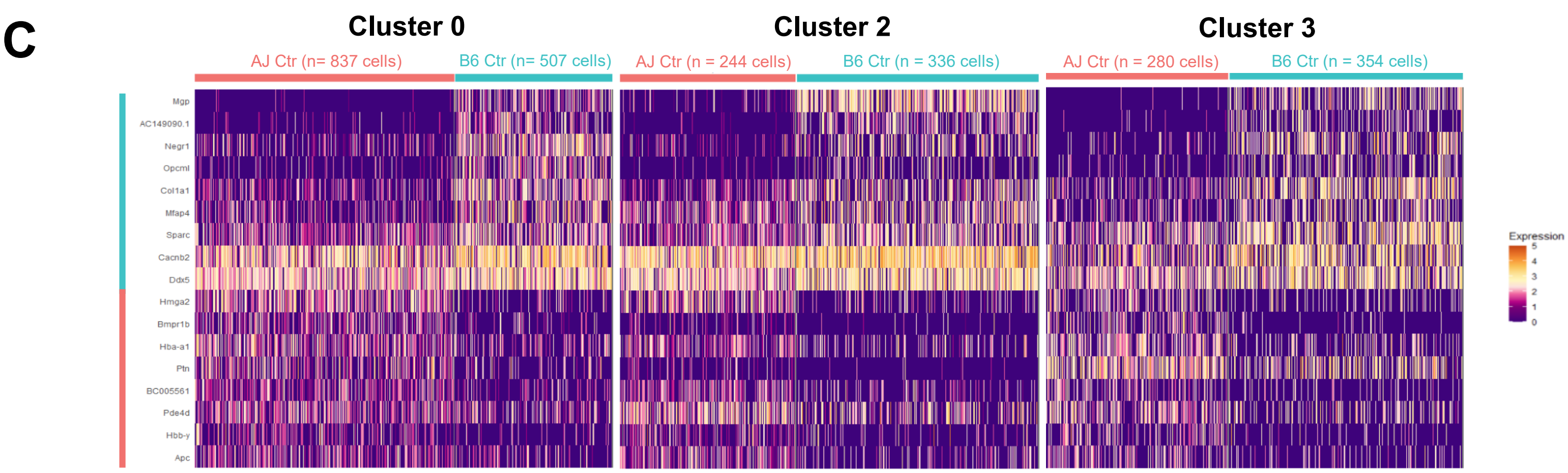

D

Cluster 0

Gene	AvgLog 2FC	Adj p value ↑
Mgp	-5.44	7.8×10^{-97}
AC149090.1	-3.92	2.9×10^{-79}
Negr1	-2.20	3.0×10^{-65}
Gm47283	-1.36	1.8×10^{-60}
Opcml	-2.96	1.7×10^{-59}
Col1a1	-1.90	3.7×10^{-54}
Hmga2	1.85	8.2×10^{-45}
Mid1	-2.30	2.4×10^{-36}
Colec12	-1.01	1.7×10^{-29}
Gm49708	1.18	2.8×10^{-29}
Mfap4	-1.70	4.1×10^{-29}
Myof	-3.98	2.4×10^{-28}
Bmpr1b	1.66	2.1×10^{-27}
Nisch	-0.70	7.9×10^{-25}
Ptprd	-0.69	3.6×10^{-23}
Hba-a1	1.29	8.8×10^{-23}
Slit2	-1.14	1.5×10^{-22}
Hjurp	-0.76	1.9×10^{-22}
Sparc	-0.82	2.9×10^{-22}
Elmo1	-1.89	4.6×10^{-22}
Gpc6	0.82	5.5×10^{-22}

Cluster 2

Gene	AvgLog 2FC	Adj p value ↑
Mgp	-3.59	1.9×10^{-46}
AC149090.1	-4.64	2.0×10^{-29}
Mpped2	2.41	3.1×10^{-25}
Hmga2	1.88	5.2×10^{-19}
Egfem1	-3.21	8.1×10^{-17}
Tmem158	2.60	3.2×10^{-16}
Sdk1	2.19	4.4×10^{-15}
Gm47283	-1.44	8.2×10^{-15}
Mir99ahg	1.00	1.5×10^{-13}
Mtcl1	1.16	6.4×10^{-12}
Kif26b	1.70	6.7×10^{-12}
Myof	-6.11	7.2×10^{-12}
Gm15261	1.32	3.2×10^{-11}
Kcnn2	1.48	6.6×10^{-11}
Rims1	-2.31	7.2×10^{-11}
Magi1	0.73	1.7×10^{-10}
Apc	1.02	1.8×10^{-10}
Prickle1	0.86	2.4×10^{-10}
Col1a1	-1.55	2.5×10^{-10}
BC005561	1.02	4.0×10^{-10}
Negr1	-2.15	5.7×10^{-10}

Cluster 3

Gene	AvgLog 2FC	Adj p value ↑
Mgp	-5.63	1.9×10^{-39}
Negr1	-2.98	7.6×10^{-24}
Col1a1	-2.13	6.2×10^{-23}
AC149090.1	-4.12	3.4×10^{-20}
Bmpr1b	3.00	4.1×10^{-19}
Gm49708	1.48	1.9×10^{-16}
Gm47283	-1.39	1.3×10^{-15}
Col3a1	-1.34	4.3×10^{-15}
Sparc	-1.05	3.4×10^{-13}
Gas5	1.11	3.3×10^{-12}
Dcn	-1.76	4.0×10^{-11}
Hba-a1	0.87	8.0×10^{-11}
Nisch	-0.67	3.6×10^{-10}
Opcml	-2.29	5.0×10^{-10}
Igfbp5	1.42	1.3×10^{-9}
Mfap4	-1.39	3.3×10^{-9}
Zfp292	0.39	6.3×10^{-9}
Foxp1	1.86	1.1×10^{-8}
Mid1	-2.32	1.3×10^{-8}
Col1a2	-1.19	1.4×10^{-8}
Tenm4	0.72	1.4×10^{-8}

E

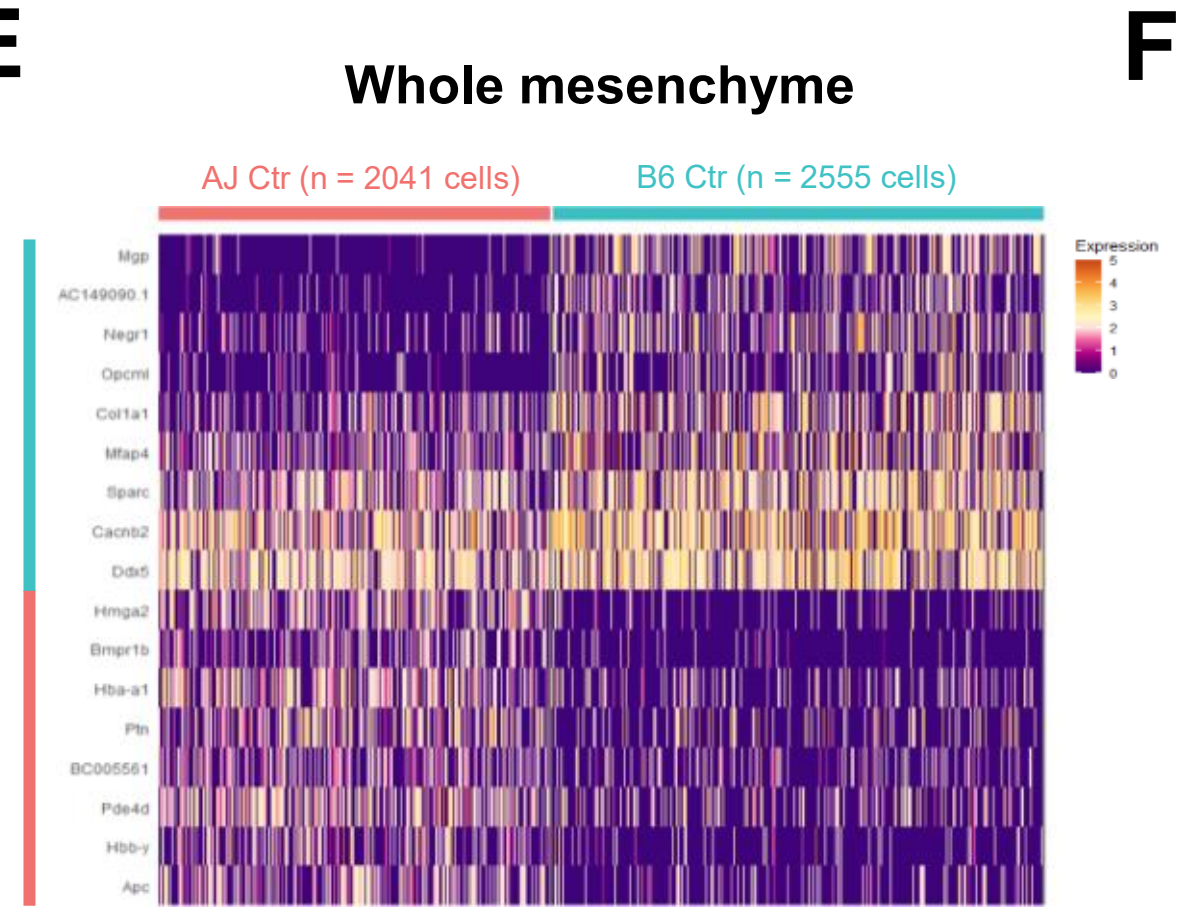

F

AJ Ctr vs B6 Ctr
Bulk RNA seq

Gene	AvgLog2FC	Adj p value ↑
Mgp	-2.99	4.0×10^{-251}
Opcml	-2.70	2.0×10^{-96}
Negr1	-2.04	3.0×10^{-72}
Ptn	0.80	3.8×10^{-32}
Mfap4	-0.73	5.9×10^{-19}
Col1a1	-0.75	1.0×10^{-15}
Hmga2	1.10	5.9×10^{-13}
Hbb-y	1.02	2.4×10^{-8}
Hba-a1	0.64	3.2×10^{-2}

Suppl. Figure 9
Otoshi et al.

A

Whole mesenchymal cells

AJ

BMS vs Ctr

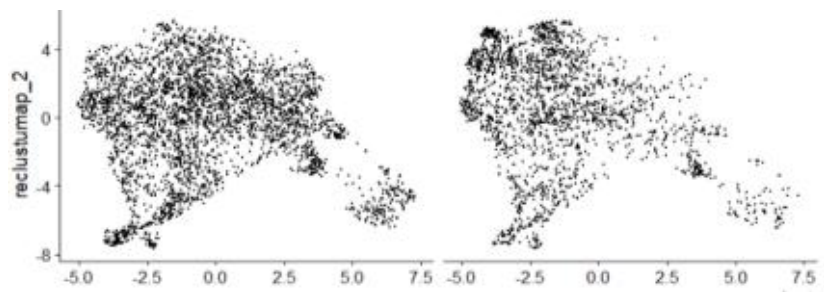

B6

BMS vs Ctr

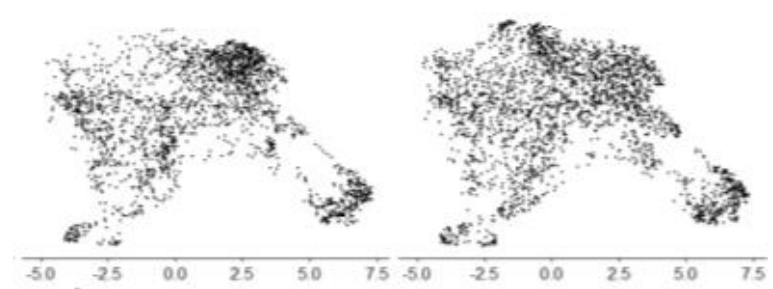

RA pathway

Gene	AJ AvgLog2FC	AJ Adj p value ↑	B6 AvgLog2FC	B6 Adj p value
Rarb	-1.74	5.7×10^{-57}	-1.18	1.0×10^{-14}
Dhrs3	-3.05	5.3×10^{-26}	-	Non-significant
Lrat	-3.97	2.0×10^{-7}	-	Non-significant

Tgfb pathway/ targets

Gene	AJ AvgLog2FC	AJ Adj p value ↑	B6 AvgLog2FC	B6 Adj p value
Col1a2	0.30	1.2×10^{-12}	-0.29	4.3×10^{-2}
Bgn	0.71	2.8×10^{-11}	-	Non-significant
Tgfbr3	0.15	1.7×10^{-7}	-	Non-significant
Ccn1	0.71	9.0×10^{-7}	-	Non-significant
Tgfbi	0.22	1.8×10^{-4}	-	Non-significant
Tgfbr2	0.12	2.3×10^{-4}	-	Non-significant

SM regulators

Gene	AJ AvgLog2FC	AJ Adj p value ↑	B6 AvgLog2FC	B6 Adj p value
Pdgfra	0.59	3.8×10^{-21}	-	Non-significant
Tnc	1.33	1.1×10^{-11}	0.89	6.2×10^{-6}
Entpd1	1.13	1.0×10^{-5}	-	Non-significant
Plxna4	1.32	1.4×10^{-5}	-	Non-significant
Zfp536	0.40	1.5×10^{-5}	-	Non-significant

SM markers

Gene	AJ AvgLog2FC	AJ Adj p value ↑	B6 AvgLog2FC	B6 Adj p value
Csrp1	0.44	3.5×10^{-5}	-	Non-significant
Myh11	0.52	9.1×10^{-3}	-	Non-significant

B

Pdgfra

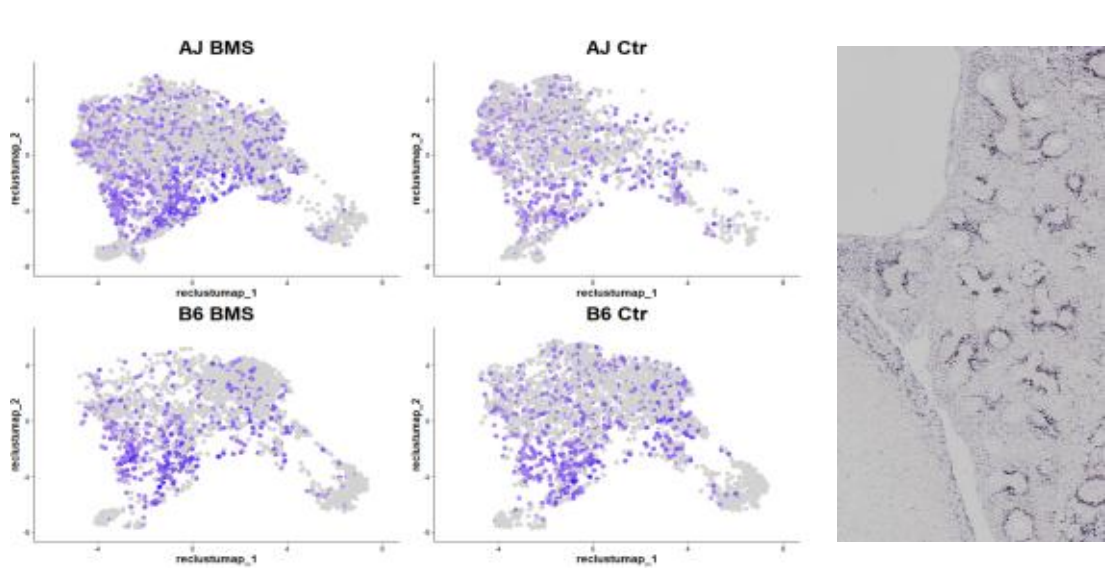

Entpd1

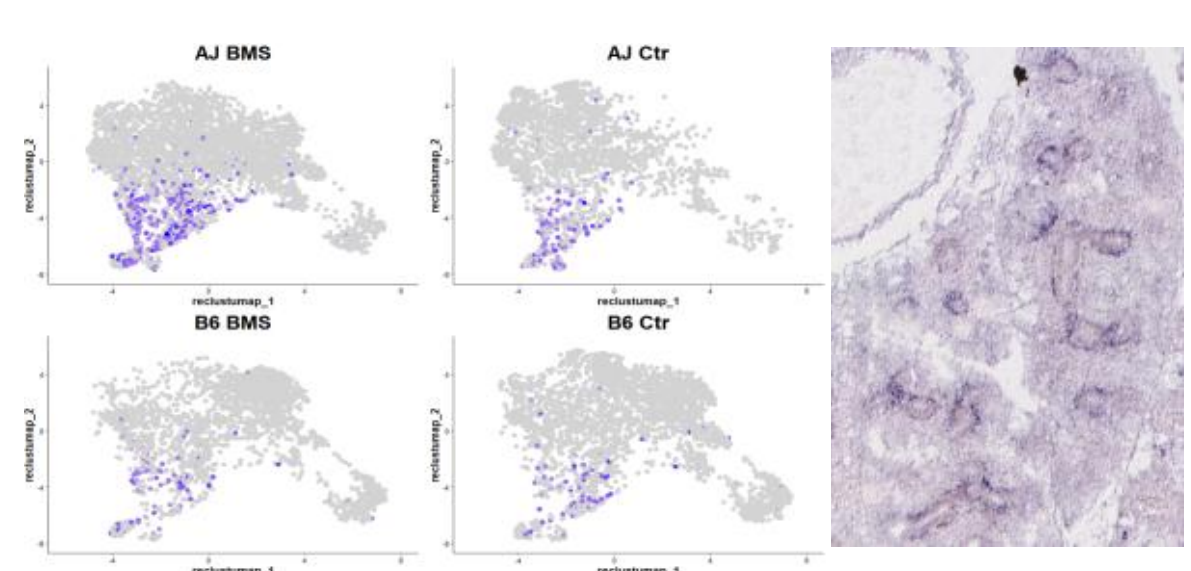

Bgn

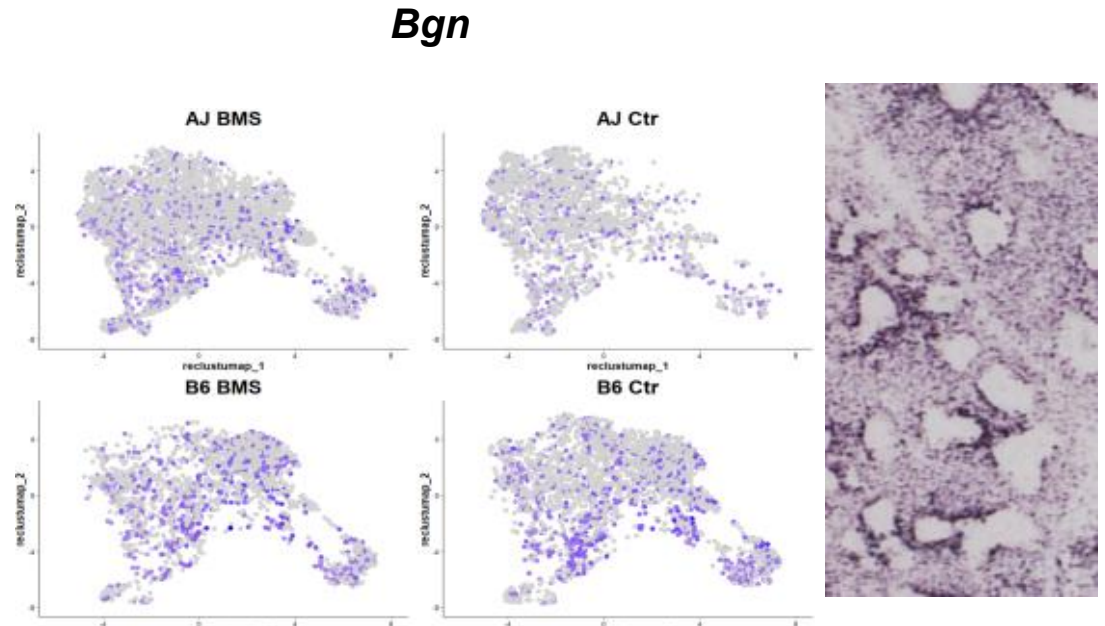

Ccn1

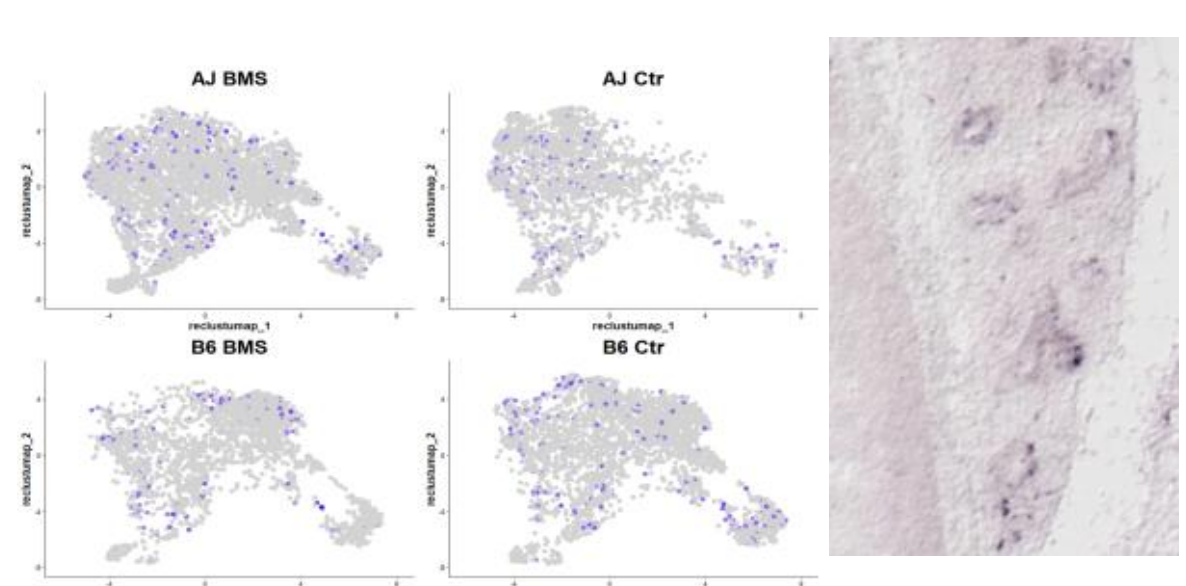

Suppl. Figure 10
Otoshi et al.

A

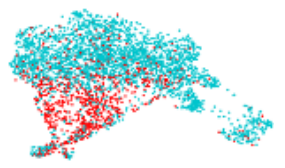

Tgfbi+ cells
(BMS vs Ctrl)
AJ

Top 50 (TOTAL = 337)

Gene	AvgLog2FC	Adj p value ↑
Ddx5	0.61	3.8×10^{-17}
Hmga2	-0.98	1.6×10^{-15}
Cd63	0.91	3.2×10^{-15}
Hbb-y	-2.51	1.1×10^{-13}
Epha7	-0.87	1.3×10^{-12}
Unc5c	-0.59	2.5×10^{-11}
Nr6a1os	-0.64	2.7×10^{-11}
Sema3a	-0.82	5.0×10^{-11}
Thsd4	-0.41	1.9×10^{-10}
Hecw2	-1.24	2.3×10^{-10}
Ccbe1	-2.54	2.9×10^{-10}
Pdgfra	0.94	4.7×10^{-10}
Meg3	-0.39	7.4×10^{-8}
2610307P16Rik	-0.55	9.1×10^{-8}
Spred1	-0.24	1.3×10^{-7}
Prpf40a	-0.55	1.4×10^{-7}
Hsp90b1	0.43	1.7×10^{-7}
Prex2	-0.83	2.7×10^{-7}
Tnc	1.67	2.7×10^{-7}
Bmpr1b	-0.81	4.0×10^{-7}
Pdzd2	-0.67	5.0×10^{-7}
Pdia3	0.34	6.8×10^{-7}
A630089N07Rik	-0.24	8.5×10^{-7}
Ppic	0.91	9.8×10^{-7}
Ptk2	-0.42	1.0×10^{-6}
Plagl1	-0.69	1.1×10^{-6}
Lrfn5	-3.29	1.9×10^{-6}
Itm2b	0.88	2.9×10^{-6}
Trim24	-0.40	3.2×10^{-6}
Dnmt3a	0.29	3.9×10^{-6}
Tshz3	-0.43	5.8×10^{-6}
Ccny	-0.29	5.9×10^{-6}
P3h2	-1.23	7.3×10^{-6}
Mfap4	0.65	7.5×10^{-6}
Tent2	-0.44	9.8×10^{-6}
Nisch	0.40	1.0×10^{-5}
Cyp7b1	-1.09	1.2×10^{-5}
Slit3	-0.51	1.3×10^{-5}
Hotairm1	-1.21	1.3×10^{-5}
Hba-a1	-1.40	1.5×10^{-5}
Laptm4a	0.39	1.7×10^{-5}
Sulf1	-1.06	1.9×10^{-5}
Rbm46	-0.87	2.0×10^{-5}
Dlc1	-0.29	2.1×10^{-5}
Slc25a36	-0.26	3.0×10^{-5}
Tnrc6a	-0.19	3.0×10^{-5}
Frmd4a	-0.25	3.0×10^{-5}
Ptbp2	-0.46	3.1×10^{-5}
Cd81	0.39	3.5×10^{-5}
Rspo2	-0.79	3.5×10^{-5}

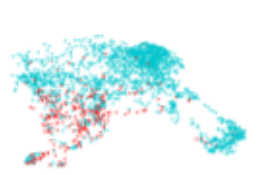

Tgfbi+ cells
(BMS vs Ctrl)
B6

TOTAL = 6

Gene	AvgLog2FC	Adj p value ↑
Serpinh1	-0.79	8.2×10^{-3}
Hspa5	-0.80	1.2×10^{-2}
Mdk	-0.60	1.3×10^{-2}
Ptprk	0.57	1.6×10^{-2}
1700018A04Rik	1.00	3.1×10^{-2}
Lcorl	0.48	3.7×10^{-2}

B

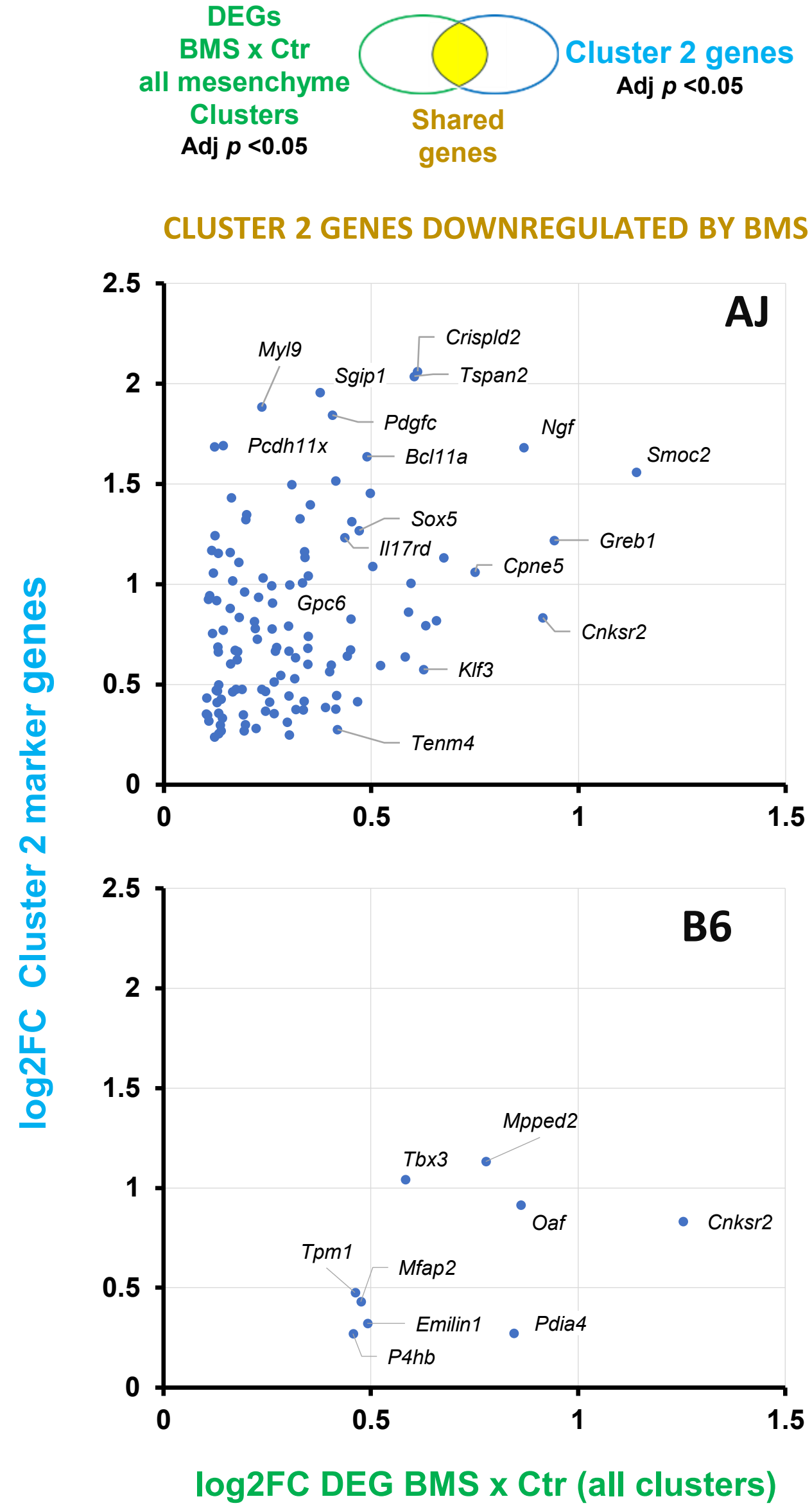

C

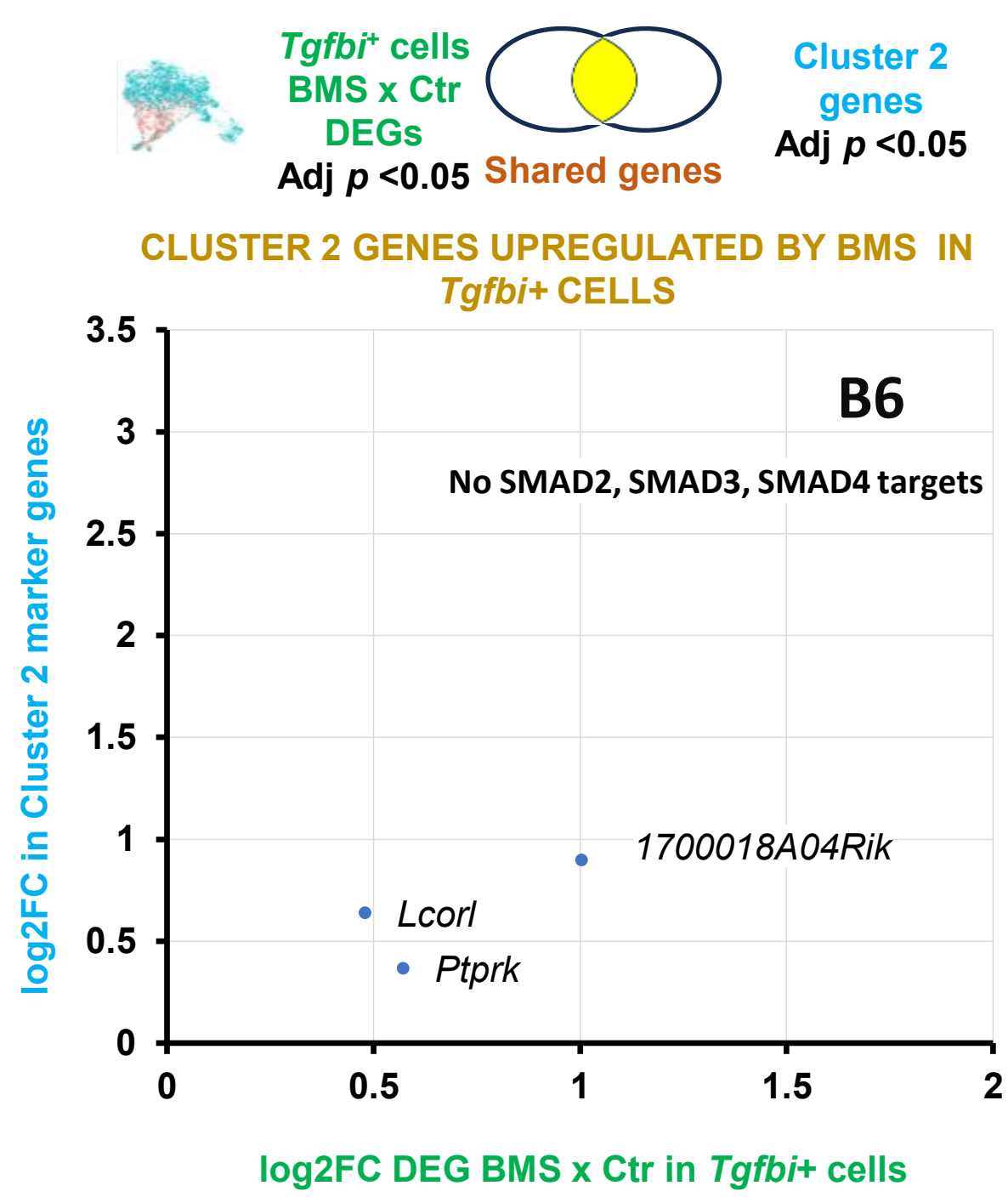

Suppl. Figure 11
Otoshi et al.

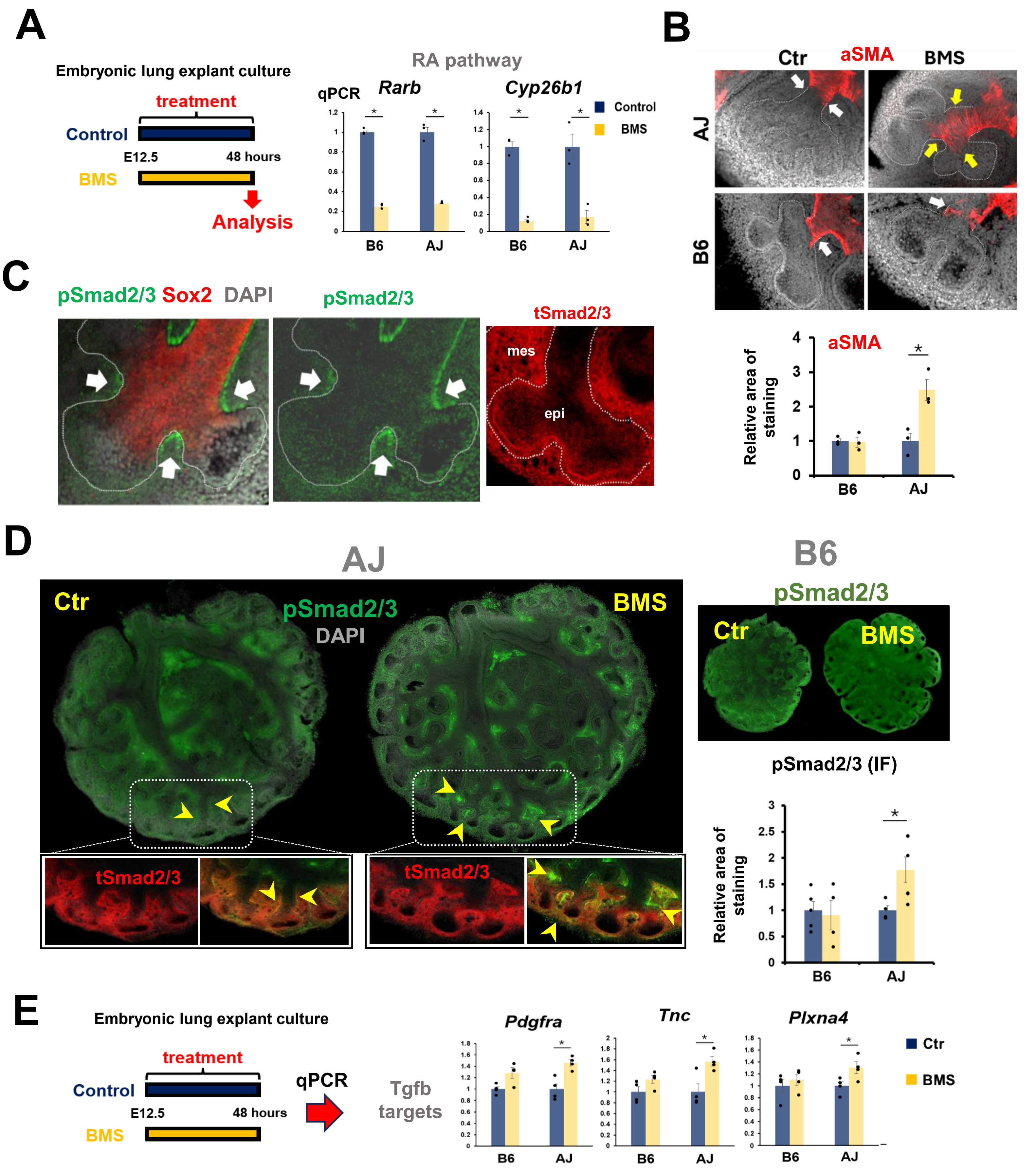

Suppl. Figure 12
Otoshi et al.

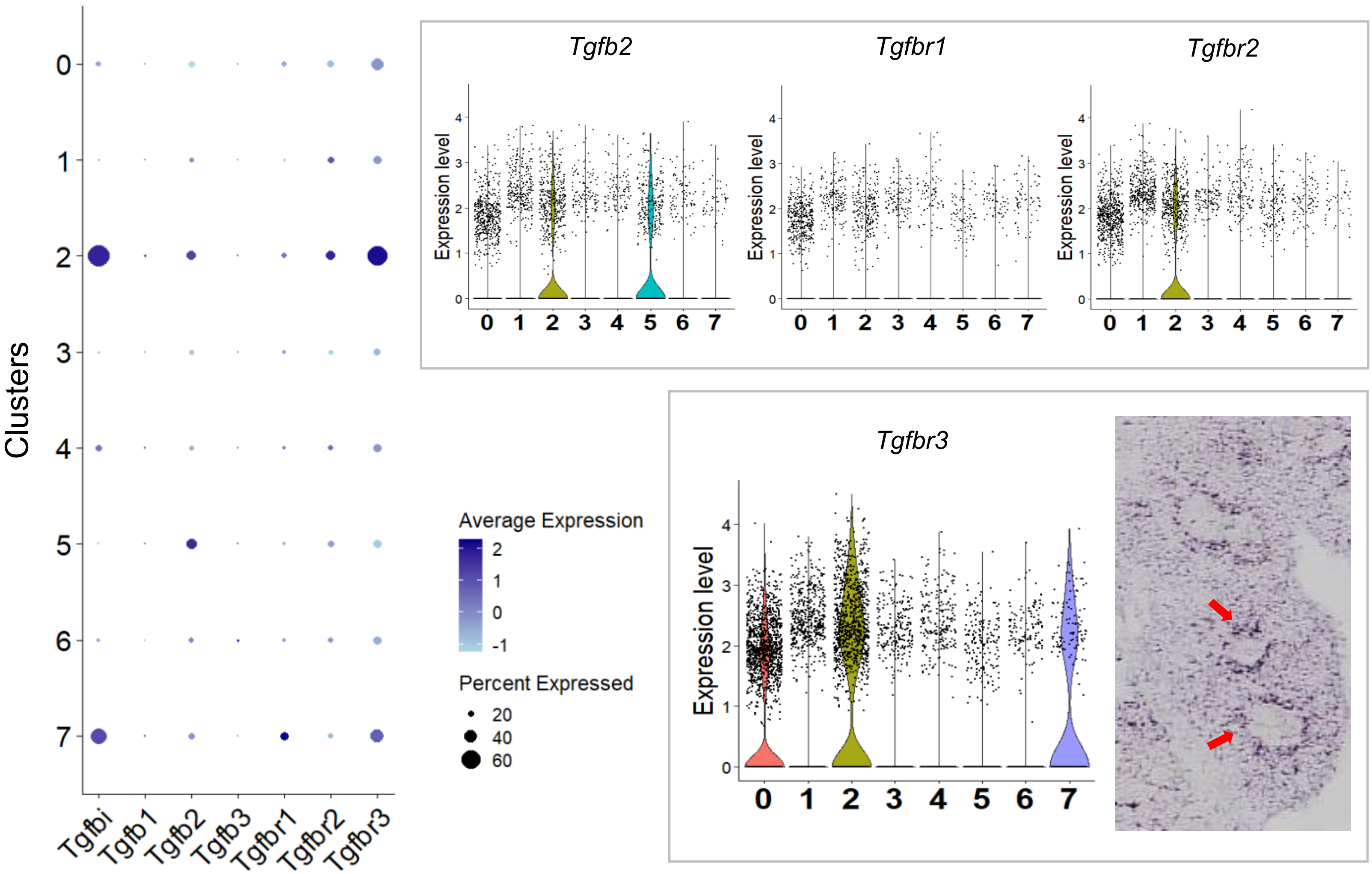

Suppl. Figure 13
Otoshi et al.

A

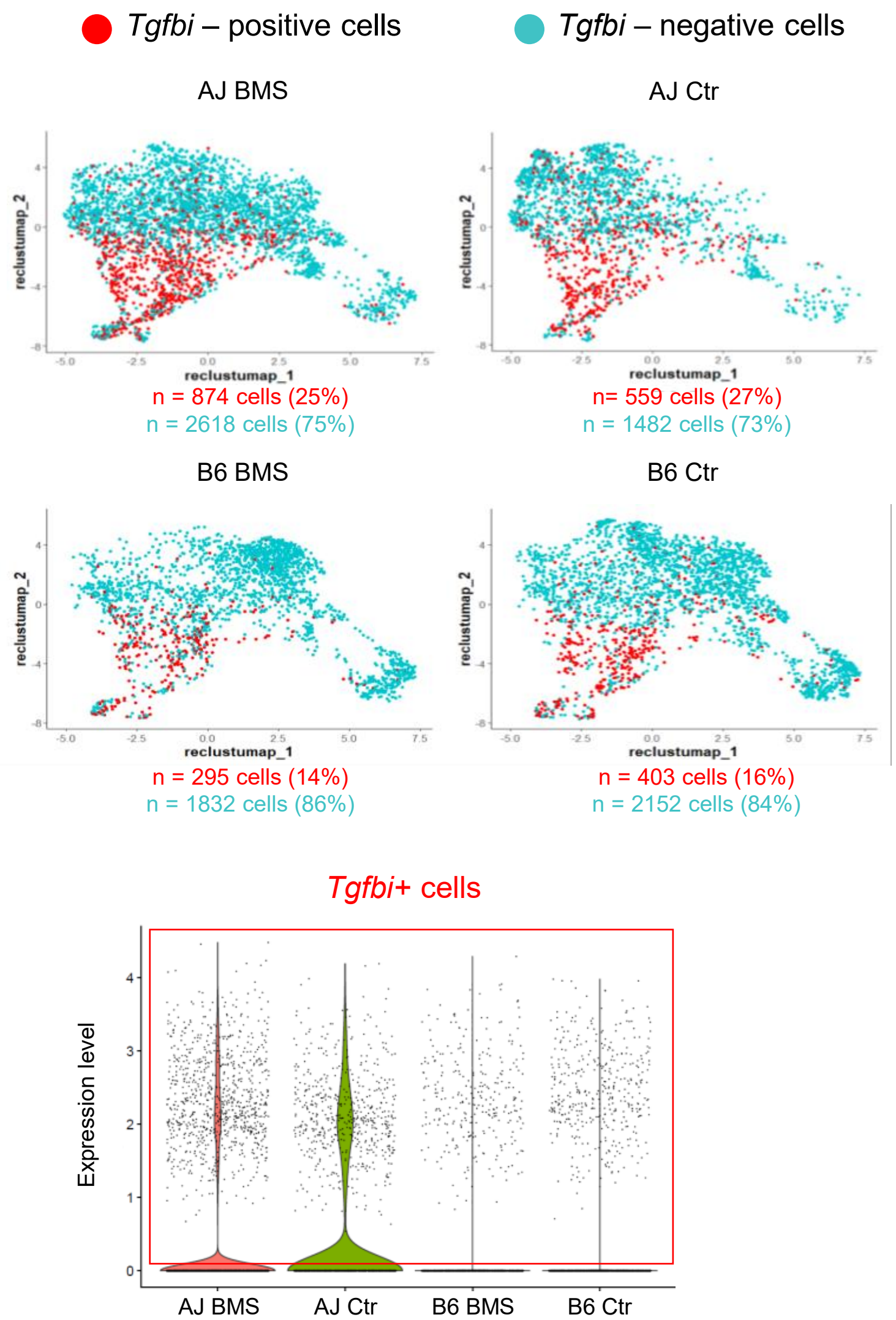

B

Tgfbi+ cells

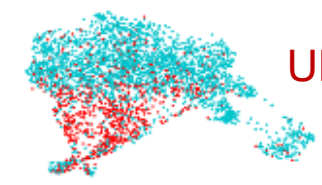

(BMS vs Ctrl)
UPREGULATED

AJ (79 DEGs) Cluster 2 markers

Gene	avg_log2FC ↑	p_adj
Tnc	1.67	$2.7x10^{-7}$
Plxna4	1.50	$7.0x10^{-3}$
Entpd1	1.24	$1.2x10^{-4}$
Emp3	1.12	$8.6x10^{-5}$
Pdgfra	0.94	$4.7x10^{-10}$
Ppic	0.91	$9.8x10^{-7}$
Cd63	0.91	$3.2x10^{-15}$
Itm2b	0.88	$2.9x10^{-6}$
Nrxn3	0.84	$2.8x10^{-2}$
1700018A04Rik	0.82	$5.6x10^{-3}$
Lamp1	0.71	$1.4x10^{-3}$
Mfap4	0.65	$7.5x10^{-6}$
Oaf	0.63	$1.4x10^{-3}$
Soga1	0.61	$1.5x10^{-2}$
Ddx5	0.61	$3.8x10^{-17}$
Ppib	0.61	$3.6x10^{-5}$
Rps3	0.58	$6.1x10^{-3}$
Rpl18a	0.56	$1.5x10^{-3}$
C430049B03Rik	0.55	$2.2x10^{-4}$
Mfap2	0.52	$6.3x10^{-4}$
Serpine2	0.51	$1.0x10^{-2}$
Lama2	0.50	$4.4x10^{-2}$
Prdm6	0.50	$3.7x10^{-2}$
Zfp536	0.49	$4.7x10^{-2}$
Ednrb	0.49	$3.0x10^{-3}$
mt-Co2	0.49	$3.7x10^{-2}$
Gas5	0.48	$3.7x10^{-4}$
Dad1	0.46	$3.8x10^{-2}$
Tmsb10	0.44	$3.0x10^{-2}$
Kif1b	0.43	$1.3x10^{-2}$
Hsp90b1	0.43	$1.7x10^{-7}$
Mylk	0.41	$1.0x10^{-2}$
Actb	0.41	$2.1x10^{-2}$
Peli2	0.41	$4.4x10^{-2}$
Nnat	0.40	$4.7x10^{-2}$
Nisch	0.40	$1.0x10^{-5}$
Laptm4a	0.39	$1.7x10^{-5}$
Cd81	0.39	$3.5x10^{-5}$
Tgfbi	0.35	$1.3x10^{-3}$
Pdia6	0.35	$4.2x10^{-2}$

B6 (3 DEGs)

Gene	avg_log2FC ↑	p_adj
1700018A04Rik	1.00	$3.1x10^{-2}$
Ptprk	0.57	$1.6x10^{-2}$
Lcorl	0.48	$3.7x10^{-2}$

C

	AJ		B6	
**Gene**	**AvgLog2FC ↓**	**Adj p value**	**AvgLog2FC**	**Adj p value**
Cyp7b1	-1.09	$1.3x10^{-5}$	-	Non-significant
Rarb	-0.84	$4.0x10^{-3}$	-	Non-significant
Dhrs3	-	Non-significant	-	Non-significant
Lrat	-	Non-significant	-	Non-significant

Suppl. Figure 14
Otoshi et al.

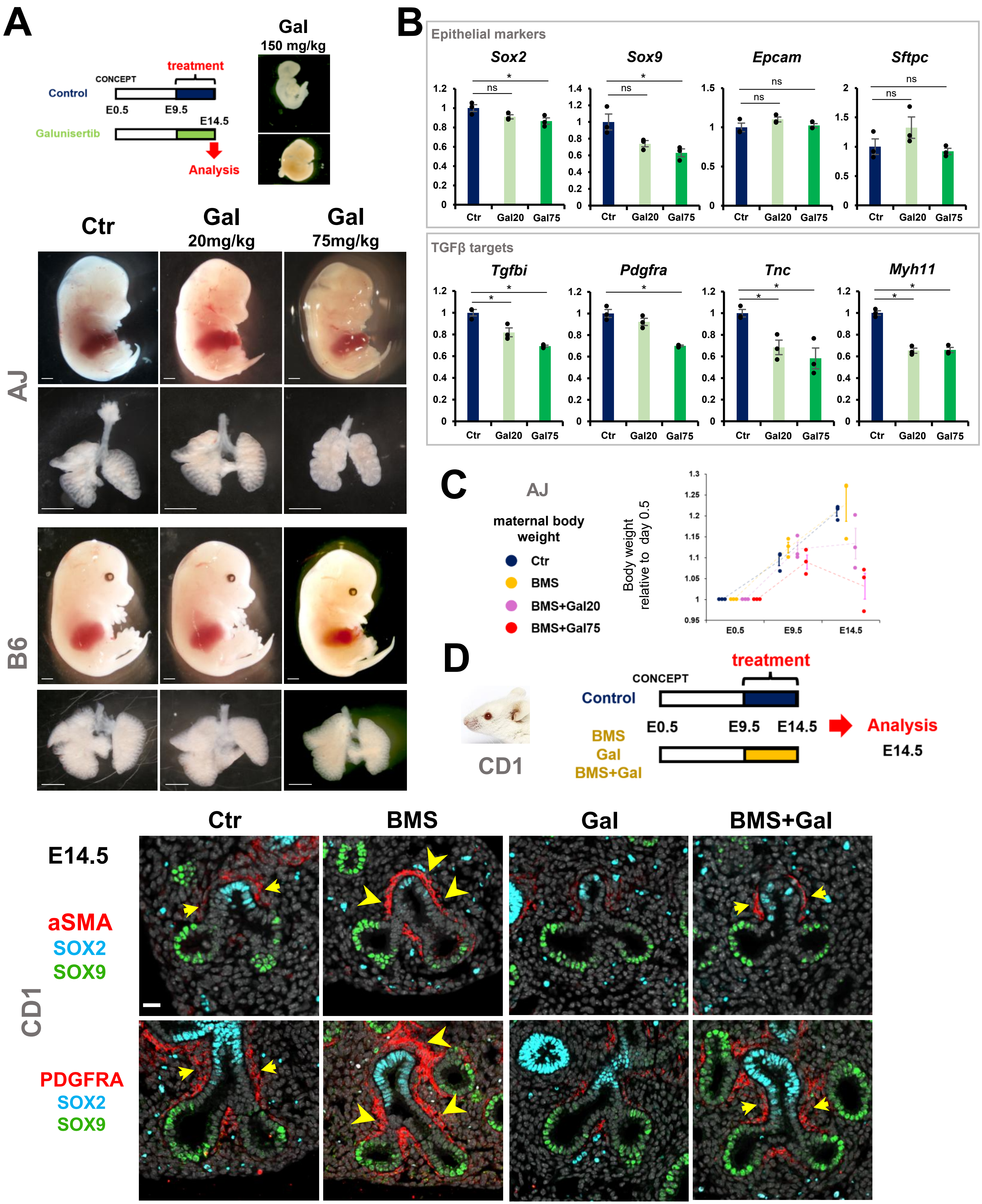

Suppl. Figure 15
Otoshi et al.

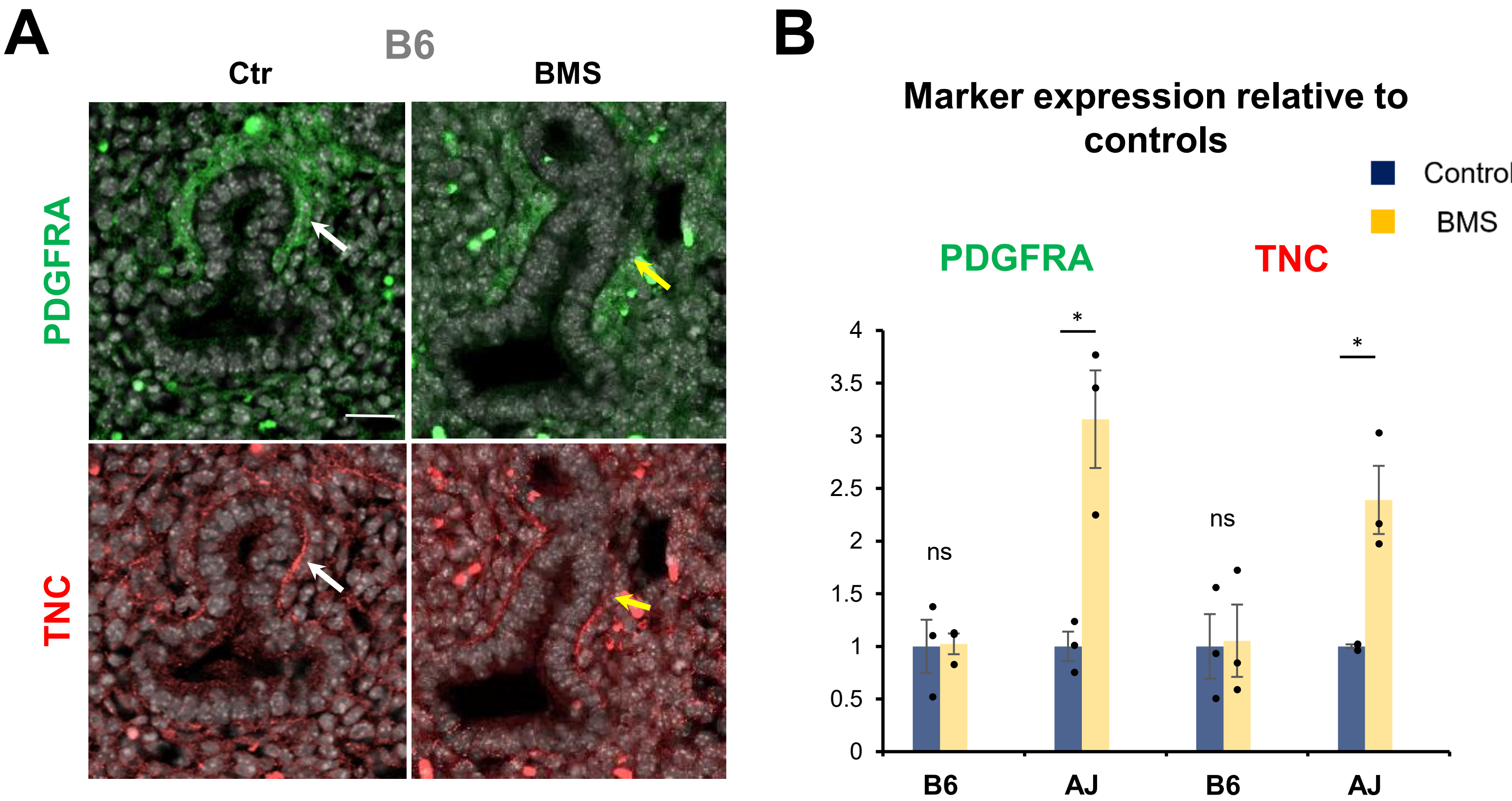

Suppl. Figure 16
Otoshi et al.

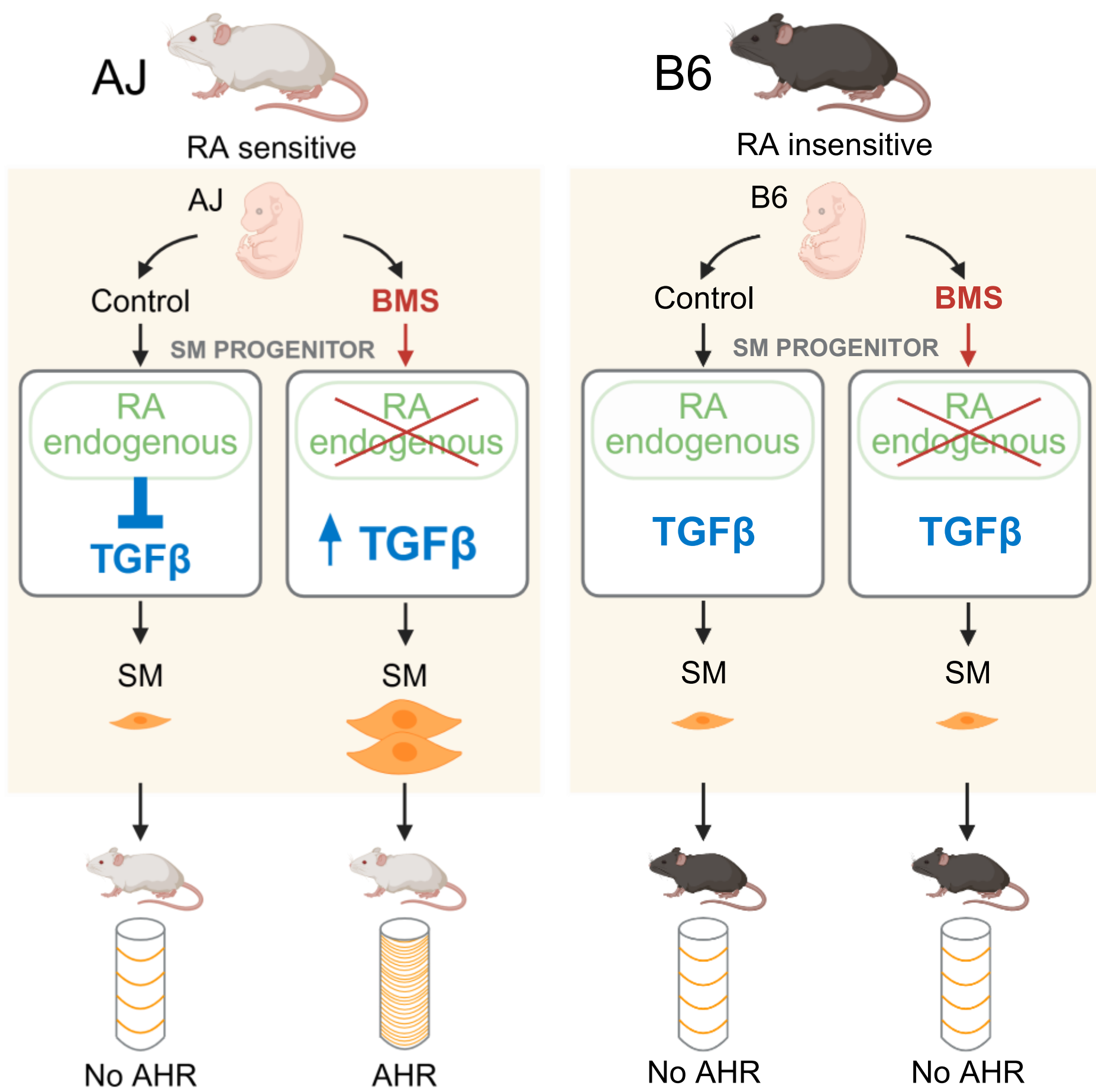

Suppl. Figure 17
Otoshi et al.
